# A Phenome-Based Functional Analysis of Transcription Factors in the Cereal Head Blight Fungus, *Fusarium graminearum*

**DOI:** 10.1371/journal.ppat.1002310

**Published:** 2011-10-20

**Authors:** Hokyoung Son, Young-Su Seo, Kyunghun Min, Ae Ran Park, Jungkwan Lee, Jian-Ming Jin, Yang Lin, Peijian Cao, Sae-Yeon Hong, Eun-Kyung Kim, Seung-Ho Lee, Aram Cho, Seunghoon Lee, Myung-Gu Kim, Yongsoo Kim, Jung-Eun Kim, Jin-Cheol Kim, Gyung Ja Choi, Sung-Hwan Yun, Jae Yun Lim, Minkyun Kim, Yong-Hwan Lee, Yang-Do Choi, Yin-Won Lee

**Affiliations:** 1 Department of Agricultural Biotechnology and Centers for Fungal Pathogenesis and Agricultural Biomaterials, Seoul National University, Seoul, Korea; 2 Department of Applied Biology, Dong-A University, Busan, Korea; 3 China Tobacco Gene Research Center, Zhengzhou Tobacco Research Institute, Zhengzhou, Henan, China; 4 Chemical Biotechnology Center, Korea Research Institute of Chemical Technology, Daejon, Korea; 5 Department of Medical Biotechnology, Soonchunhyang University, Asan, Korea; Purdue University, United States of America

## Abstract

*Fusarium graminearum* is an important plant pathogen that causes head blight of major cereal crops. The fungus produces mycotoxins that are harmful to animal and human. In this study, a systematic analysis of 17 phenotypes of the mutants in 657 *Fusarium graminearum* genes encoding putative transcription factors (TFs) resulted in a database of over 11,000 phenotypes (phenome). This database provides comprehensive insights into how this cereal pathogen of global significance regulates traits important for growth, development, stress response, pathogenesis, and toxin production and how transcriptional regulations of these traits are interconnected. In-depth analysis of TFs involved in sexual development revealed that mutations causing defects in perithecia development frequently affect multiple other phenotypes, and the TFs associated with sexual development tend to be highly conserved in the fungal kingdom. Besides providing many new insights into understanding the function of *F. graminearum* TFs, this mutant library and phenome will be a valuable resource for characterizing the gene expression network in this fungus and serve as a reference for studying how different fungi have evolved to control various cellular processes at the transcriptional level.

## Author Summary

Large collections of mutant lines allow for identification of gene functions. Here we constructed a mutant library of 657 putative transcription factors (TFs) through homologous recombination in the head blight fungus, *Fusarium graminearum*, providing a resource for understanding gene regulation in fungus. By screening these mutants in 17 phenotypic categories, we constructed a dataset of over 11,000 phenotypes. This study provides new insight into understanding multiple phenotypes caused by single TF as well as regulation of gene expression at the transcription level in *F. graminearum*. Furthermore, our TF mutant library will be a valuable resource for fungal studies through the distribution of mutants and easy access to our phenotypic and genetic data.

## Introduction

Transcription factors (TFs) orchestrate gene expression under the control of cellular signaling pathways and are key mediators of cellular function [1]. Understanding the regulatory mechanisms of each TF family and their function could provide valuable insight into gene expression changes underpinning cellular and developmental responses to environmental cues. Modification of TF activity through either gene disruption or overexpression can help determine the function and interconnectedness of individual TFs based on resulting cellular changes.

The filamentous fungus *Fusarium graminearum* (teleomorph: *Gibberella zeae*) is an important plant pathogen that causes head blight of major cereal crops, such as wheat, barley, and rice [2]. The fungus also produces mycotoxins in the infected cereals, posing grave threats to the health of animals and humans [3]. Complete genome sequencing of *F. graminearum* [4] has allowed for genome-wide gene functional studies, and transcriptome data from *F. graminearum* generated using the Affymetrix platform (http://www.plexdb.org/) can be used to predict gene function based on gene expression profiles [5], [6], [7], [8], [9], [10], [11], [12]. In addition, TFs from 64 fungal species, including *F. graminearum*, have been annotated in the Fungal Transcription Factor Database (FTFD, http://ftfd.snu.ac.kr/) [13]. The majority of *F. graminearum* TFs belong to the Zn(II)_2_Cys_6_ fungal binuclear cluster family; other types of TFs include C2H2 zinc finger, GATA, bHLH, B-ZIP, CBF, CCAAT-binding factor, homeobox, RING finger, PHD finger, and MIZ zinc finger [13]_ENREF_6. Although a few TFs that regulate pigmentation [14]_ENREF_6, mycotoxins biosynthesis [15], [16], [17]_ENREF_7_ENREF_7, sexual development, and virulence [18], [19], [20], [21], [22] have been characterized in *F. graminearum*, the function and regulation of most TFs remain to be determined.

In this study, we constructed *F. graminearum* deletion mutants in 657 genes encoding various types of TFs through homologous recombination to dissect their functions; resulting mutants were analyzed under 17 phenotypic categories to build a comprehensive phenotypic dataset (phenome). This phenome, which can be accessed at FgTFPD (*Fusarium graminearum* Transcription Factor Phenotype Database, http://ftfd.snu.ac.kr/FgTFPD), helped understand the mechanisms underpinning sexual and asexual development, toxin production, stress responses, and pathogenicity. The observed phenotypes were analyzed using a combination of data from published genome-wide fungal resources and bioinformatics analysis. Our mutant library will be a valuable source through distribution of mutants and easy access to our phenotypic and genetic data.

## Results

### Construction of genome-wide putative TF deletion mutants in *F. graminearum*

**Figure 1:**
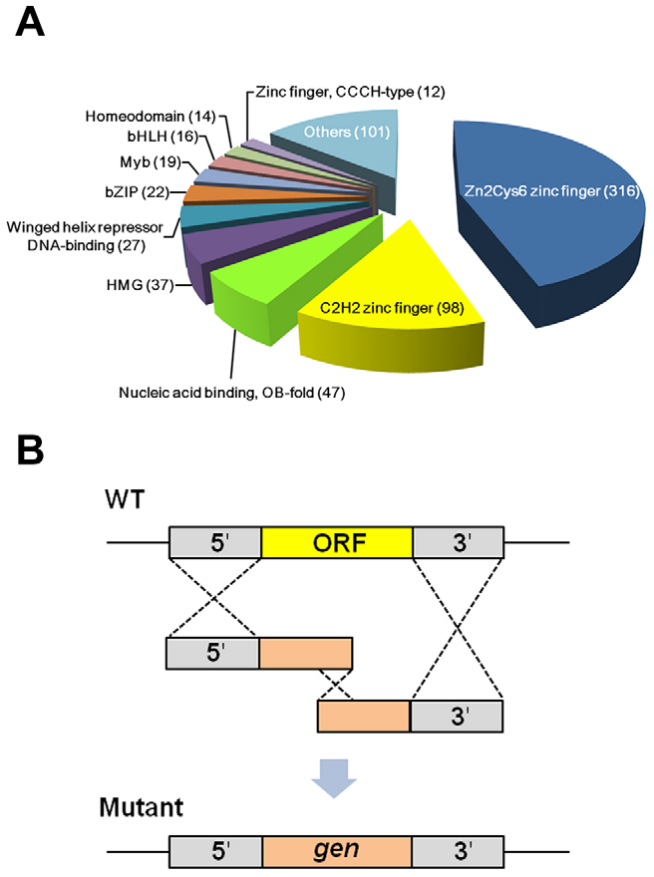
Classification and deletion strategy of putative transcription factors (TFs) in *Fusarium graminearum*. (A) Total TFs were classified based on nucleic acid binding domains. More than half of the total TFs are Zn_6_Cys_6_ zinc finger and C2H2 zinc finger proteins. (B) Each target TF gene was deleted using the split marker method based on triple homologous recombination.

In order to construct *F. graminearum* TF mutants, we employed the FTFD [13] to choose the genes annotated as TFs in *F. graminearum*. The FTFD is a standardized pipeline for annotating fungal TFs using the InterPro database that is based on DNA-binding motifs. Among the 64 classified TF families present in the 61 fungal and three oomycete species, 693 putative *F. graminearum* TFs, belonging to 45 families, were annotated and selected for deletion. We also added 16 manually identified TFs based on sequence homology or conserved motifs (Table S1). We finally selected 709 putative TFs, consisting of 6.1% of all the *F. graminearum* genes [4]_ENREF_6. It has been reported that TFs cover 3–6% of the genes in *Drosophila melanogaster*, *Caenorhabditis elegans*, and *Arabidopsis thaliana* [23]. In the fungal kingdom the percentage of TFs is also variable, ranging from 2.3% in *Pneumocystis carinii* to 7.2% in *Rhizopus oryzae* [13]. Approximately 60% of the total TFs in *F. graminearum* belong to TFs bearing zinc finger DNA-binding domains, including Zn(II)_2_Cys_6_, C2H2, and CCCH (Figure 1A and Table S2). In contrast, approximately 20% of TFs in *A. thaliana* and *C. elegans* belong to this group_ENREF_10. The higher percentage of zinc finger TFs in fungi is mainly due to the existence of fungal-specific Zn(II)_2_Cys_6_ zinc finger TFs (e.g., 25% of total TFs in *Saccharomyces cerevisiae*) [23]. In addition to the zinc finger-related TFs, the other major TF families in the *F. graminearum* genome include OB-fold nucleic acid binding, high mobility group, winged helix repressor DNA-binding, and B-ZIP domains containing proteins (Figure 1A).

Using homologous recombination, we successfully disrupted 657 TF genes out of 709 (Figure 1B). Eight genes were excluded because of failure to amplify their 5′ or 3′ flanking region under various PCR conditions and primer sets. Repeated attempts to delete the remaining 44 genes were unsuccessful likely due to lethality. Disruption of 132 genes was confirmed by co-dominant PCR and Southern hybridization with a 5′ or 3′ flanking region probe, as previously reported [24]. The remaining mutants were confirmed by co-dominant PCR screening followed by Southern hybridization with a geneticin resistant gene cassette (*gen*) probe (Figure S1). The *FgFSR1*-deletion mutant was generated in our previous study [21]. Only co-dominant PCR screening was used to confirm disruption of two TF mutants (FGSG_06071 and FGSG_10716). We found an extra copy of the mutant allele integrated ectopically in 49 mutants (Figure S1 and Table S2). We screened more than 10,000 transformants using co-dominant PCR to identify desired mutants, resulting in 2,183 positive mutants. Among them, approximately 65% were single deletion mutants without ectopic integration and 23% were deletion mutants with multiple integrations. In a high-throughput gene deletion study in *Neurospora crassa*, more than 90% deletion efficiency was obtained using a strain with deletion of genes required for non-homologous end-joining DNA repair (*mus-51* or *mus-52*) [25]. The split marker method based on triple homologous recombination to disrupt genes in *F. graminearum* was efficient enough that similar mutations of the parental strain were not required for mutagenesis.

### Phenotypic analyses of resulting mutants

**Figure 2:**
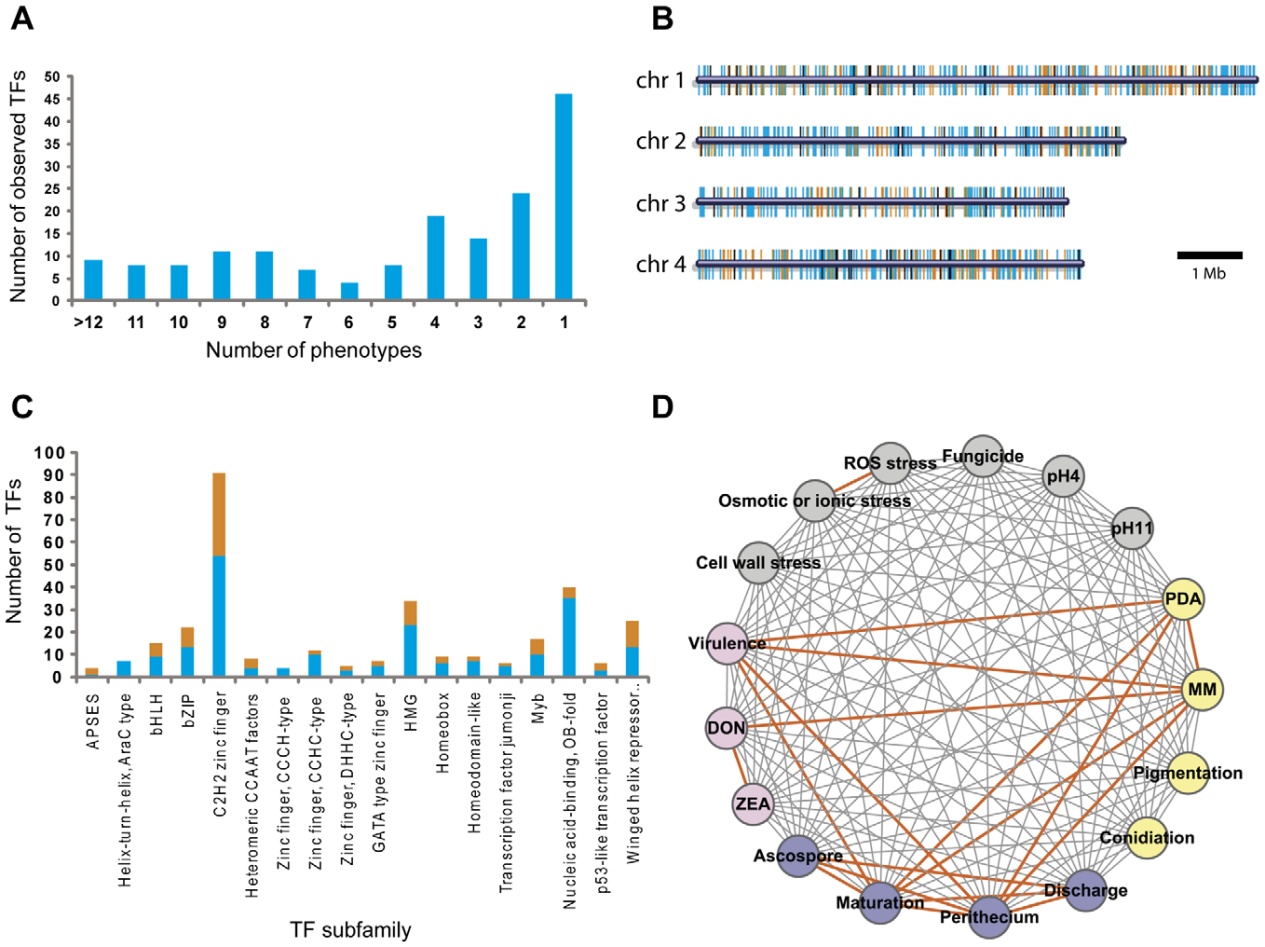
Analysis of transcription factor (TF) mutant phenotypes. (A) Number of TFs showing multiple mutant phenotypes. TFs without mutant phenotype were omitted. (B) Chromosome distribution of 709 putative TFs. TFs that exhibit mutant phenotypes (orange bars) and those that do not (blue bars) are randomly distributed throughout the genome. Black bars represent the TF genes that could not be disrupted. (C) Number of mutants showing mutant phenotypes in each TF subfamily. Orange and blue bars represent the number of TFs whose deletion results in a mutant phenotype or no phenotype change, respectively. Data representing the Zn_2_Cys_6_ subfamily are not shown in this graph. (D) Phenotype network based on PCC (Table S6). PCC values were calculated from the results of tested phenotypes. Orange edges indicate positive correlations (PCC>0.60).

We analyzed 657 TF mutants for changes in 17 phenotypes (Table S2), which fall into six major phenotypic categories including mycelial growth (Figure S2 and Figure S3), sexual development (Figure S4), conidia production (Table S3), virulence (Figure S5), toxin production (Figure S6), and stress responses (Figure S7). A substantial fraction of the mutants (26%, 170/657) displayed clearly visible mutant phenotypes, with 73% (124/170) of these mutants exhibiting multiple mutant phenotypes and 27% (46/170) with single mutant phenotype (Figure 2A, Table S4, and Table S5). Analysis of TF locations in the *F. graminearum* genome revealed that the 170 TFs exhibiting phenotype changes were randomly distributed throughout the genome (Figure 2B). Among 103 TF deletion mutants in *N. crassa* [25], 43% (44/103) exhibited phenotype changes with 50% (22/44) of these exhibiting multiple defects in growth and asexual/sexual development. Deletion of members of the Zn(II)_2_Cys_6_ TF family in *N. crassa*, resulted in mutant phenotypes in 42% (30/72) of the mutants [25]. However, we observed mutant phenotypes in only 16% (46/296) of the *F. graminearum* mutants defective in Zn(II)_2_Cys_6_ TFs. Deletion of the genes encoding winged helix repressor DNA-binding TFs resulted in the highest percentage of mutants with phenotype change (48%, 12/25), whereas Nucleic acid-binding, OB-fold TF deletion mutants displayed the lowest percentage of phenotype change (13%, 5/40) (Figure 2C and Table S4). The percentages of phenotype change among the mutants of the C2H2 zinc finger, Myb, and B-ZIP TF family members were 41% (37/91), 41% (7/17), and 41% (9/22), respectively.

To determine correlations between phenotypes, we calculated Pearson's correlation coefficient (PCC) from mutants that exhibit multiple mutant phenotypes (Table S6). The PCC between two phenotypes was calculated to determine which phenotypes are most highly correlated (Figure 2D). PCC>0.6 (*p* = 3.25×10^−12^) was chosen as the cut-off, and 18 phenotype pairs were selected and marked with orange edges between nodes (Figure 2D). This analysis revealed that sexual development, virulence, growth, and toxin production were highly correlated. The representative TFs are listed in Table 1. The phenotypes under sexual development, including perithecia formation and maturation, and ascospore production, were highly correlated with the phenotypes in other categories.

**Table 1:** List of key TF mutants showing no perithecia development and multiple defects in virulence, growth, and toxin production.

Locus ID	Gene name	Perithecia Development	Virulence	Growth on MM	DON production
FGSG_00477	*GzC2H003*	None	Reduced	Reduced	Normal
FGSG_10517	*GzC2H090*	None	Reduced	Reduced	Reduced
FGSG_13746	*GzNot002*	None	Reduced	Reduced	Increased
FGSG_10129	*FgStuA*	None	Reduced	Reduced	None
FGSG_10384	*GzAPSES004*	None	Reduced	Reduced	None
FGSG_06291	*GzBrom002*	None	Reduced	Reduced	None
FGSG_05171	*GzbZIP007*	None	Reduced	No growth	None
FGSG_13711	*GzC2H105*	None	Reduced	No growth	None
FGSG_10716	*GzCCHC011*	None	Reduced	No growth	None
FGSG_01665	*FgFSR1*	None	Reduced	Reduced	None
FGSG_09992	*GzNH001*	None	Reduced	Reduced	None
FGSG_13120	*GzOB047*	None	Reduced	Reduced	None
FGSG_06948	*Gzscp*	None	Reduced	Reduced	None
FGSG_08481	*GzWing018*	None	Reduced	Reduced	None
FGSG_08572	*GzWing019*	None	Reduced	Reduced	None
FGSG_08719	*GzWing020*	None	Reduced	Reduced	None
FGSG_08769	*GzZC108*	None	Reduced	Reduced	None
FGSG_07067	*GzZC232*	None	Reduced	Reduced	Normal
FGSG_06071	*GzAT001*	None	Reduced	Reduced	Normal
FGSG_01350	*GzC2H014*	None	Reduced	Reduced	Reduced
FGSG_06871	*GzC2H045*	None	Reduced	Reduced	Normal
FGSG_00324	*GzMyb002*	None	Reduced	Reduced	Reduced
FGSG_10069	*GzZC087*	None	Reduced	Reduced	Normal
FGSG_00574	*GzZC302*	None	Reduced	Reduced	Normal
FGSG_04220	*GzAPSES001*	None	Reduced	Reduced	Reduced
FGSG_01022	*GzC2H007*	None	Reduced	Normal	Reduced
FGSG_04134	*GzCON7*	None	Reduced	No growth	Reduced
FGSG_05304	*GzCCAAT004*	None	Reduced	Reduced	Reduced
FGSG_00385	*GzHMG002*	None	Reduced	Reduced	Reduced
FGSG_09339	*GzMADS003*	None	Reduced	Normal	Reduced
FGSG_12781	*GzMyb017*	None	Reduced	Reduced	Reduced

MM, minimal medium; DON, deoxynivalenol. Two TFs (*FgStuA* and *FgFSR1*) were characterized in the previous studies [19], [21].

We also performed in-depth studies of *F. graminearum* phenotypes under diverse environmental stresses. This analysis, including osmotic (or ionic), reactive oxygen species (ROS), fungicide, cell wall, and acidic (pH = 4) and basic (pH = 11) conditions, indicates there is no correlation between the stresses, except between osmotic and ROS stress (Figure 2D and Table S6).

We identified the *F. graminearum* orthologs of the 103 TF genes studied in *N. crassa* [25] to compare the resulting phenotypes between these species (Table S7A). Among 88 *F. graminearum* TFs identified, 26% of the mutants (23/88) exhibited mutant phenotypes with 43% (10/23) of these mutants showing multiple defects in growth and asexual/sexual development. However, only nine mutants showed the similar phenotype changes in growth, asexual, and/or sexual development in both species (Table S7B). This comparison of mutant phenotypes between *F. graminearum* and *N. crassa* suggests that the large majority of these TFs evolved to have unique, rather than conserved, functions in controlling growth and/or asexual and sexual developments.

### Classification of TFs associated with sexual development

**Figure 3:**
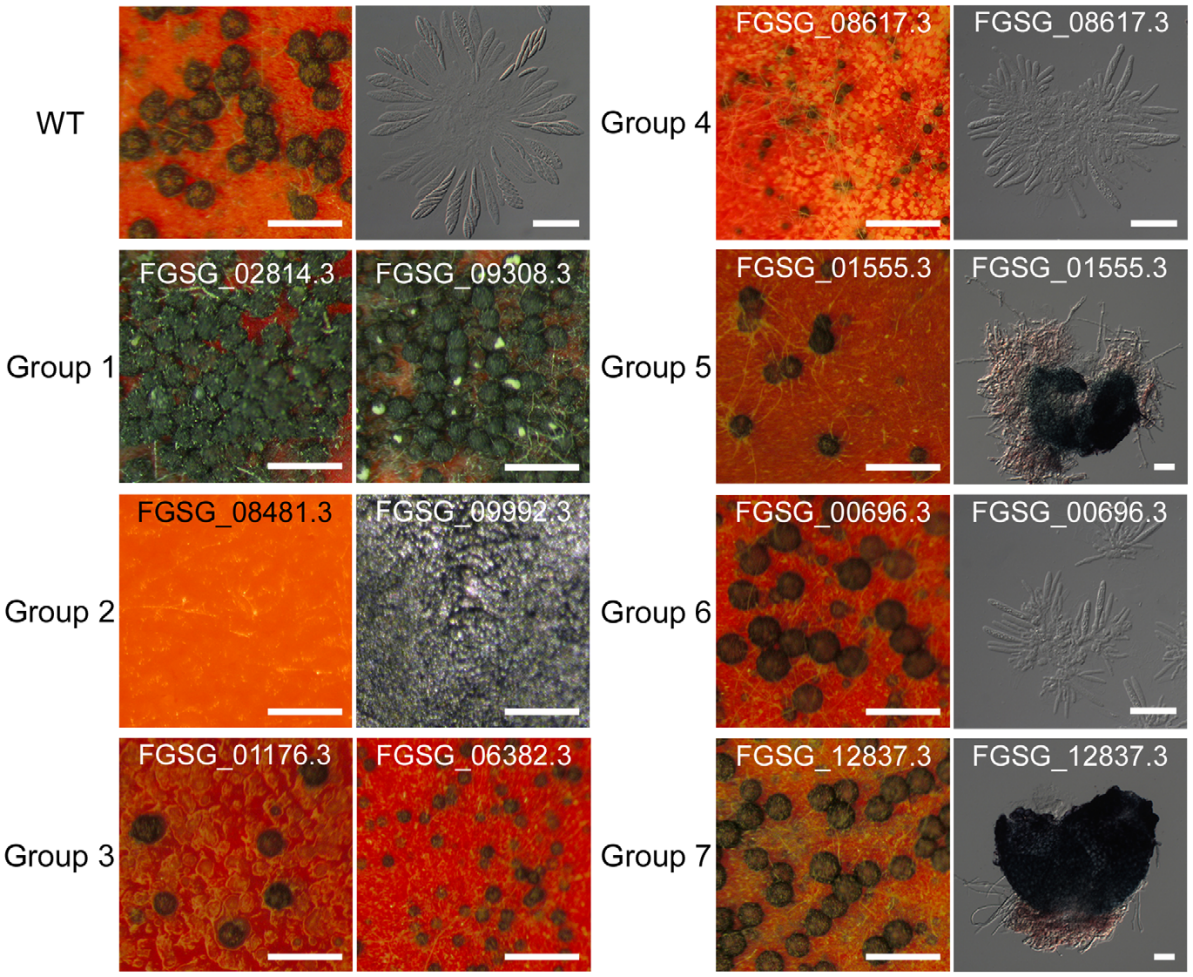
Representative images of wild-type or transcription factor (TF) mutants. TF mutants are defects in sexual development 10 days after sexual induction using dissecting or differential interference contrast (DIC) microscopy. Scale bar = 0.5 mm (left panel in wild-type, for dissecting microscopic pictures), 40 µm (right panel in wild-type, for DIC images).

Among the 170 TF mutants exhibiting at least one mutant phenotype, 105 mutants showed considerable defect in sexual development. Based on the nature and severity of the phenotype change in perithecia development (number of perithecia and perithecia maturation) and ascospore production (existence and morphology) among these 105 mutants, we classified them into seven groups representing (Table 2 and Table S8). Four mutants produced more perithecia than the wild-type strain (Group 1). Group 2 contained 44 mutants that completely lack perithecia development and could not produce any initial structure for perithecium. The mutants showing decreased number of perithecia or delayed perithecia maturation, which eventually developed normally, were further divided into three groups based on ascospore formation: normal-shaped ascospore formation (23 TFs, Group 3), abnormal-shaped ascospore (9 TFs, Group 4), and no ascospore formation (19 TFs, Group 5). Among the Group 5 mutants, 12 TF mutants (*GzCCAAT002*, *GzHMG010*, *MYT1*, *GzOB038*, *FgFlbA*, *GzWing015*, *GzRFX1*, *GzWing027*, *GzZC246*, *MAT1-1-3*, *MAT1-1-1*, and *MAT1-2-1*) formed protoperithecia but failed to differentiate further and the other 7 TF mutants belonging to Group 5 still made initial structures of asci or rosettes (Figure S4). Five of the six mutants with normal perithecia development produced abnormally-shaped ascospores (Group 6), and one mutant produced neither asci nor ascospores (Group 7) (Table 2 and Figure 3). In total, 96 mutants had defects in perithecia development, five mutants exhibited defects in ascospore production with normal perithecia development, and four mutants showed enhanced sexual reproduction.

**Table 2:** Classification of transcription factors (TFs) that are crucial for sexual development.

Group	Perithecia development	Ascospore formation	Number
1	Increased number	Normal	4
2	None	None	44
3	Defective	Normal	23
4	Defective	Abnormal shaped	9
5	Defective	No ascospore	19
6	Normal	Abnormal shaped	5
7	Normal	No ascospore	1

Groups were classified based on perithecia development (number or maturation) and ascospore formation (existence and morphology). “Defective” in perithecia development means decreased number or defective maturation. “Number” represents the number of TFs included in each group.

### Relationship between phenotype, TF conservation, and TF expression level

**Figure 4:**
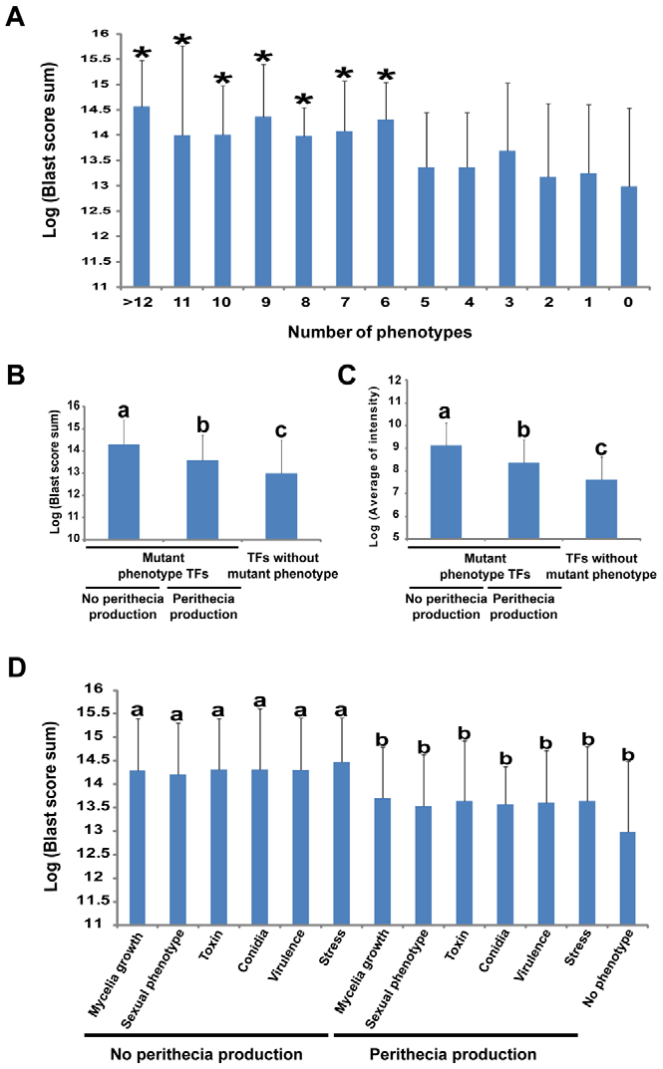
Relationship between multiple phenotypes, gene conservation in fungal kingdom, and transcription factor (TF) expression levels. (A) Relationship between BLAST score sum and number of phenotype changes caused by a single TF deletion. Statistical analysis was performed using the Fisher LSD test. *p<0.05. (B) Correlation between TF conservation and various phenotypes with and without perithecia formation. (C) Comparison of expression level and mutant phenotype TFs with and without perithecia formation. Statistical analysis for (B) and (C) was performed using the Duncan multiple range test. Values with different superscript letters are significantly different (*p*<0.01). (D) Relationship between BLAST score sum and the no perithecia formation phenotype. Statistical analysis was performed using the Duncan multiple range test. Values with different superscript letters are significantly different (*p*<0.05).

We utilized BLASTMatrix tool that is available on the Comparative Fungal Genomics Platform (CFGP, http://cfgp.riceblast.snu.ac.kr/) [26] to investigate whether TFs whose deletion resulted in a mutant phenotype are conserved in other fungal species. The distribution of each *F. graminearum* TF in other fungal species was surveyed using BLAST search scores. Total BLAST scores across the 164 species (159 fungi and five oomycetes) were obtained (Table S9). Higher scores for TFs imply that it is more distributed across fungal species. We divided the TF deletion mutants showing mutant phenotypes into subgroups based on the number of mutant phenotypes as well as phenotypic categories. The TFs whose mutants exhibited mutant phenotypes have higher BLAST scores than those TFs without mutant phenotypes (*p* = 1.01×10^−11^). In particular, TF mutants with more than six mutant phenotypes have significantly higher BLAST scores than those of TF mutants without a mutant phenotype (*p*<0.05, Fisher LSD test, Figure 4A and Table S10). Loss of TFs unique to *F. graminearum* showed mutant phenotypes in only 6% of the resulting mutants (2/31) compared to 26% of total tested *F. graminearum* TFs, and their expression levels were relatively low compared to the other 626 *F. graminearum* TFs. These unique TFs may function under specific conditions which we may not have tested in this study (Table S10).

Phenotypic analysis revealed that perithecia development was identified as central among the tested phenotypes. Thus, we examined whether TFs that show perithecia development defects when deleted are widely distributed in other fungal species. Interestingly, TFs whose deletion results in no perithecia production are more widely distributed in other fungal species than those of TF whose loss results in no phenotype change (*p*<0.01, Duncan multiple range test, Figure 4B). Furthermore, TFs whose deletion results in no perithecia production showed significantly higher BLAST scores than those of TF whose loss results in normal perithecia production (*p*<0.05, Duncan's multiple range test, Figure 4D).

The relationship between function and gene expression of *F. graminearum* TFs was examined through comparison of the phenotype dataset with the normalized spot intensity of *F. graminearum*. We downloaded 121 slides from pLEXdb (http://www.plexdb.org/) and normalized spot intensity to measure the expression level of each gene. The normalized spot intensity of TFs was calculated for each slide and then an average intensity was determined as the level of gene expression (Table S11). The TF mutants with mutant phenotypes had higher levels of gene expression than those of TF whose deletion results in no mutant phenotype (*p* = 1.60×10^−7^). Mutant phenotype TFs showed higher levels of gene expression than those of TFs whose deletion results in no mutant phenotype, and mutant phenotype TFs with no perithecia production exhibited higher levels of gene expression than those of mutant phenotype TFs with normal perithecia production (*p*<0.01, Duncan's multiple range test, Figure 4C). However, no different expression patterns were observed among the TFs with mutant phenotypes in sexual development even in perithecia development-related microarrays (Figure S8) [5], [6]. Overall, phenotype and expression level of TFs as well as phenotype and distribution of TFs in other fungal species is highly correlated with *F. graminearum* TFs.

### Interconnection of mutant phenotype TFs through phenotype, expression, and sequence

**Figure 5:**
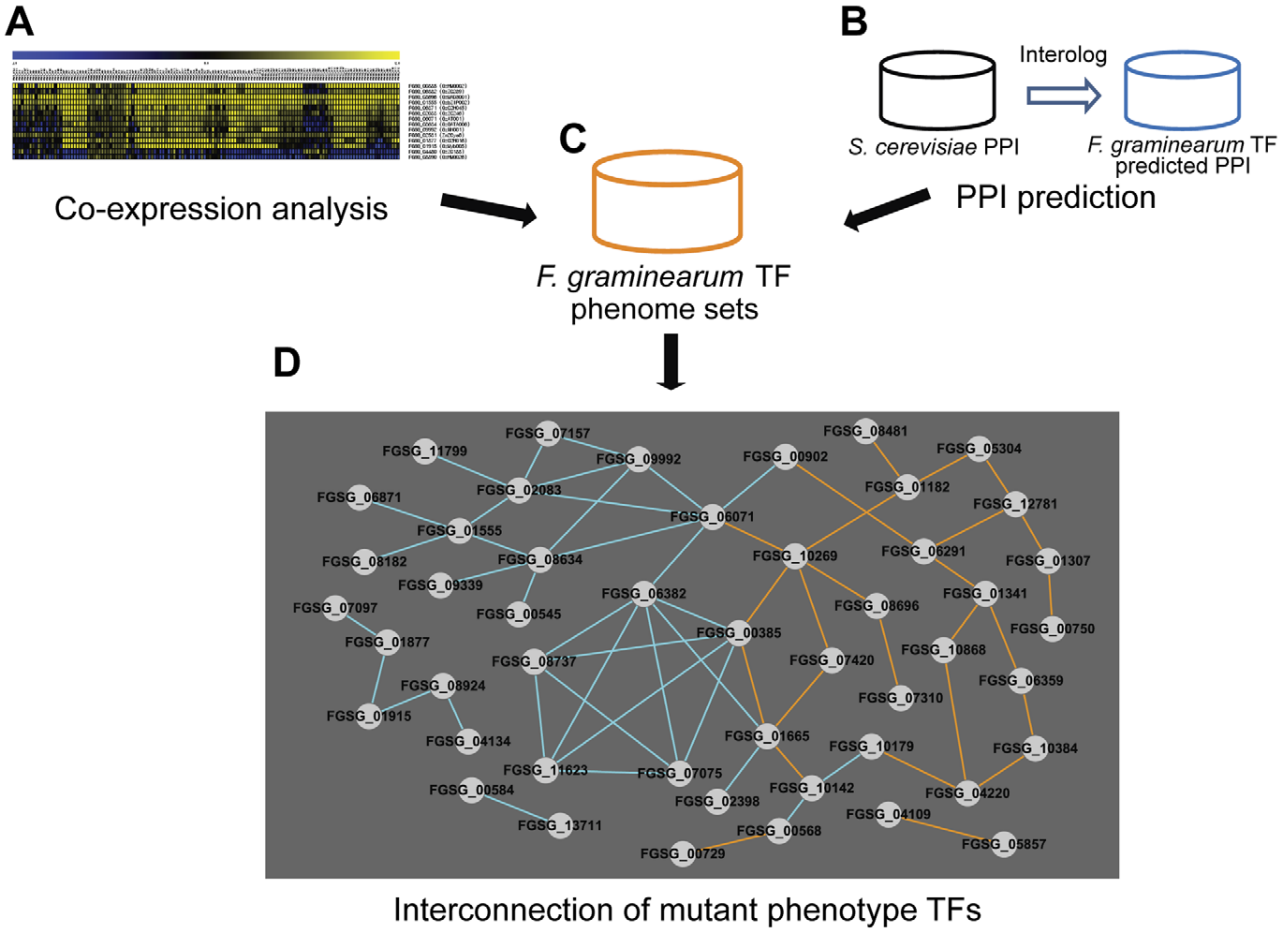
Transcription factors (TFs) with phenotype changes are interconnected either by co-expression or predicted protein-protein interaction (PPI). (A) Co-expression analysis (PCC>0.75 cut-off) of *Fusarium graminearum* TFs using public microarray data. PCC values were calculated using the 121 *F. graminearum* Affymetrix array data (Table S11). (B) Predicted PPI of *F. graminearum* TFs using the interolog map of the *S. cerevisiae* PPI dataset. (C) Phenome of *F. graminearum* TFs was generated. (D) Interconnection of mutant phenotype TFs through integration of co-expression (orange lines) and predicted PPI (blue lines). The nodes represent TFs whose knock-out displays a mutant phenotype.

Dissecting transcriptional regulatory networks (TRNs) and determining which TFs participate in individual cellular processes and how they interact are main goals in the field of TF research. Analyses of mutants showing mutant phenotypes provide valuable information for addressing these questions. We attempted to identify a TRN in *F. graminearum* via construction of TF connections through patterns of gene expression and predicted protein-protein interaction. In order to analyze TF gene expression, we built *F. graminearum* gene transcript co-expression networks for the putative TFs based on PCC, calculated using the publicly available Affymetrix microarray data (Table S11). The use of PCC was successfully applied previously to validate other biological data [27]. We defined correlated and anticorrelated interactions through values of PCC (PCC>|0.75|) that is corresponding to approximately 0.6% portion of all PCC pairs (Table S12). We identified 426 TF pairs that have PCC>|0.75| (Table S12 and Figure 5A). Next, we predicted protein-protein interaction (PPI) among TF pairs using interologs in *S. cerevisiae* from the PPI database. Using this approach, 243 PPI pairs were predicted for *F. graminearum* TFs (Table S13 and Figure 5B). These co-expression and predicted PPI data were incorporated into our phenome dataset containing 170 TFs whose deletion results in at least one mutant phenotype (Figure 5C). Deletion of 35 out of 426 co-expressed TF pairs and 25 out of 243 predicted PPI pairs resulted in a mutant phenotype (Figure 5D). These interconnected 48 TFs have higher BLAST scores (*p* = 2.23×10^−4^) than the remaining 122 TFs whose deletion results in a mutant phenotype. Thus, these interconnected TFs, which are members of various subfamilies, may coregulate diverse functions.

## Discussion

Taking advantage of genome sequences of several model organisms, genome-wide high-throughput genetic screens have been performed through the use of gene disruption methods [28], [29] or RNA interference [30], [31]. For fungi, the construction of genome-wide gene deletion sets for the budding yeast *S. cerevisiae* [28], [32] and the fission yeast *Schizosaccharomyces pombe* [33] serve as valuable resources. In phytopathogenic fungi, genome-wide functional analysis of rice blast fungus, *Magnaporthe oryzae*, using insertional mutagenesis mediated by *Agrobacterium* transformation [34] facilitated analysis of the genetic basis of fungal pathogenicity.

In this study, we generated a comprehensive phenome of *F. graminearum* 657 TFs in 17 traits and analyzed shared and unique phenotypes of individual TF mutants. In most case, deletion of the mutant phenotype TFs resulted in pleiotropic phenotype changes compared to the wild-type strain, whereas 46 mutants were defected in single phenotypic category (Table S5). Especially, specific genes functional for virulence (7 TFs) or conidiation (4 TFs) had rarely been found in *G. zeae*. Research on these 46 TFs will expand our understanding on fungal biology.

Integration of data from other genome-wide analyses, such as transcriptome analysis and predicted PPI, into the constructed phenome, as well as investigation of the distribution pattern of TFs across the fungal kingdom revealed that deletion of single TFs that are widely-distributed in fungal species exhibited a greater chance of causing phenotypic defects. This observation was also supported by phenotypic analysis of 31 TFs unique to *F. graminearum* (Table S10). The result is similar with a hypothesis that essential genes are more evolutionary conserved than nonessential genes in bacteria because negative selection acted on more stringent essential genes [35]. Conserved TFs among fungal species might have more important functions in survival, development, and adaption.

The interaction and coordination of multiple TFs provides the ability to regulate cellular processes through fine-tuned transcriptional control. A recent study reported that high correlation between two phenotypes is highly predictive by known indicators of biological data such as protein-interactions [36]. Among the 170 TFs whose deletion resulted in mutant phenotypes, the 26 TFs whose connection was predicted using PPI are widely distributed among fungi and have higher gene expression levels compared to the other 144 TFs. Thus, these 26 TFs could be essential for the control of development and virulence in *F. graminearum*, as well as other fungi, and will be good resources to study the *F. graminearum* transcriptional network.

Our phenotypic data are mostly consistent with previously reported TF studies except the phenotype of *Tri6* deletion mutant [37]. The previous report demonstrated that DON-nonproducing mutants showed reduced virulence on wheat head [37]. However, phenotype network in this study showed weak correlation (*p*-value = 3.25×10^−12^ and PCC = 0.5162) between virulence and DON production (cut-off with *p*-value<10^−12^ and PCC>0.6, Table S6 and Figure 2D). This discrepancy may be due to differences in experimental conditions and wheat cultivars. Differences in virulence between wheat plants inoculated with the wild-type strain and a DON-nonproducing mutant were more dramatic in the field tests than in controlled environment studies [37], [38]. In our study, we used a growth chamber for virulence test where the condition highly favors for disease development. In addition, the cultivar Eunpamil used for our study has been known to be highly susceptible to *Fusarium* head blight. Further virulence test in different field condition is necessary to resolve this discrepancy.

We revealed that there is no correlation between the stresses except between osmotic and ROS stress. The correlation between osmotic and ROS stress may be due to associated changes in membrane physiology. Further analysis of diverse stress responses in the 657 mutants generated in this study could provide a novel control strategy for *F. graminearum*.

Comparison of phenotypes between *F. graminearum* and *N. crassa* TF orthologs suggested that these TFs evolved divergently in how they control growth, asexual, and sexual development, rather than keeping the same function. These divergences may have been caused by differences in the life cycles between the two fungi. *N. crassa* is an obligate saprophyte that obtains its nutrients from dead or decaying matter, and *F. graminearum* is a facultative saprophytic plant pathogen that additionally needs specialized functions in order to obtain nutrients from plants. In addition, although both fungi might have similar features during sexual reproduction, i.e. repeat-induced point mutation, *F. graminearum* completes sexual reproduction homothallically, while *N. crassa* is heterothallic and the homothallic nature of *F. graminearum* may require unique pathways to complete sexual reproduction. The availability of a genome-wide TF deletion mutant library for *F. graminearum*, may provide a valuable resource for investigating sexual development and a reference for studying how different fungi have evolved to control various cellular processes at the transcriptional level.

## Materials and Methods

### Manual identification of 16 putative TFs

These TFs were identified based on the presence of specific motif associated with TFs or sequence homology with known TFs as listed in Table S1.

### Fungal strains and media

*G. zeae* strain GZ3639 [39] was used for generating deletion mutants. All strains were stored as conidial suspensions in 20% glycerol at −70°C and deposited to the Center for Fungal Genetics Resources (CFGR, http://genebank.riceblast.snu.ac.kr/).Media were prepared and used according to the *Fusarium* laboratory manual [2].

### DNA extraction, Southern blot, and PCR

Genomic DNA was extracted as previously described [2]. Restriction endonuclease digestion and Southern blot were performed following standard techniques [40]. PCR primers used in this study (Table S14) were synthesized by Bionics (Seoul, Korea).

### Targeted gene deletion

Mutant alleles for targeted gene deletion were generated using the double-joint (DJ) PCR method [41]. A geneticin resistance gene cassette (*gen*) and the 5′ and 3′ flanking region of each TF gene were amplified from pII99 and GZ3639, respectively, and fused by DJ PCR using the conditions previously described [42]. Finally, split marker recombination was performed using primer sets from Table S14 [43]. At least three mutants were isolated for each gene deletion via co-dominant PCR screening. To exclude wild-type strain and ectopic mutants, (TF name)-5F/(TF name)-with 5F primers set was used. (TF name)-5F and Gen-with 5F primer set was used for positive selection of deletion mutants. In order to confirm a single copy integration of the mutant allele, Southern hybridization was performed (Figure S1).

### Asexual development, stress test, and virulence test

In most cases, three independent mutants for each gene deletion were used for phenotyping with two replications. Radial growth on potato dextrose agar (PDA), minimal media (MM), and carrot agar were measured at three and five days after inoculation with freshly grown culture plugs from MM. The percentage of the average radial growth of mutants compared to the wild-type strain was calculated. Pigmentation of the bottom of PDA plates representing aurofusarin accumulation after culturing for five days was compared to wild-type strain and scored intensity of red color (0–6) with naked eyes.

Conidia production was measured by counting the number of conidia after culturing culture plugs from MM in 5 ml of carboxymethylcellulose medium (CMC) [44] for five days at 25°C on a rotary shaker (150 rpm). The virulence of fungal strains was determined on the susceptible wheat cultivar Eunpamil as previously described [24].

Various stress conditions were established in which the wild-type strain showed about one half of the radial growth observed on CM medium without any stress inducing agent: oxidative stress (5 mM hydrogen peroxide and 0.1 mM menadione) [45], osmotic stress (1 M NaCl, 1 M KCl, 1.5 M sorbitol, and 6 mM FeSO_4_), pH stress (pH = 4 and pH = 11), cell wall stress (60 mg/L Congo Red and 5 mg/L sodium dodecyl sulfate), DNA synthesis inhibition (8.6 mg/L fungicide iprodione), inhibition of mitogen-activated protein kinase pathway (0.023 mg/L fungicide fludioxonil). Radial growth of mutants on CM amended with each stress-inducing agent was evaluated compared to growth on CM.

### Sexual development and mycotoxin analysis

Mycelia grown on carrot agar for five days were rubbed with a glass spreader after applying 2.5% sterilized Tween-60 solution to induce sexual reproduction [2]. After this treatment, all cultures were incubated under UV light (365 nm; HKiv Import & Export Co., Ltd., Xiamen, China) at 25°C. Perithecium growth was scored. Maturation of perithecia was determined by measuring their size. The presence of ascospores, their morphology, and ascospore discharge were also checked. In order to determine the effect of TF deletion on mycotoxin production, fungal strains were grown on 50 g of rice substrate for three weeks at 25°C in the dark. Rice cultures were harvested, and mycotoxins were extracted as previously described [46]. A portion of each extract was analyzed using TLC to quantify toxin production.

### Construction of the co-expression network

We calculated Pearson correlation coefficient (PCC) scores to measure the correlation between expression of individual TF genes based on two sets of publicly available Affymetrix and NimbleGen microarray data. The raw Affymetrix data was downloaded from PLEXdb (http://www.plexdb.org/). We analyzed the raw Affymetrix data using MAS 5.0 R-package [47]. The trimmed mean target intensity of each array was arbitrarily set to 500, and the data were then log_2_ transformed. The mapping of Affymetrix probe sets onto *F. graminearum* genes was performed using the *Fusarium graminearum* Genome Database (FGDB).

### TF conservation analysis

We utilized the BLASTMatrix tool that is available on the Comparative Fungal Genomics Platform (CFGP, http://cfgp.riceblast.snu.ac.kr/) for this analysis [26]. In brief, ungapped BLASTP with BLOSUM62 alignment scoring matrix was used without any filtering and BLAST scores calculated by “score = −log (expect)” equation were downloaded from the BLASTMatrix analysis results.

### Prediction of *F. graminearum* TF protein-protein interactions (PPI)

Yeast interactome datasets were obtained from DIP (release 2010-6-14), BIOGRID (version 3.0.67), IntAct (release 2010-7-30), MINT (release 2010-07-27) and SGD (downloaded on 2010-8-2). The number of nonredundant PPIs is 88,720. Orthologs from yeast and *F. graminearum* were identified using Inparanoid version 4.1 [48], [49].

### Microscopic observation

Microscopic observation was performed with a DE/Axio Imager A1 microscope (Carl Zeiss, Oberkochen, Germany).

## Supporting Information

Figure S1 **Deletion confirmation of deletion mutants by Southern blot.** Restriction enzymes used for each blot and the size of DNA standards (kb) are indicated on the right of each blot. WT, *G. zeae* wild-type strain GZ3639; H, *Hind*III; E1, *EcoR*I, E5, *EcoR*V; B, *Bgl*II; S, *Sal*I; Sc, *Sac*I; Nd, *Nde*I; Nr, *Nru*I; B1, *BamH*I; Xh, *Xho*I; Xb, *Xba*I; P1, *Pst*I; C1, *Cla*I; SS1, *Ssp*I; K1, *Kpn*I.
(PDF)

Figure S2 **Mycelia growth of** ***G. zeae*** **strains on potato dextrose agar (PDA).** Fungal strains were grown on PDA for five days. WT, *G. zeae* wild-type strain GZ3639.
(PDF)

Figure S3 **Mycelia growth of** ***G. zeae*** **strains on minimal media (MM).** Fungal strains were grown on MM for five days. WT, *G. zeae* wild-type strain GZ3639.
(PDF)

Figure S4 **Sexual development of seven groups of TF mutants.** Each strain was inoculated on carrot agar. The photographs were taken 10 days after sexual induction. WT, *G. zeae* wild-type strain GZ3639. Scale bar = 0.5 mm (dissecting microscope images) and 100 µm (differential interference contrast images).
(PDF)

Figure S5 **Virulence of TF mutants on wheat heads.** A center spikelet of each wheat head was injected with 10 µl of conidia suspension. The photographs were taken 14 days after inoculation. WT, *G. zeae* wild-type strain GZ3639.
(PDF)

Figure S6 **Toxin production using thin layer chromatography (TLC) analysis.** Fungal strains were grown on rice substrate for three weeks. ZEA and DON are indicated in TLC plate image of the wild-type strain. WT, *G. zeae* wild-type strain GZ3639. ZEA, zearalenone; DON, deoxynivalenol.
(PDF)

Figure S7 **Phenotype of TF mutants under various stress conditions.** The photographs were taken three days after inoculation. WT, *G. zeae* wild-type strain GZ3639. CM, mock complete medium; NaCl, CM with 1 M NaCl; KCl, CM with 1 M KCl; Sorbitol, CM with 1.5 M sorbitol; FeSO_4_, CM with 6 mM FeSO_4_; H_2_O_2_, CM with 5 mM; Mena, CM with 0.1 mM menadione; Fludi, CM with 0.023 mg/L fludioxonil; Ipro, CM with 8.6 mg/L iprodione; SDS, sodium dodecyl sulfate (SDS) 5 mg/L; C.R., Congo Red 60 mg/L; pH 4 and pH 11, CM with pH = 4 and pH = 11, respectively.
(PDF)

Figure S8 **Gene expression patterns of transcription factors, exhibiting defective sexual phenotypes in a deletion mutant, from perithecia development-related microarray data.** 105 defective sexual phenotypes were grouped (Table S8 and Figure 3) and two microarray experiments were employed to monitor expression patterns; one is a sexual development microarray on *G. zeae* wild-type strain PH-1 (from 0 h to 144 h) [5] and the other is a microarray on ascospore discharge of PH-1 mutant [6].
(PDF)

Table S1 **Manually selected putative TFs.**
(XLS)

Table S2 **Phenotypes from 709 transcription factors.**
(XLS)

Table S3 **Conidia production of** ***G. zeae*** **strains on carboxymethylcellulose medium (CMC).**
(XLS)

Table S4 **Summary of phenotypes from each TF family.**
(XLS)

Table S5 **TFs with single mutant phenotype.**
(XLS)

Table S6 **Analysis of phenotypic correlations via PCC.**
(XLS)

Table S7 **Comparison of phenotypes between two fungal mutants.**
(XLS)

Table S8 **Sexual phenotype grouping.**
(XLS)

Table S9 **The quantification of distribution of each TFs.**
(XLS)

Table S10 **Relationships between phenotypes, total blast scores, and gene expression in 657 TFs.**
(XLS)

Table S11 **Average of normalized intensity of each TF.**
(XLS)

Table S12 **Co-expression analysis of TFs.**
(XLS)

Table S13 **Predicted protein-protein interaction (PPI).**
(XLS)

Table S14 **Primers used for construction of TF mutants.**
(XLS)
