# Supplementary figures and images for: A Phenome-Based Functional Analysis of Transcription Factors in the Cereal Head Blight Fungus, Fusarium graminearum
Source: PLoS Pathog. 2011 Oct 20;7(10):e1002310. doi: 10.1371/journal.ppat.1002310 (PMC3197617; doi:10.1371/journal.ppat.1002310)

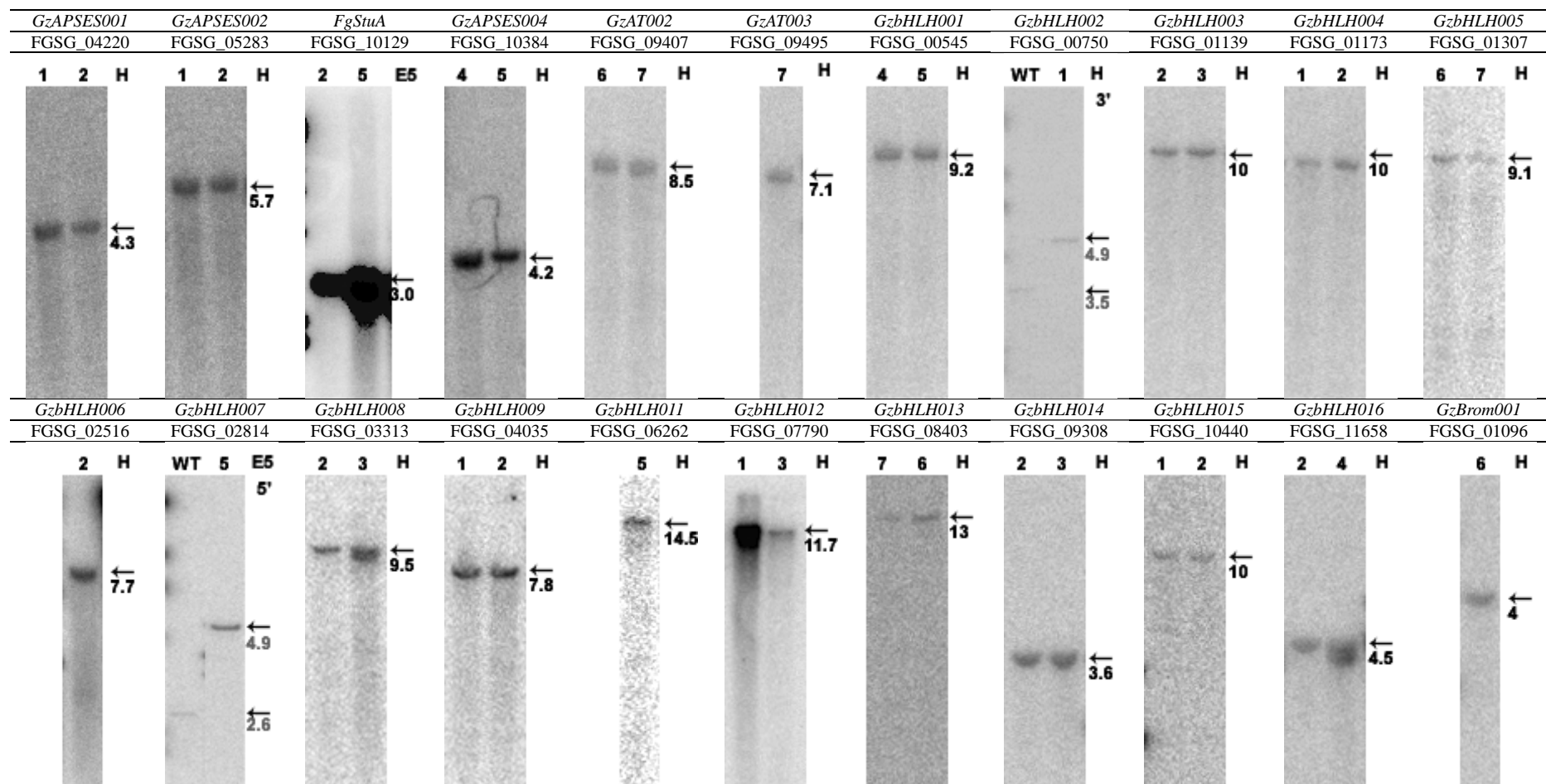

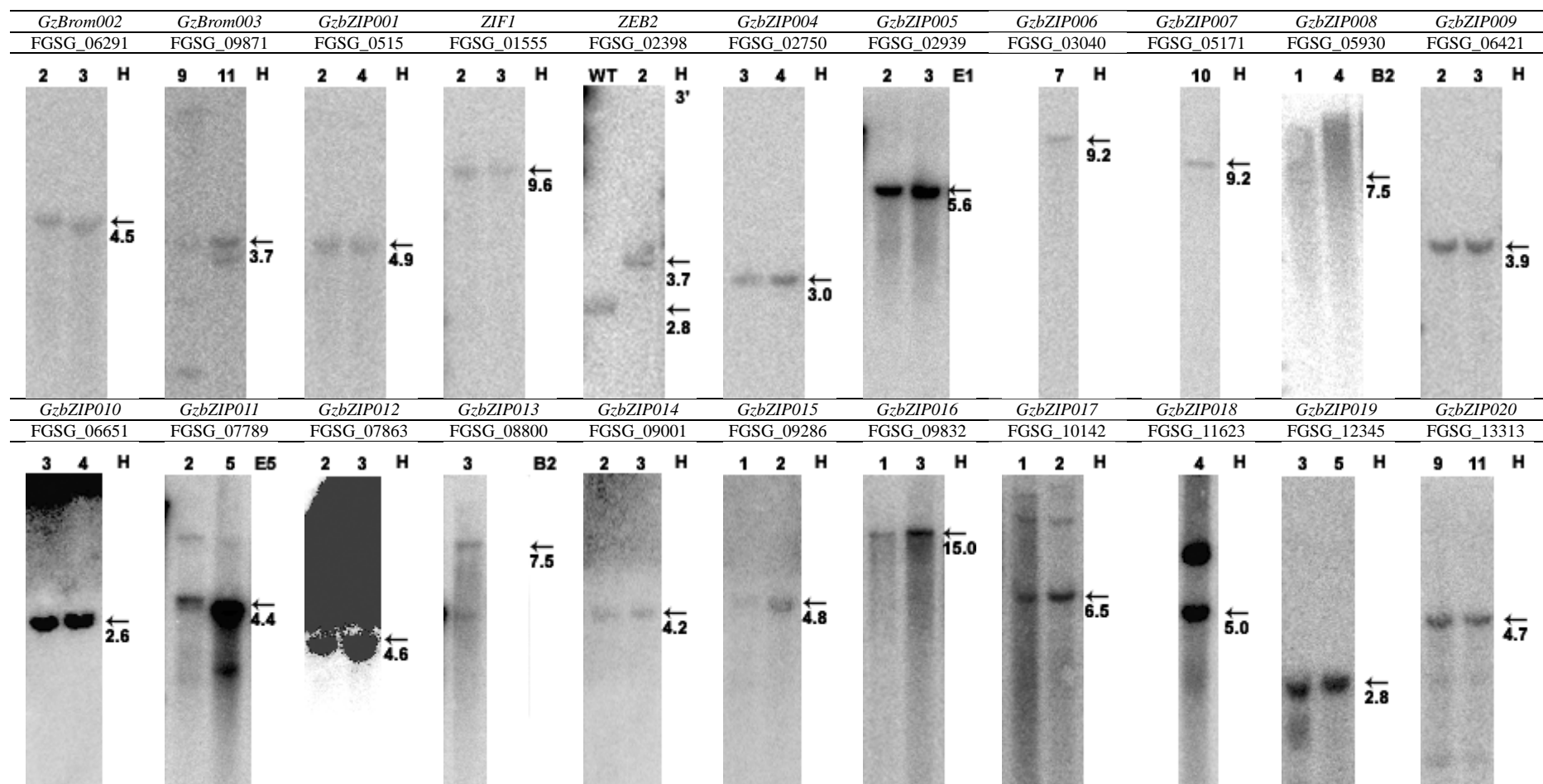

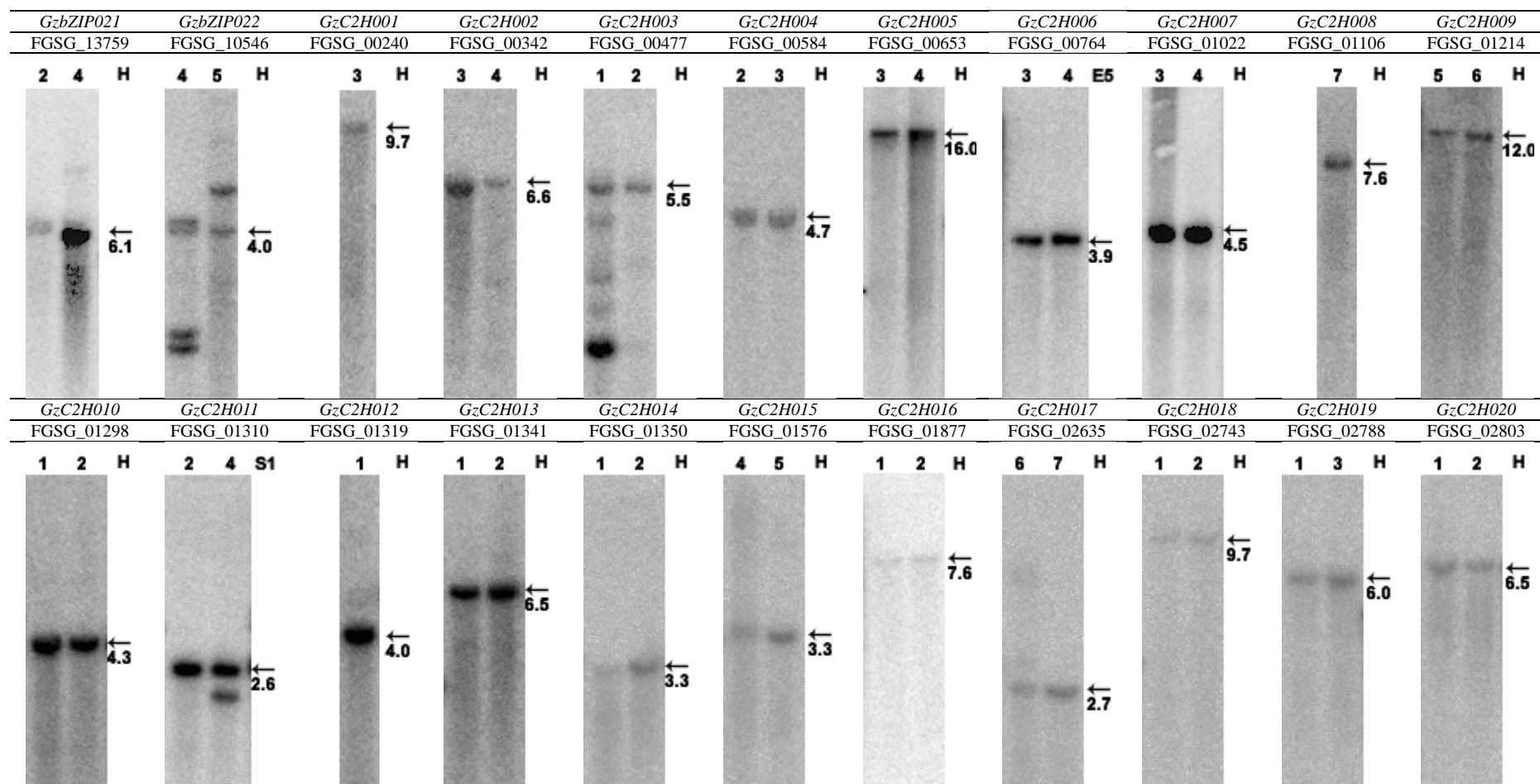

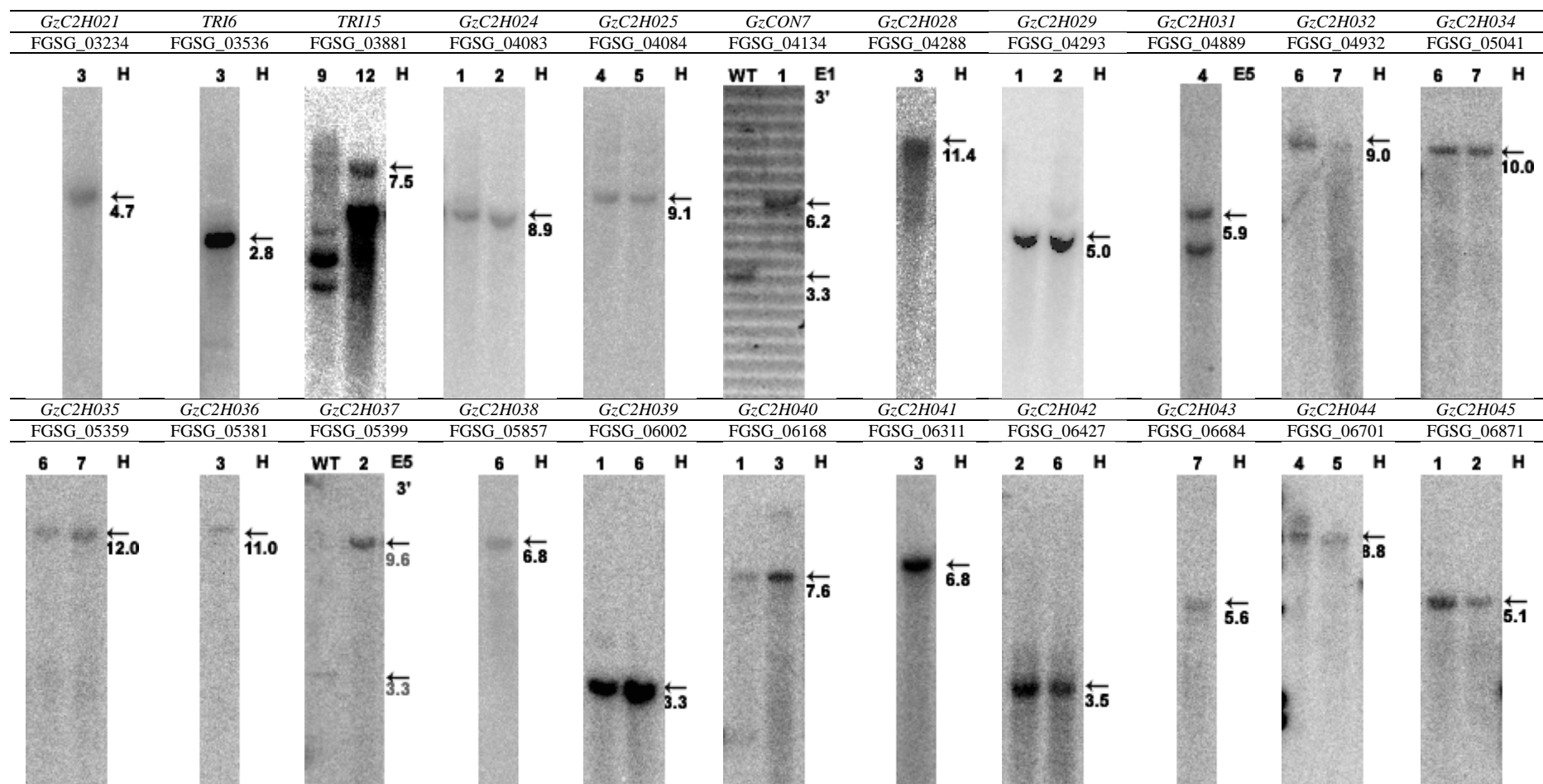

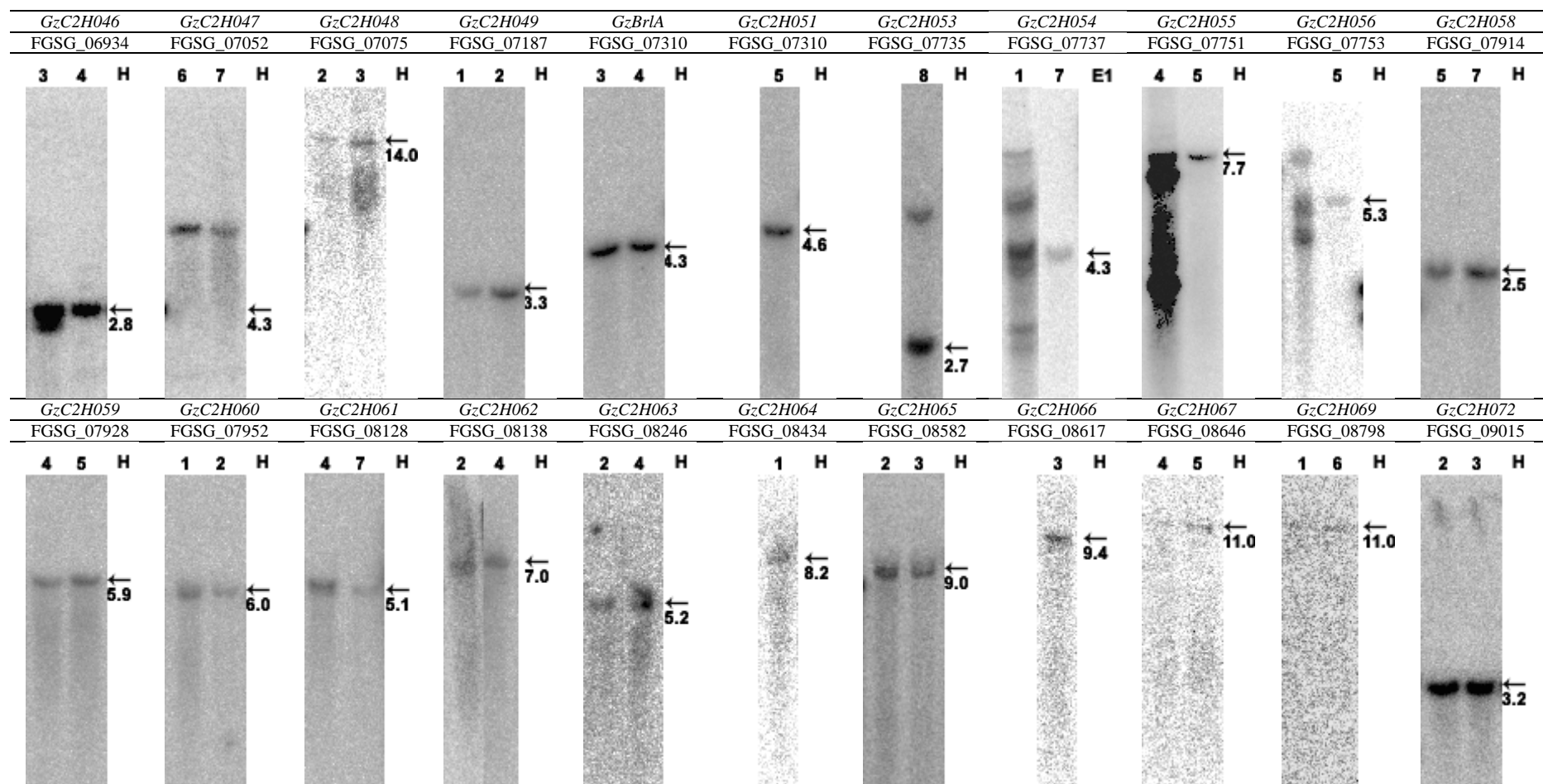

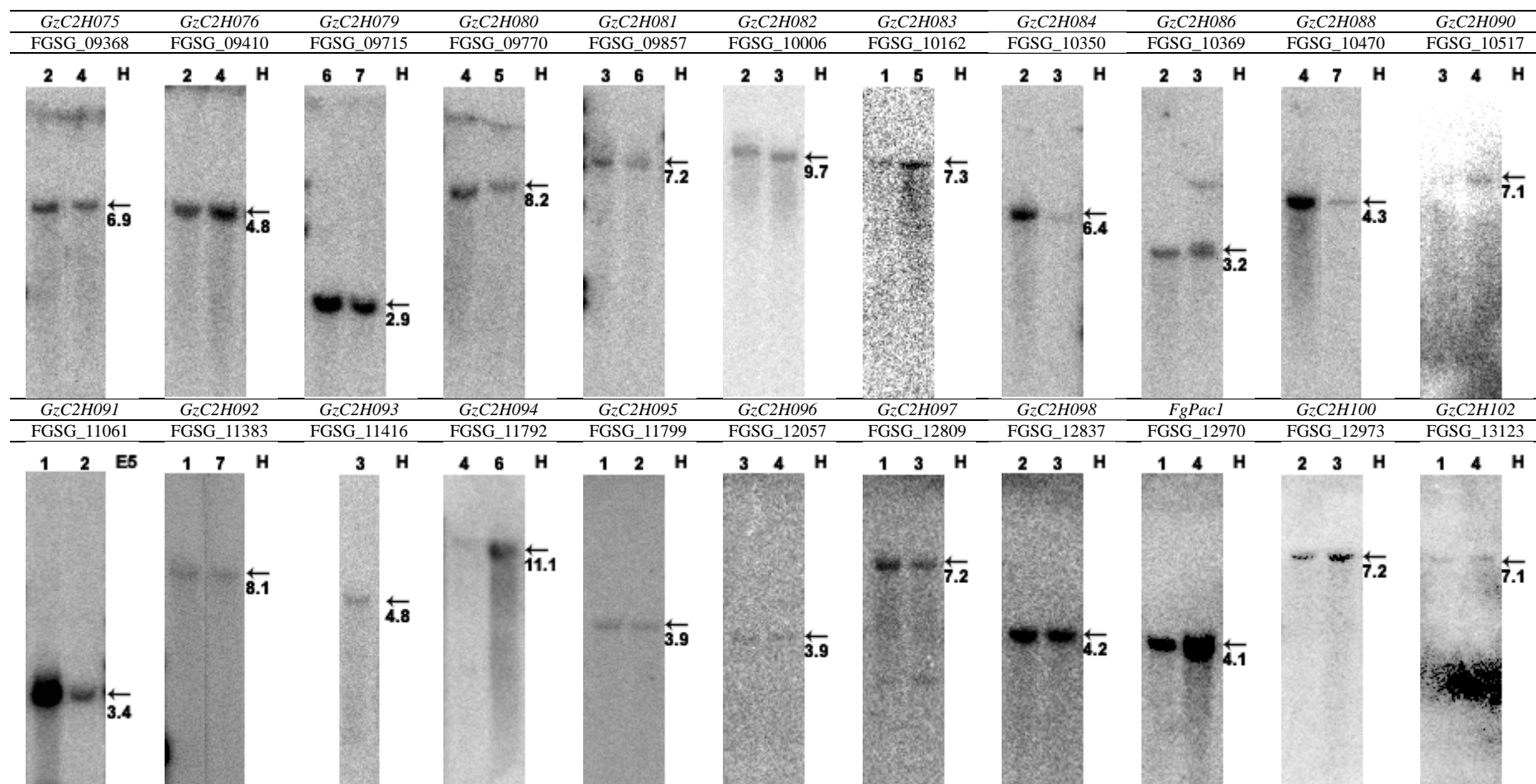

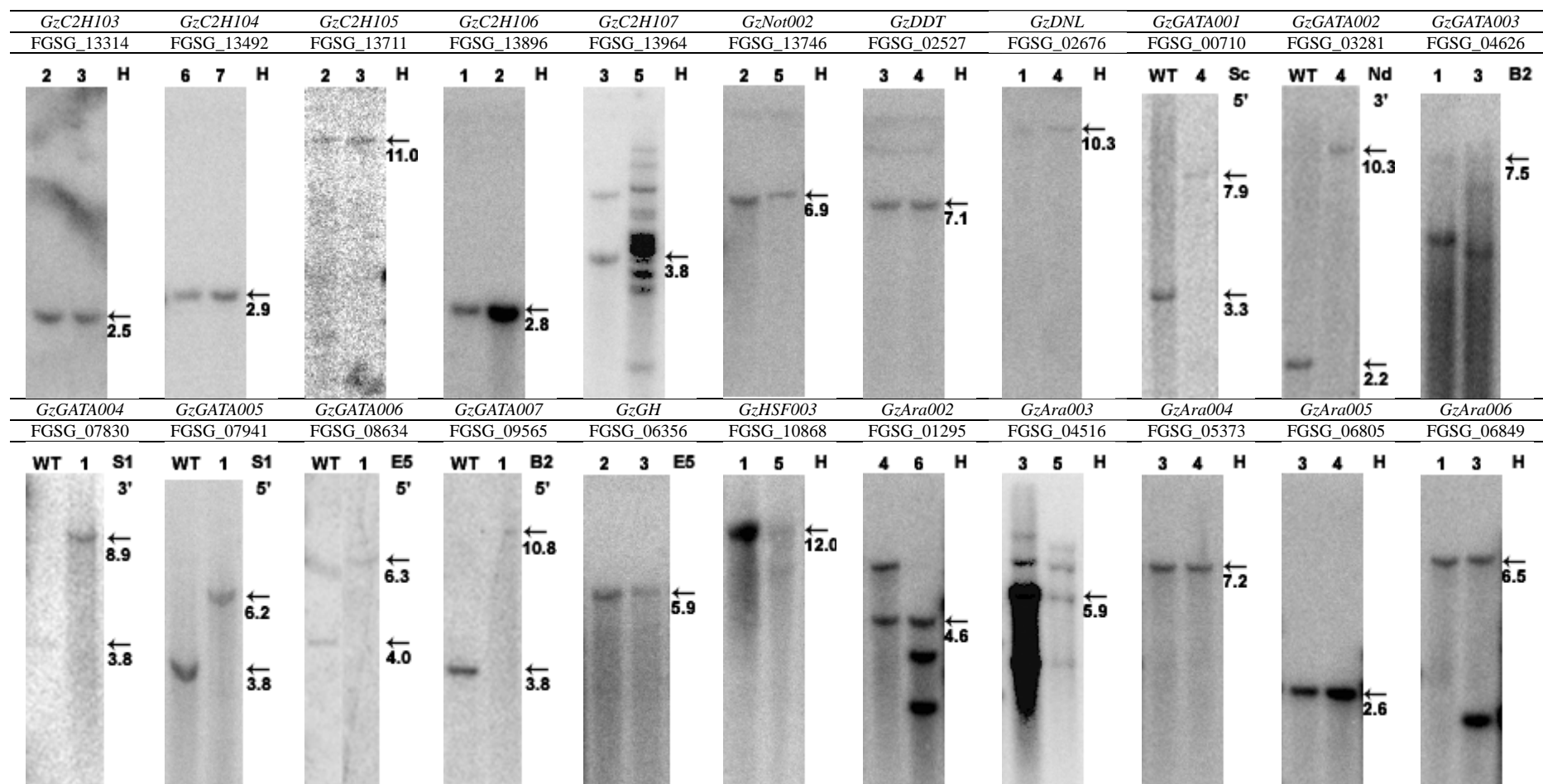

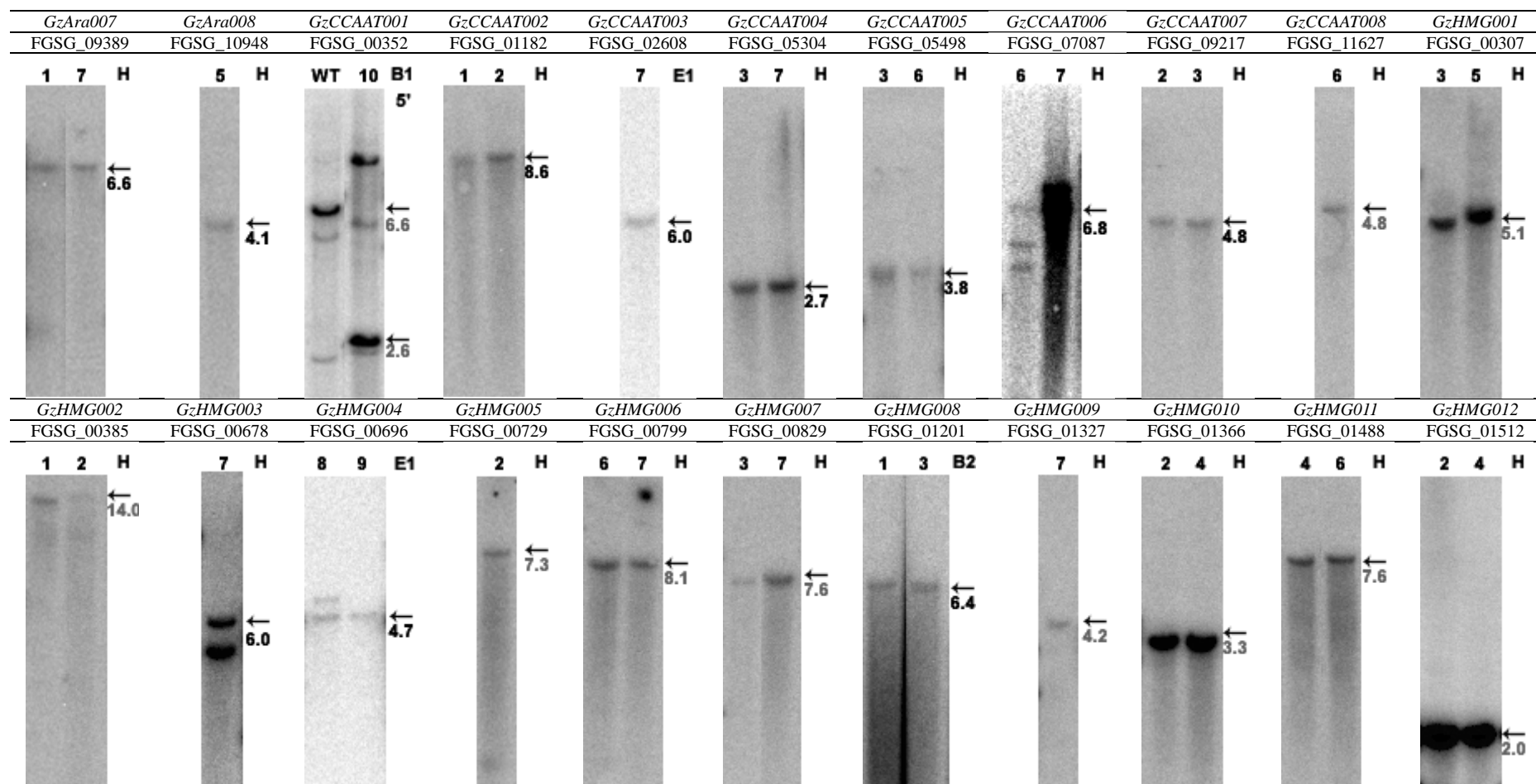

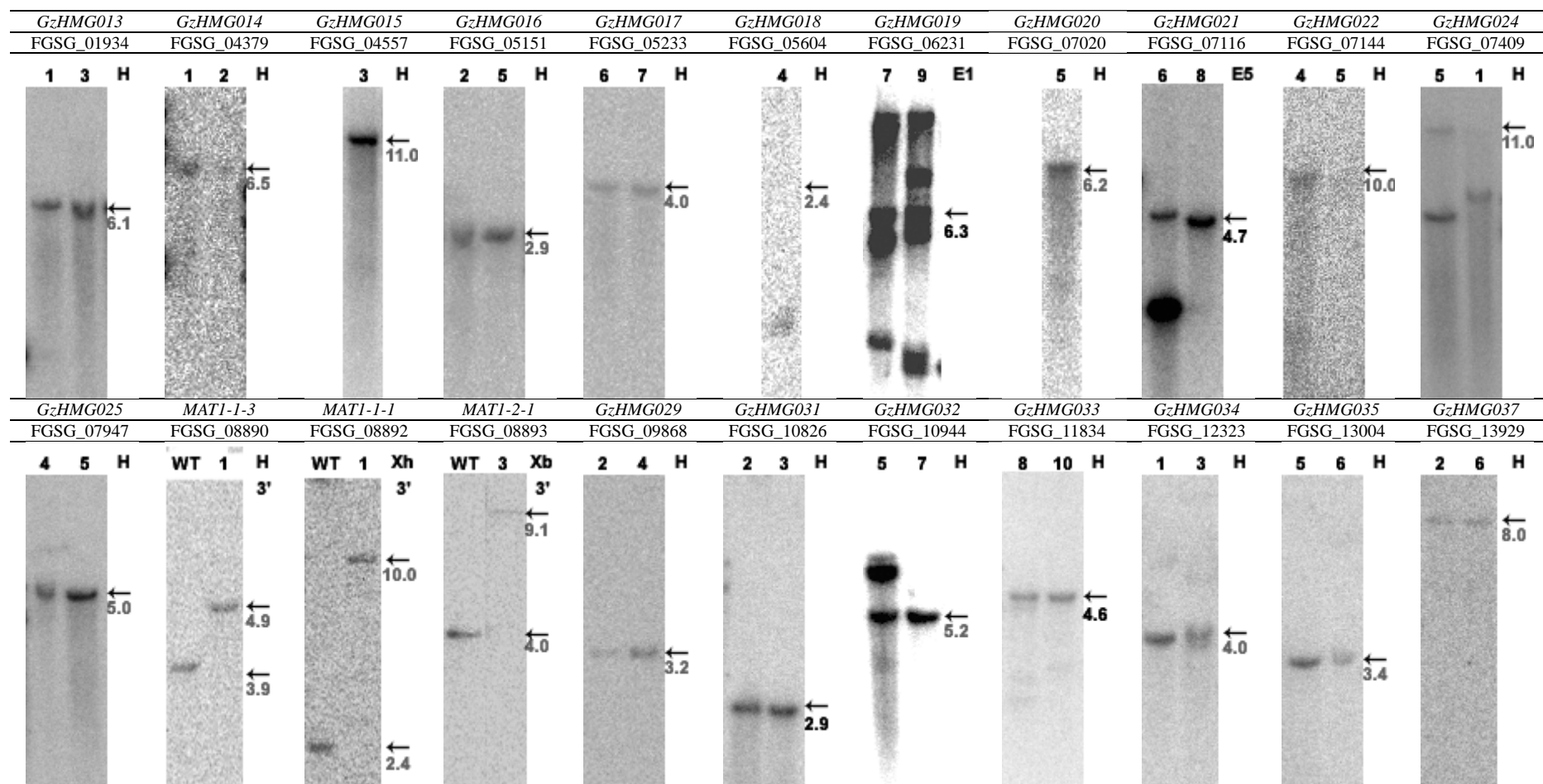

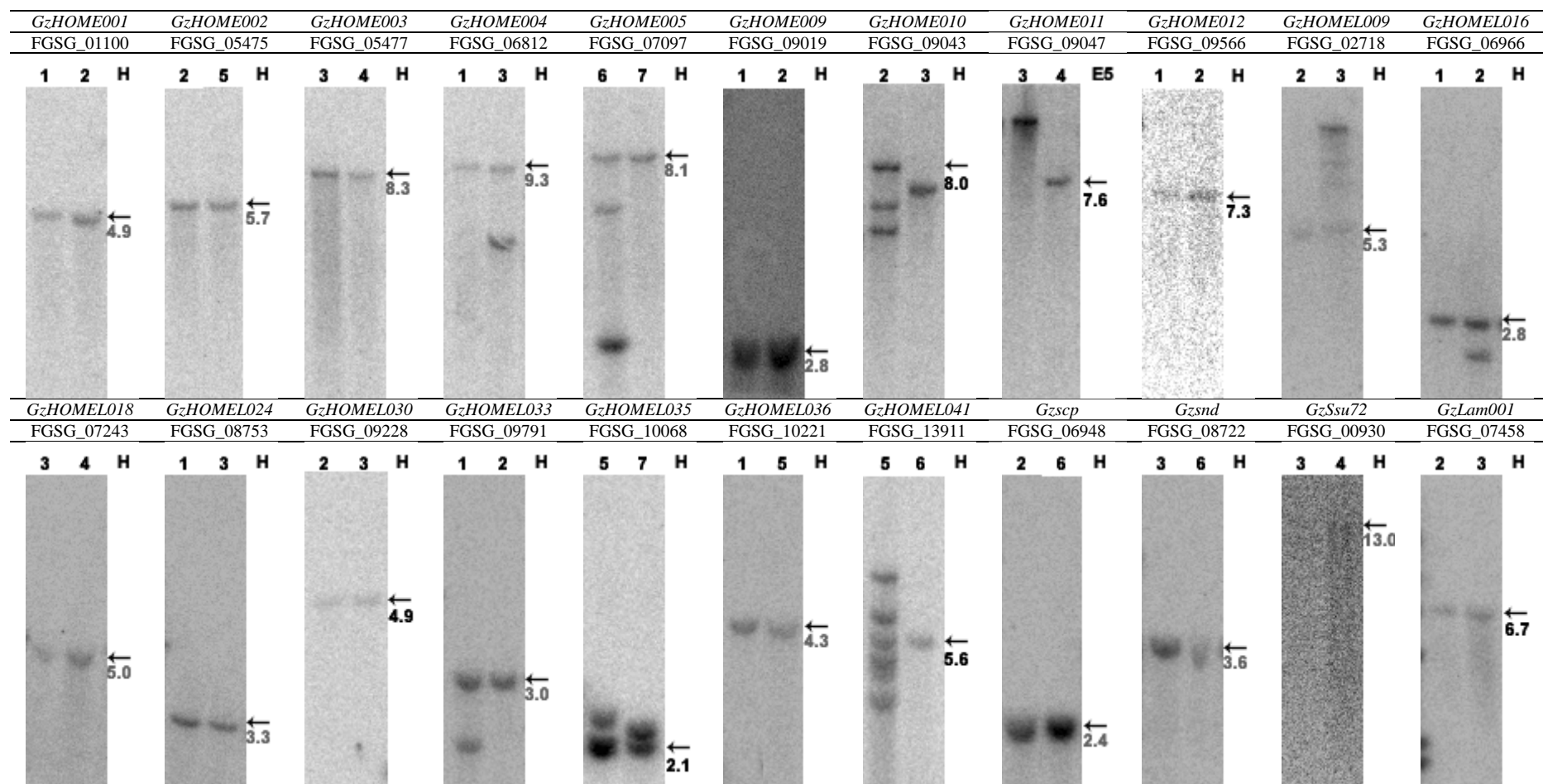

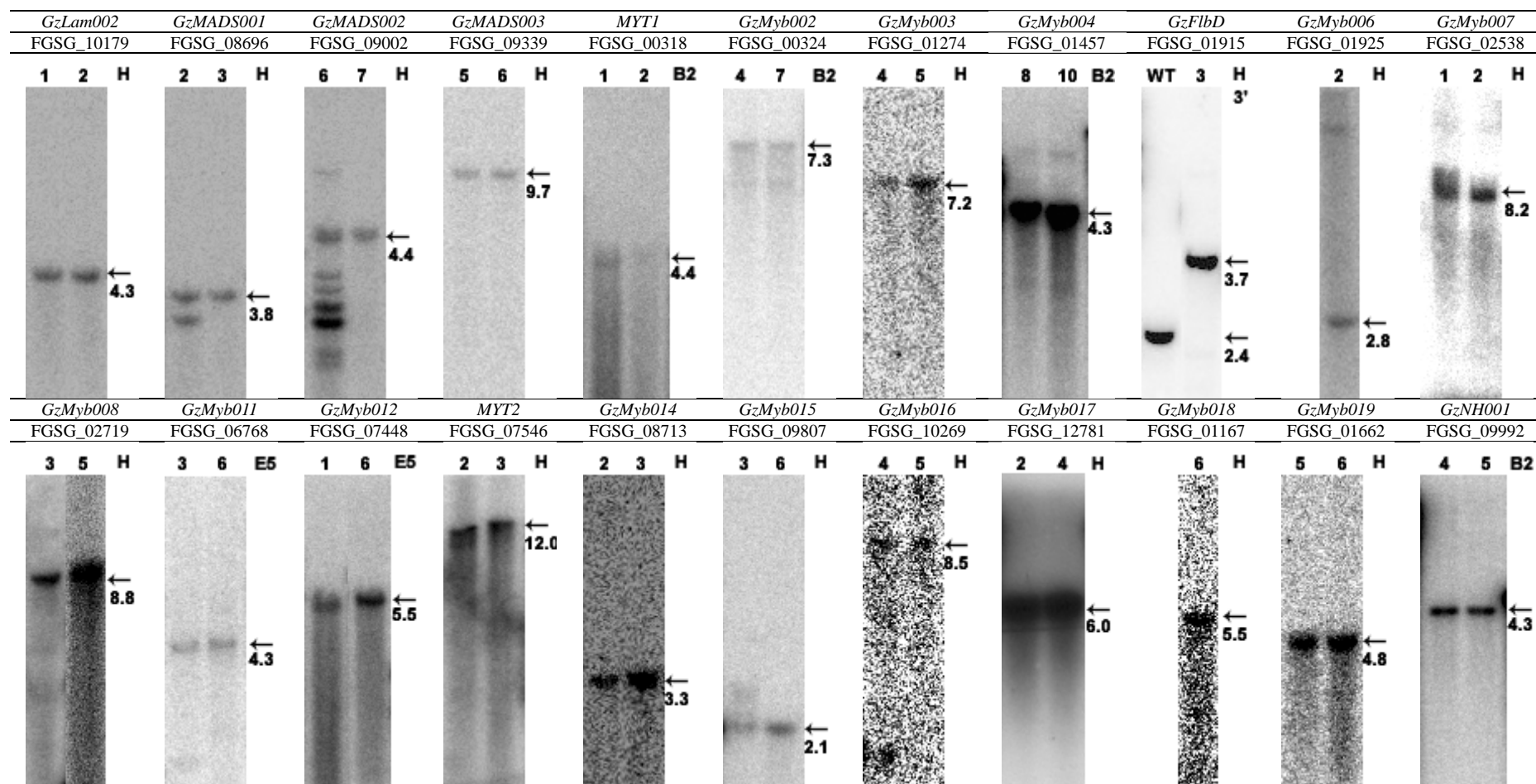

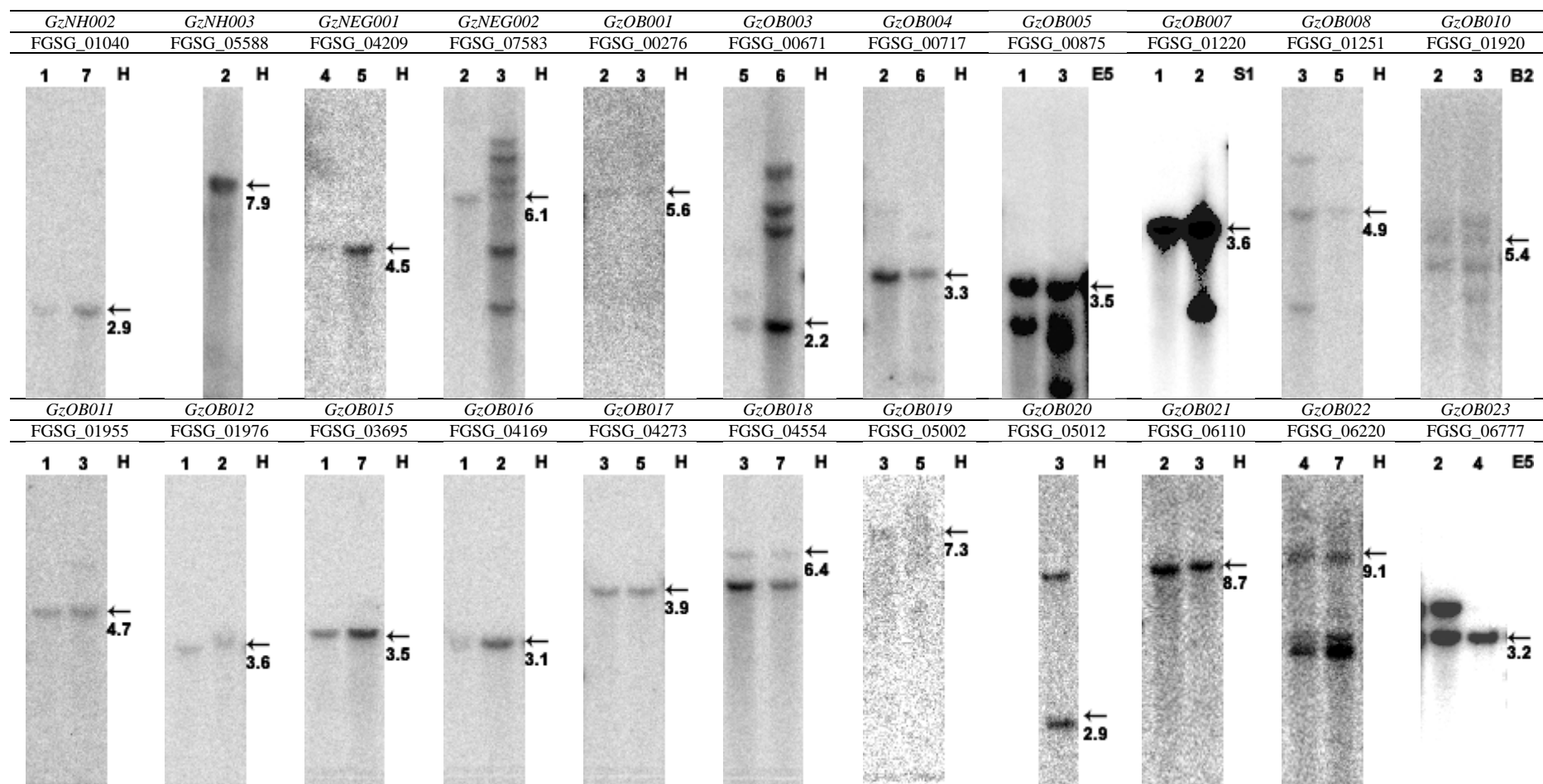

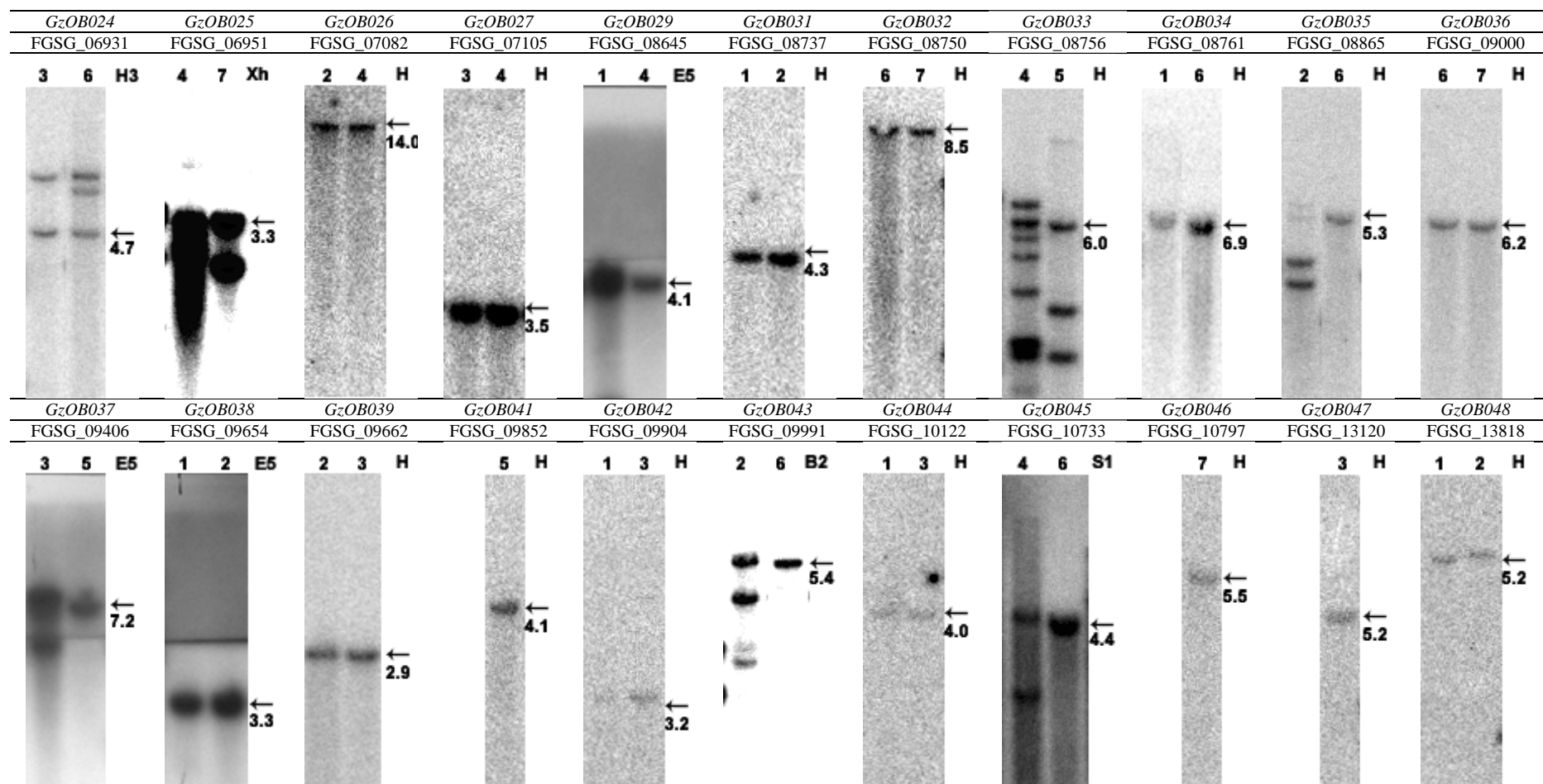

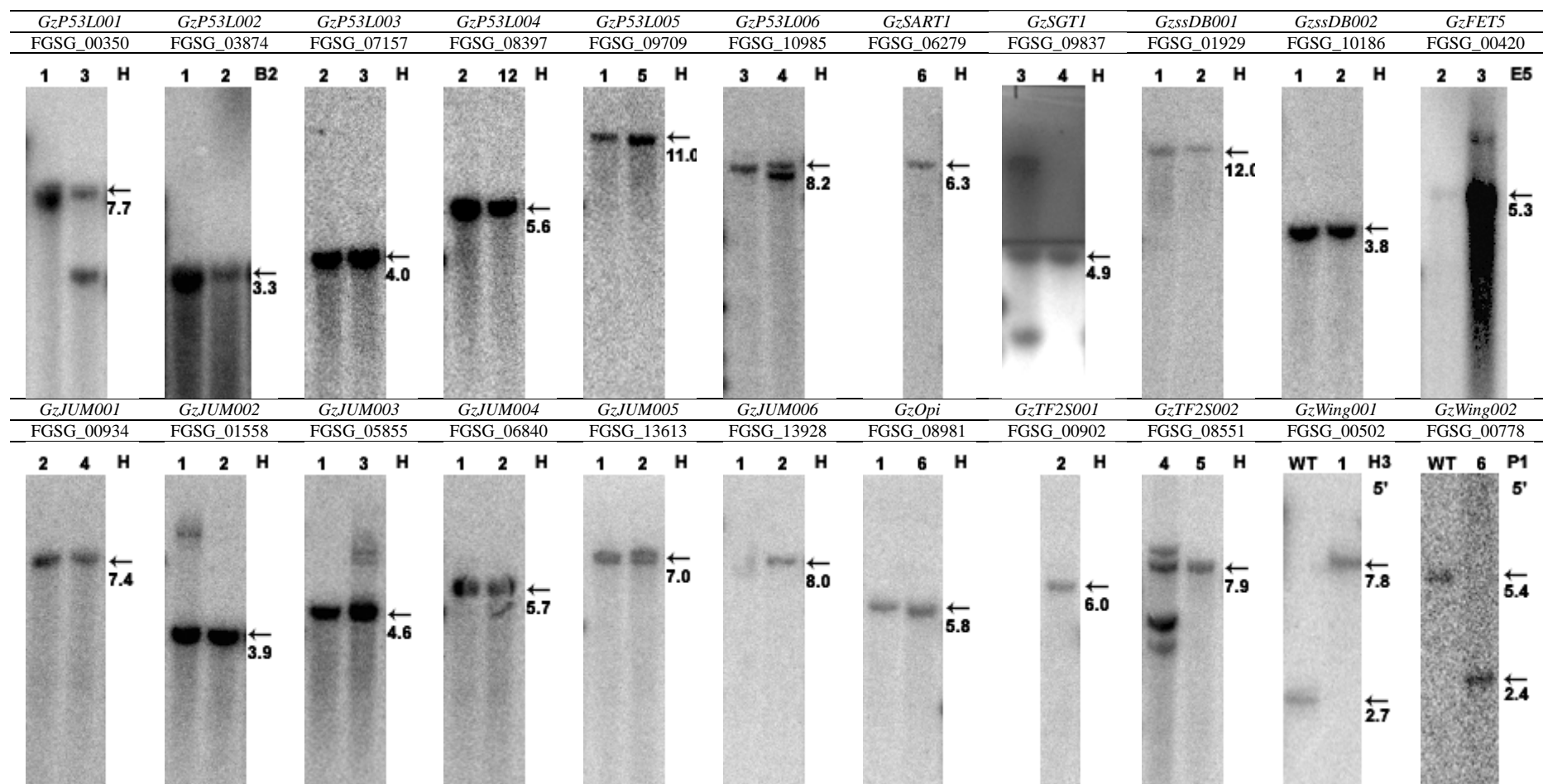

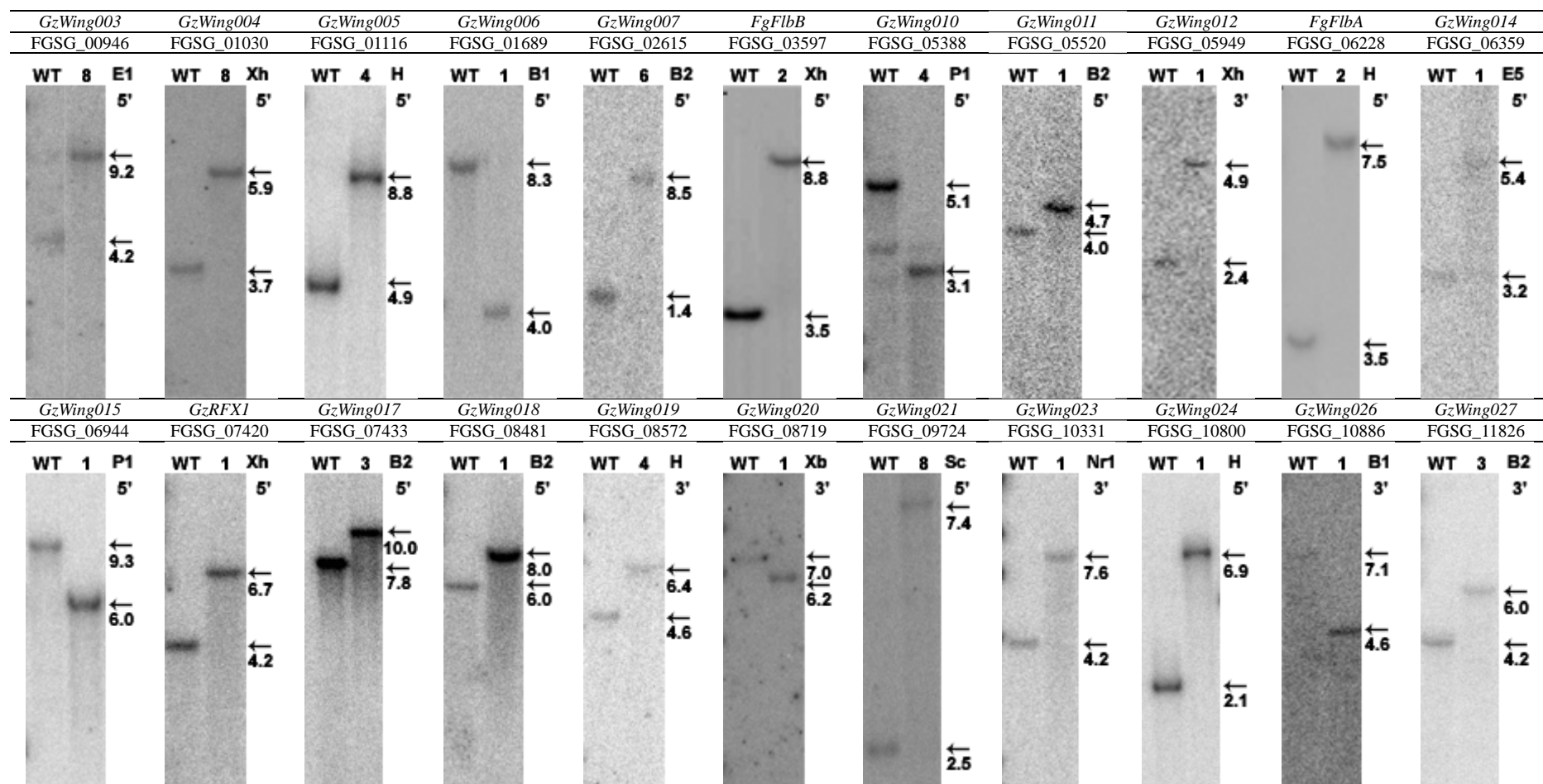

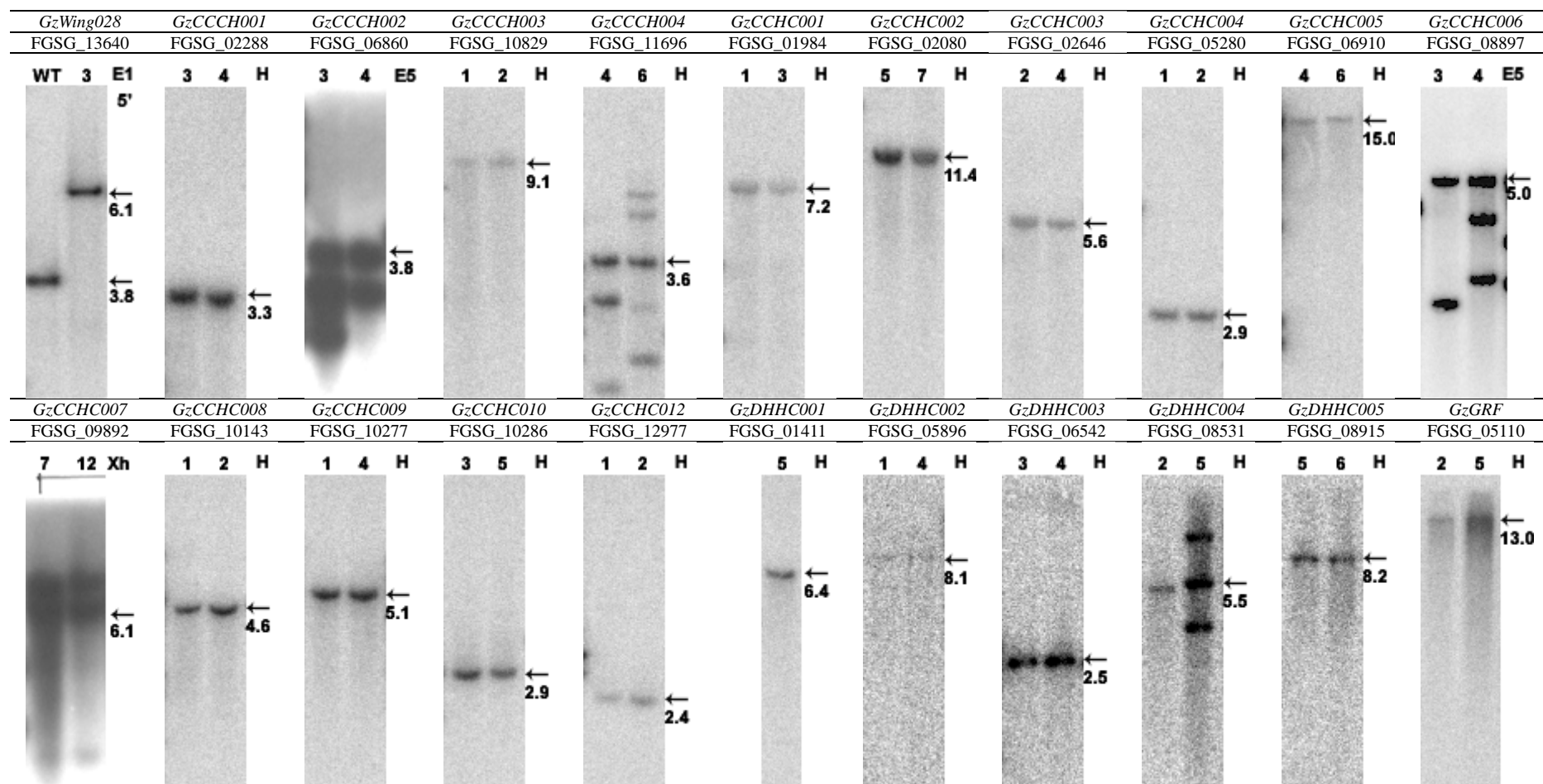

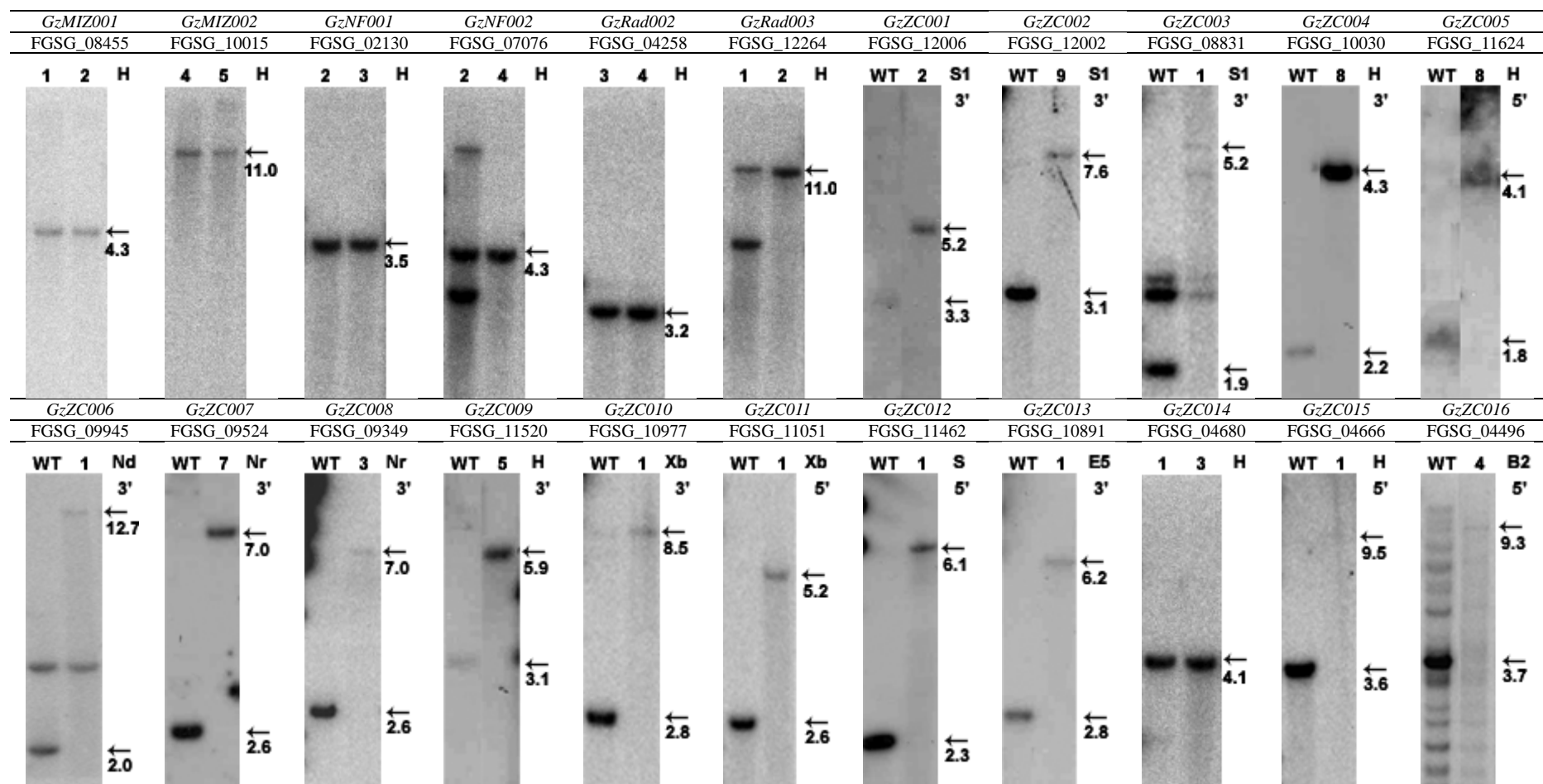

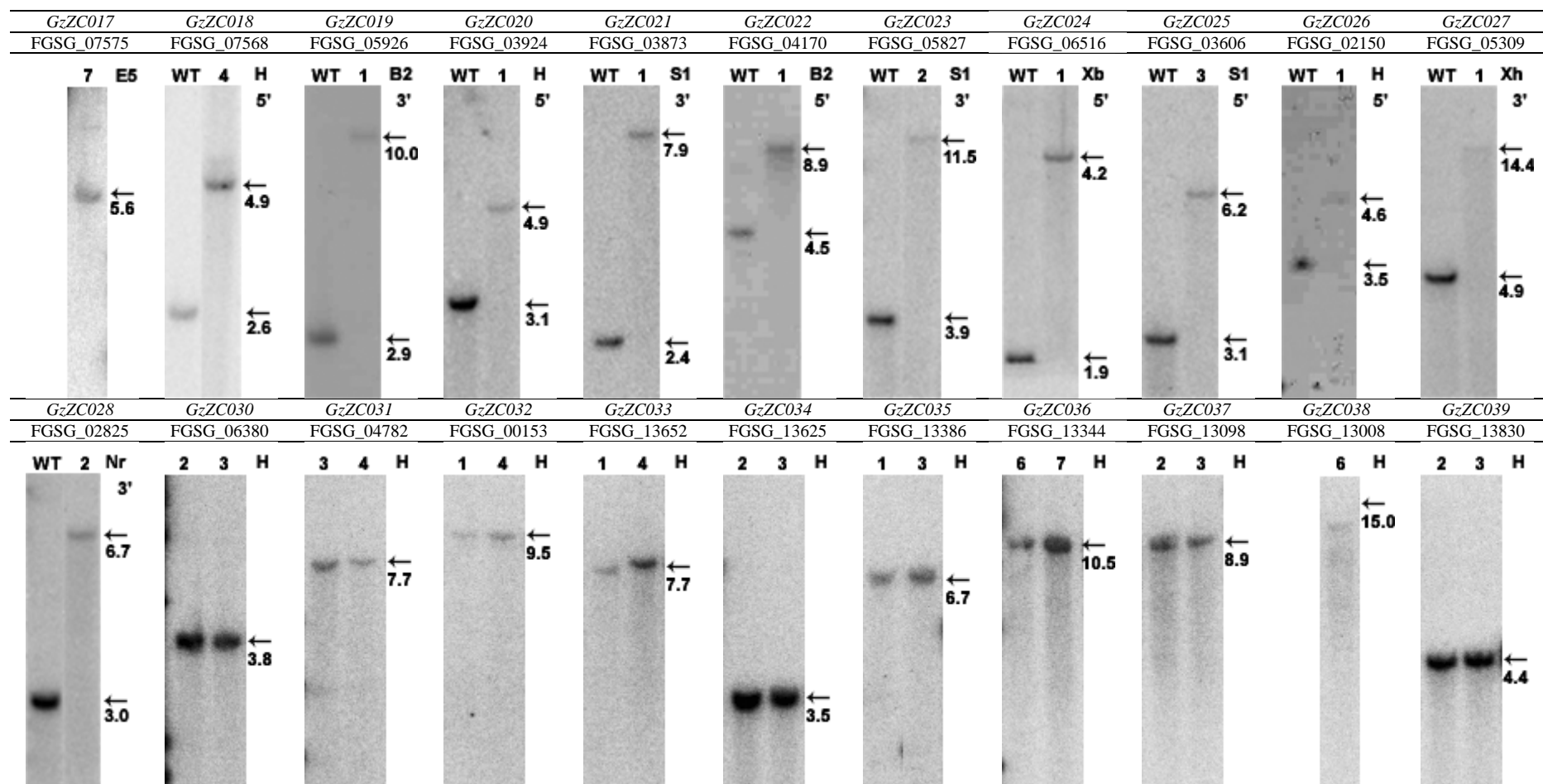

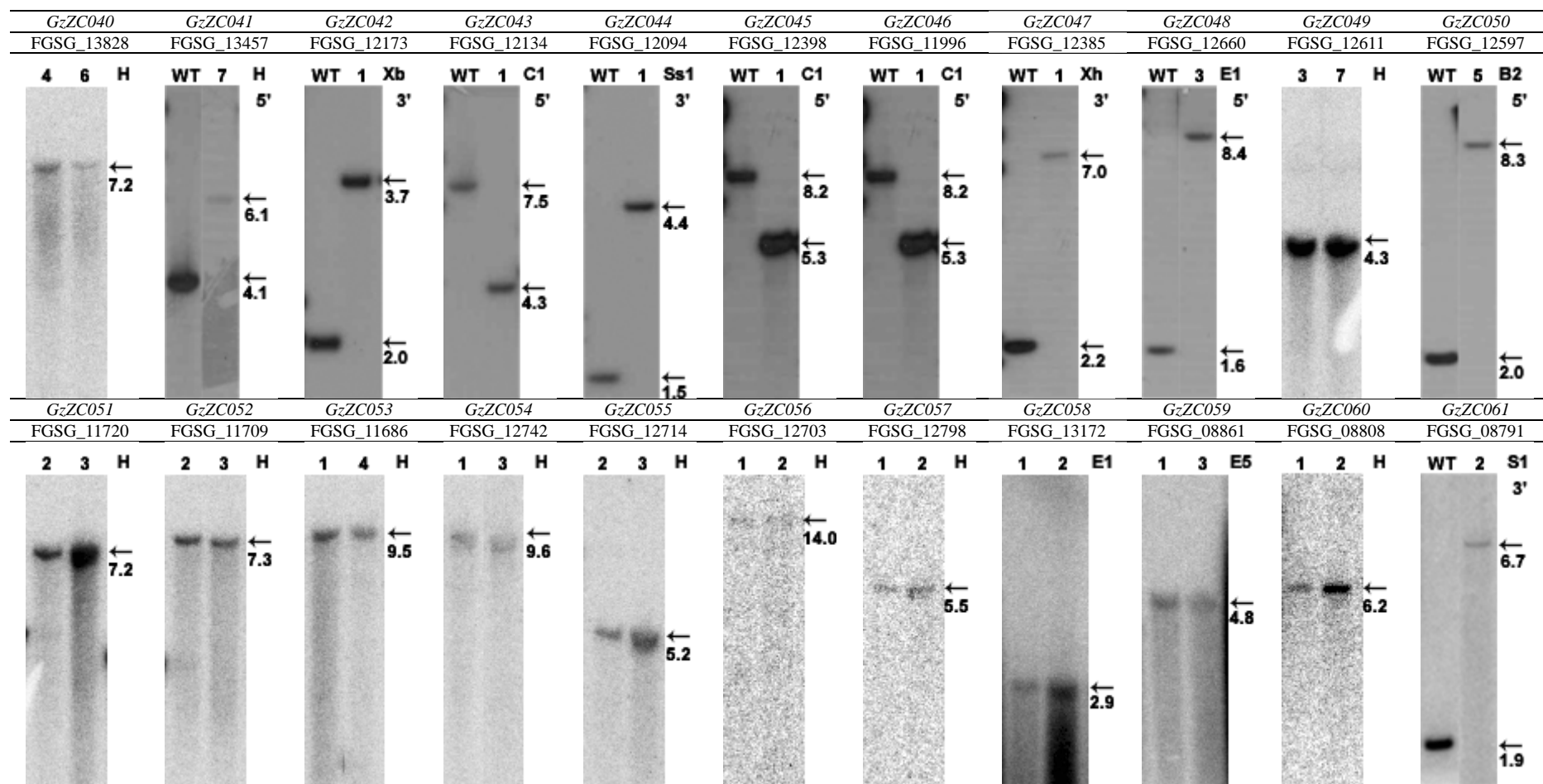

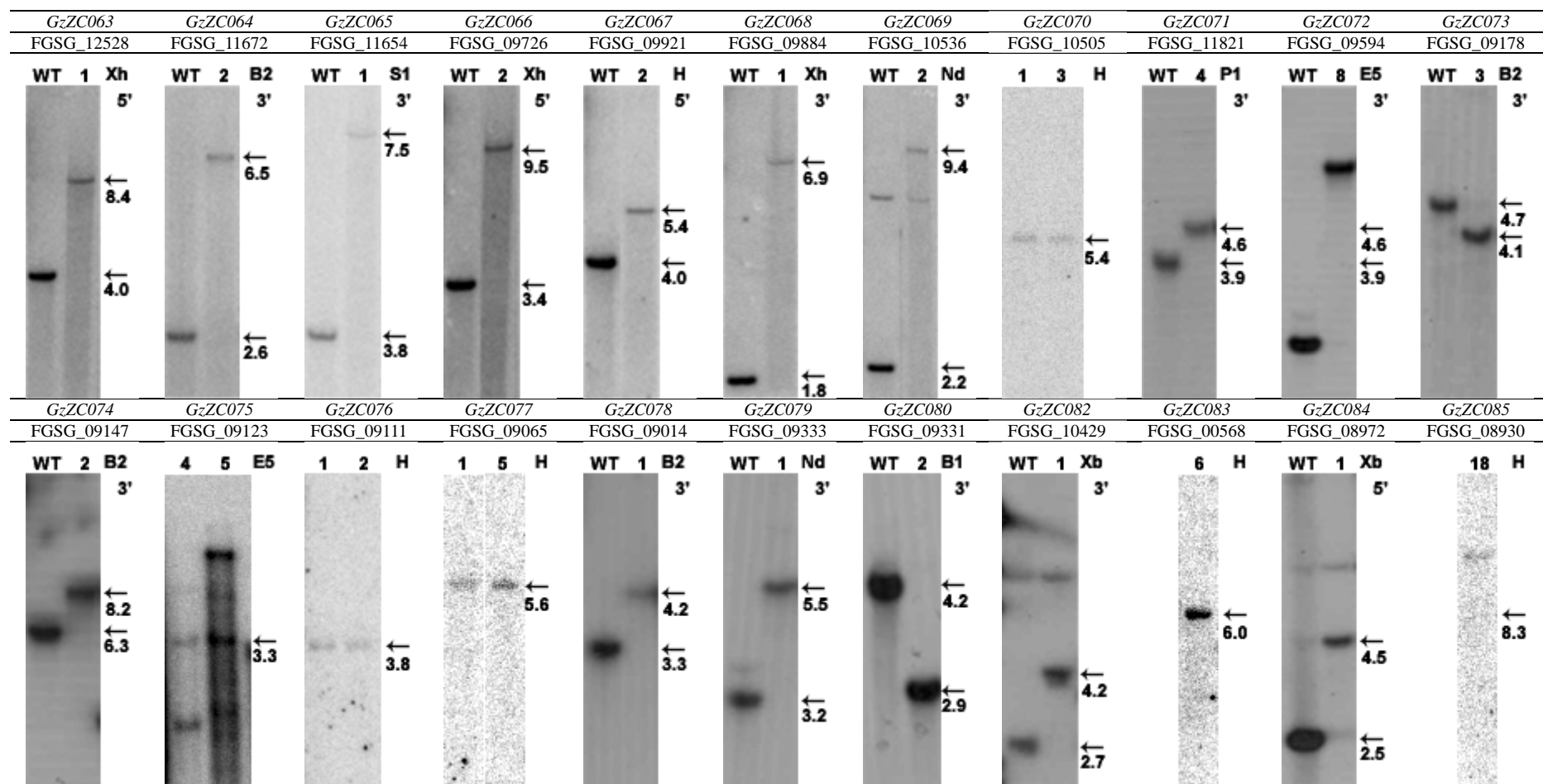

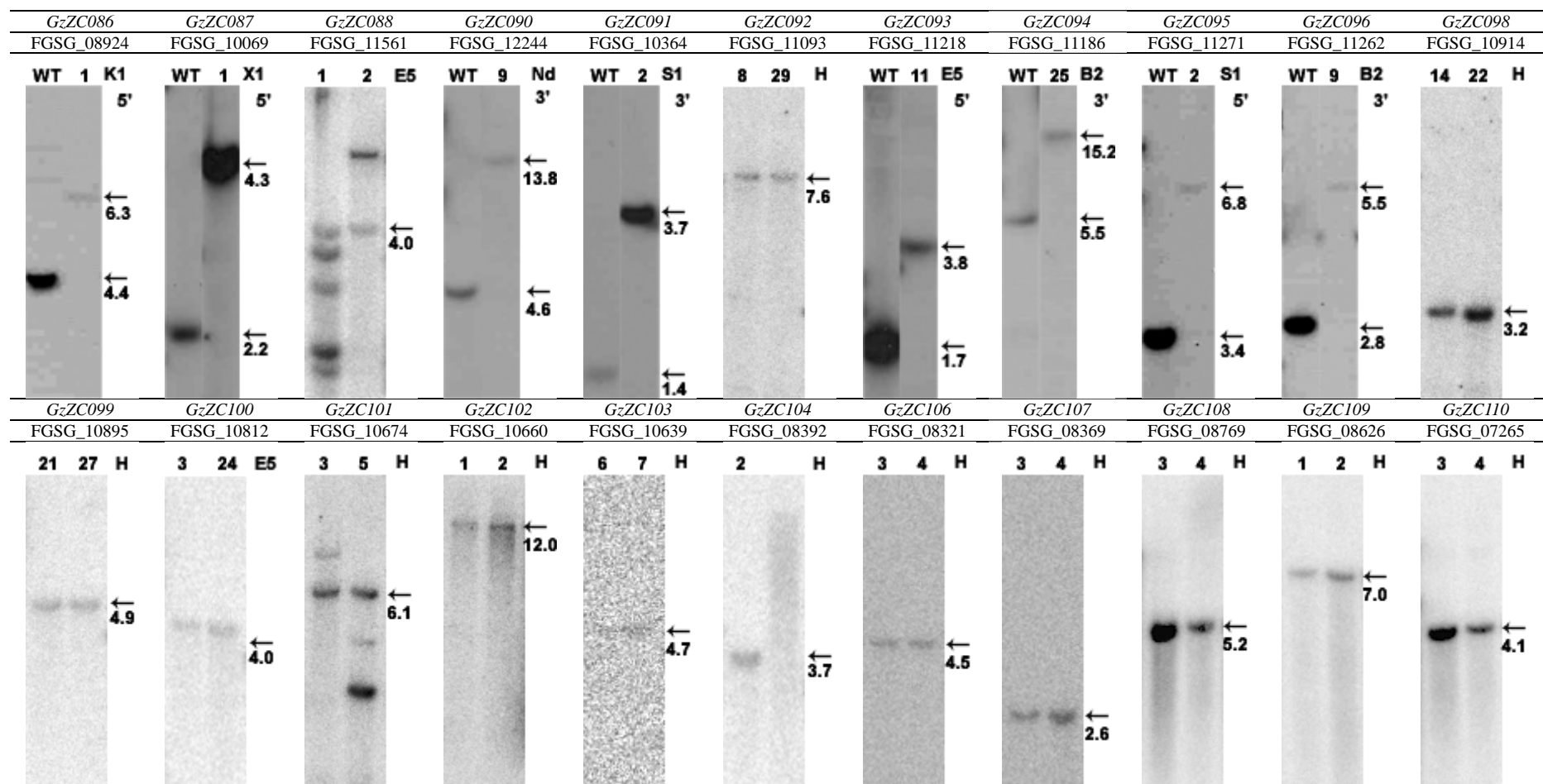

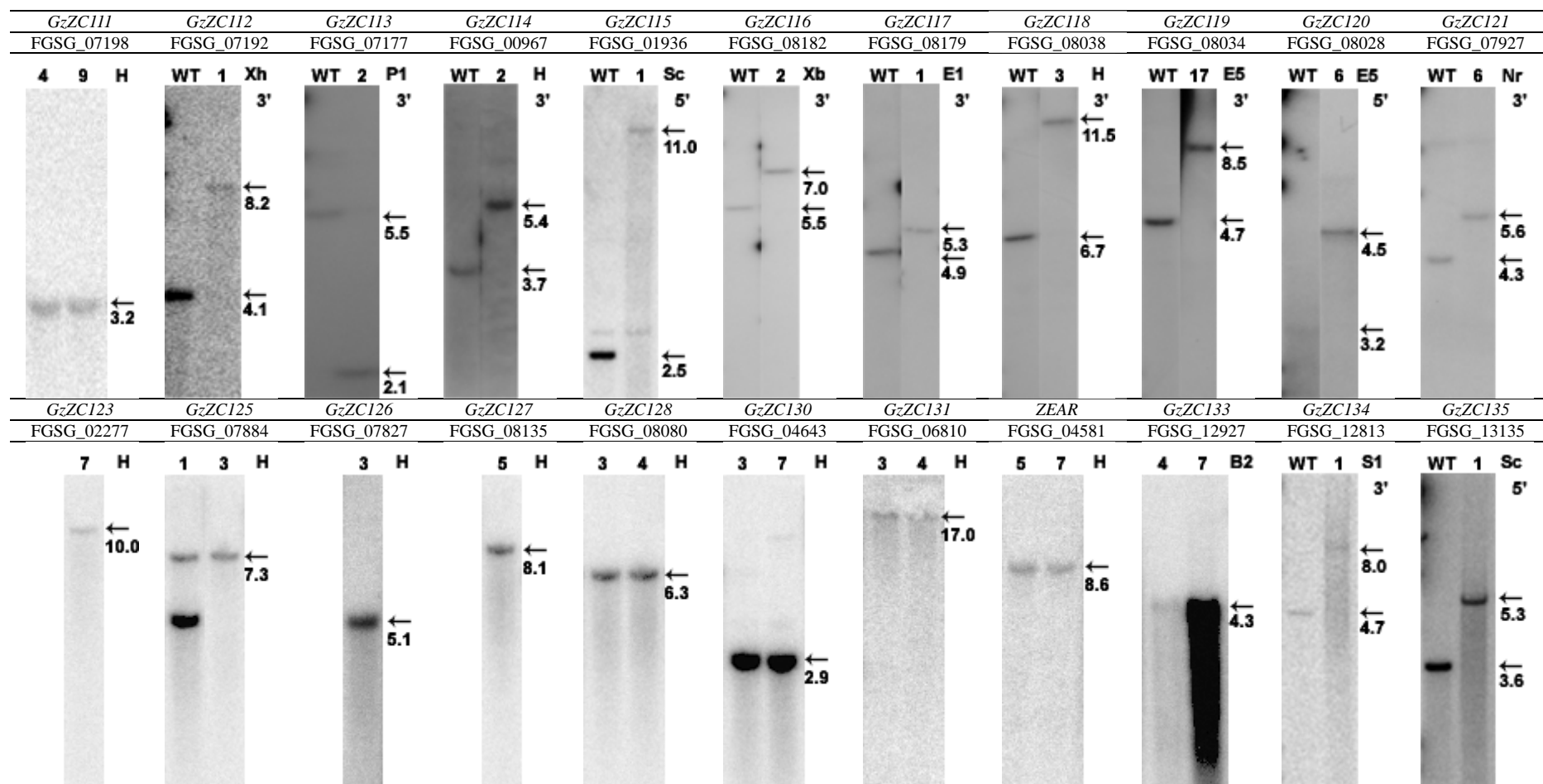

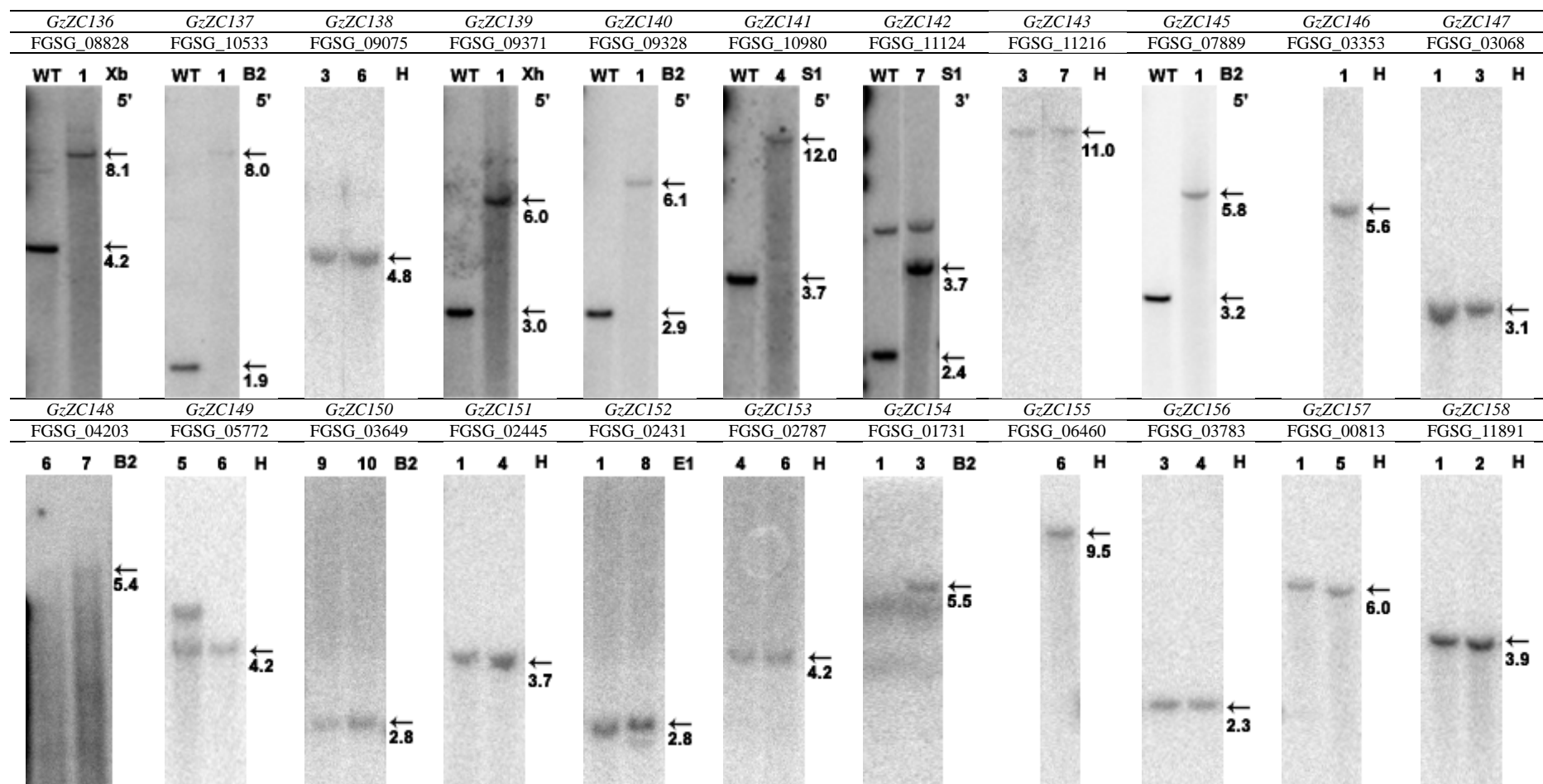

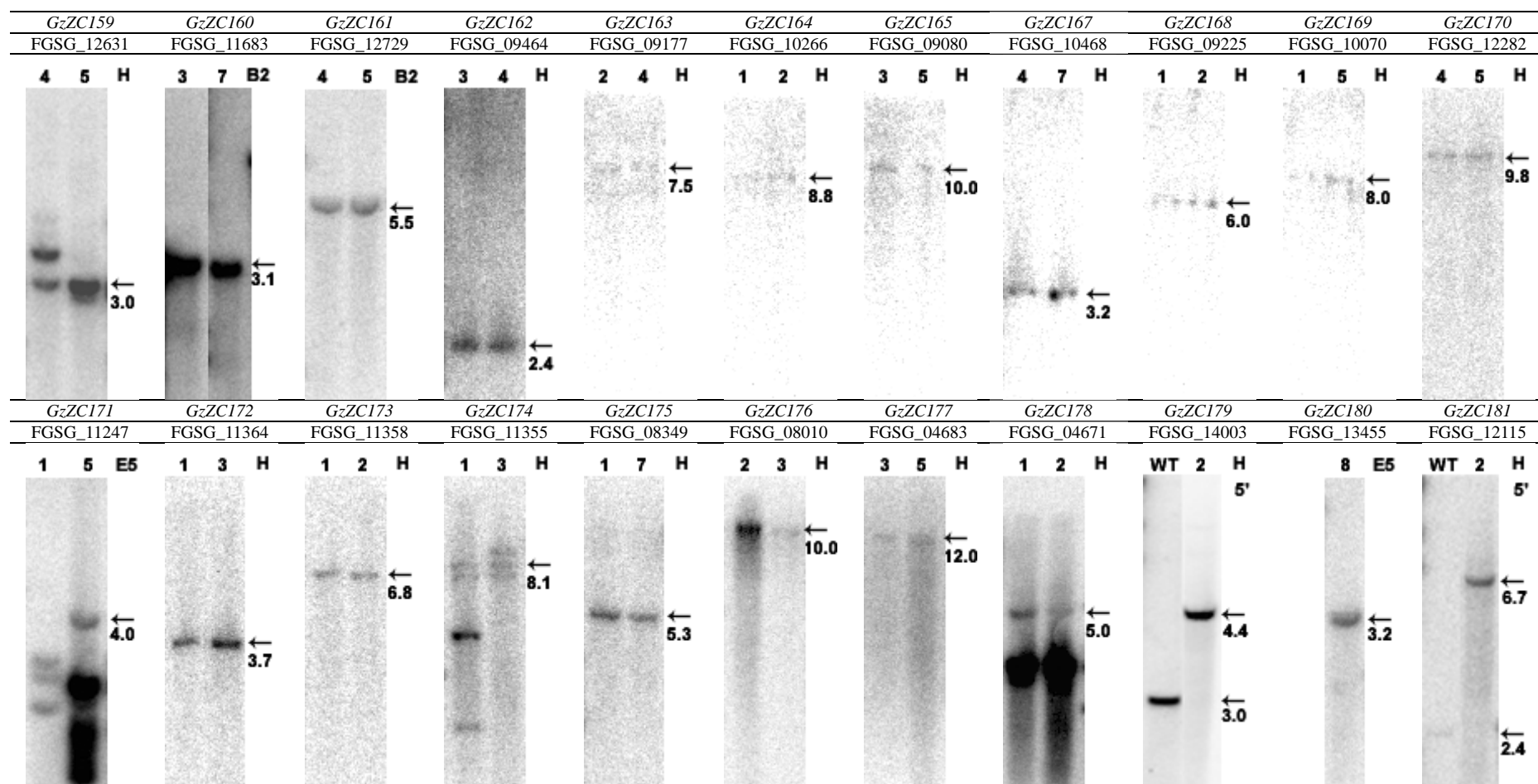

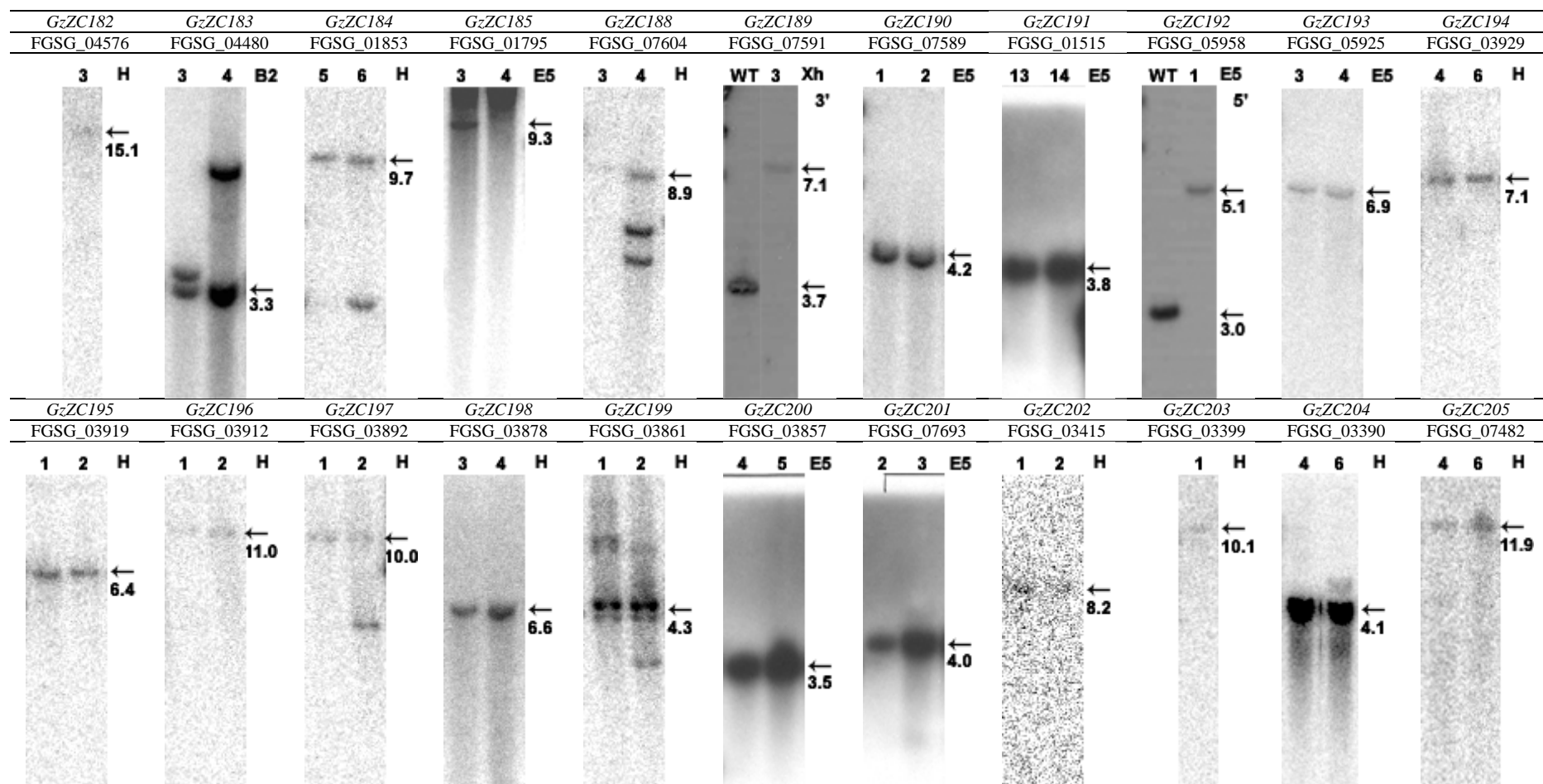

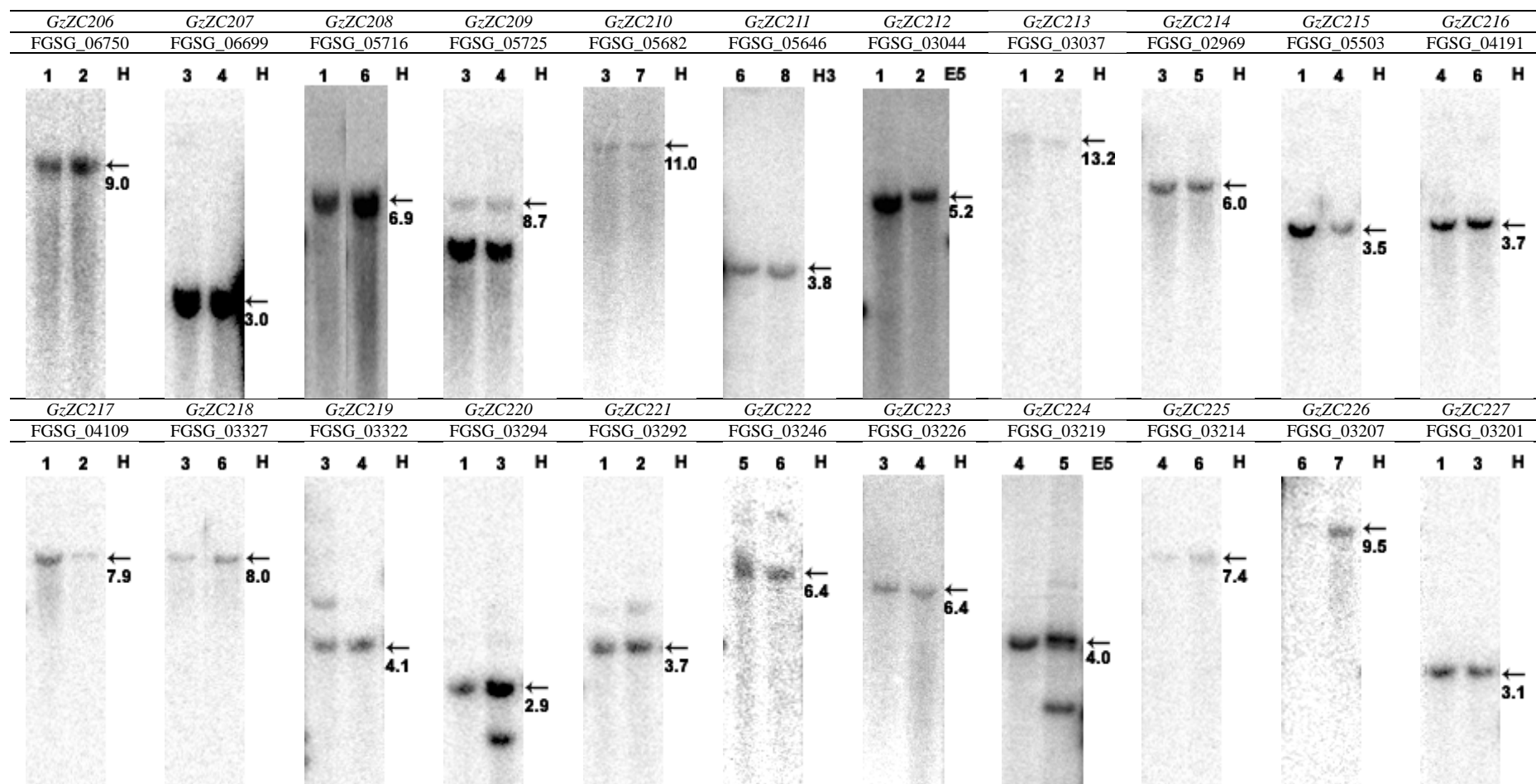

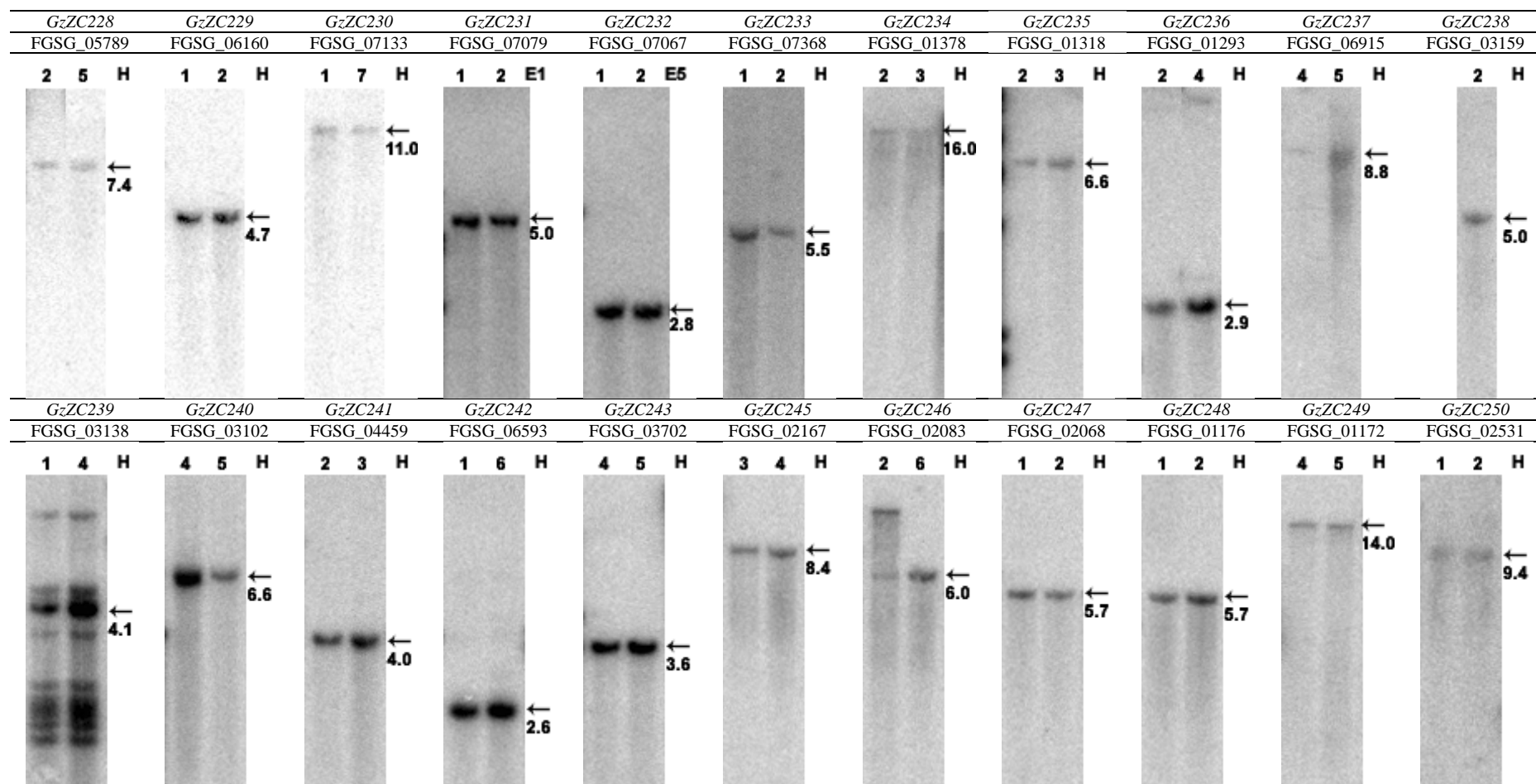

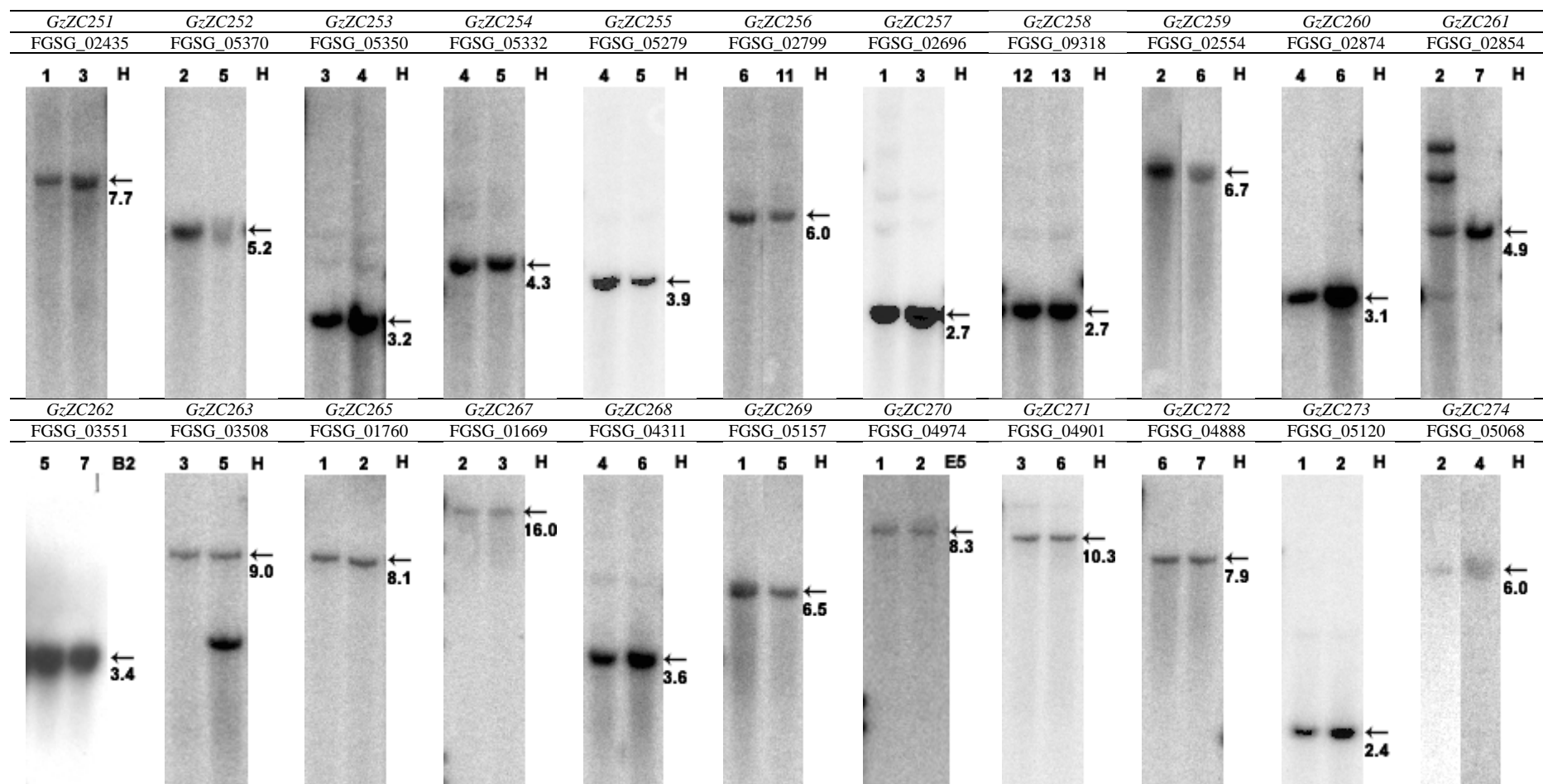

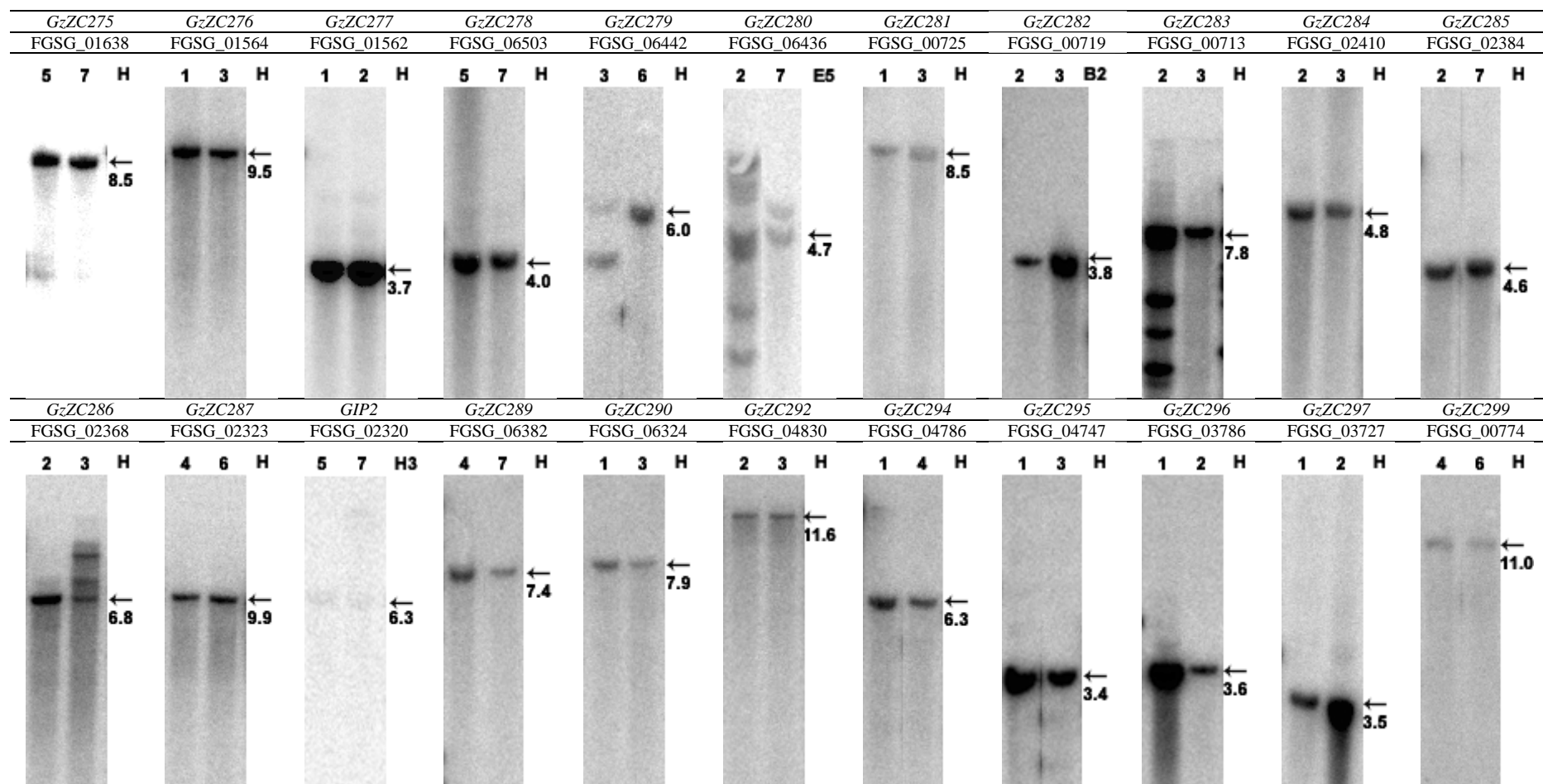

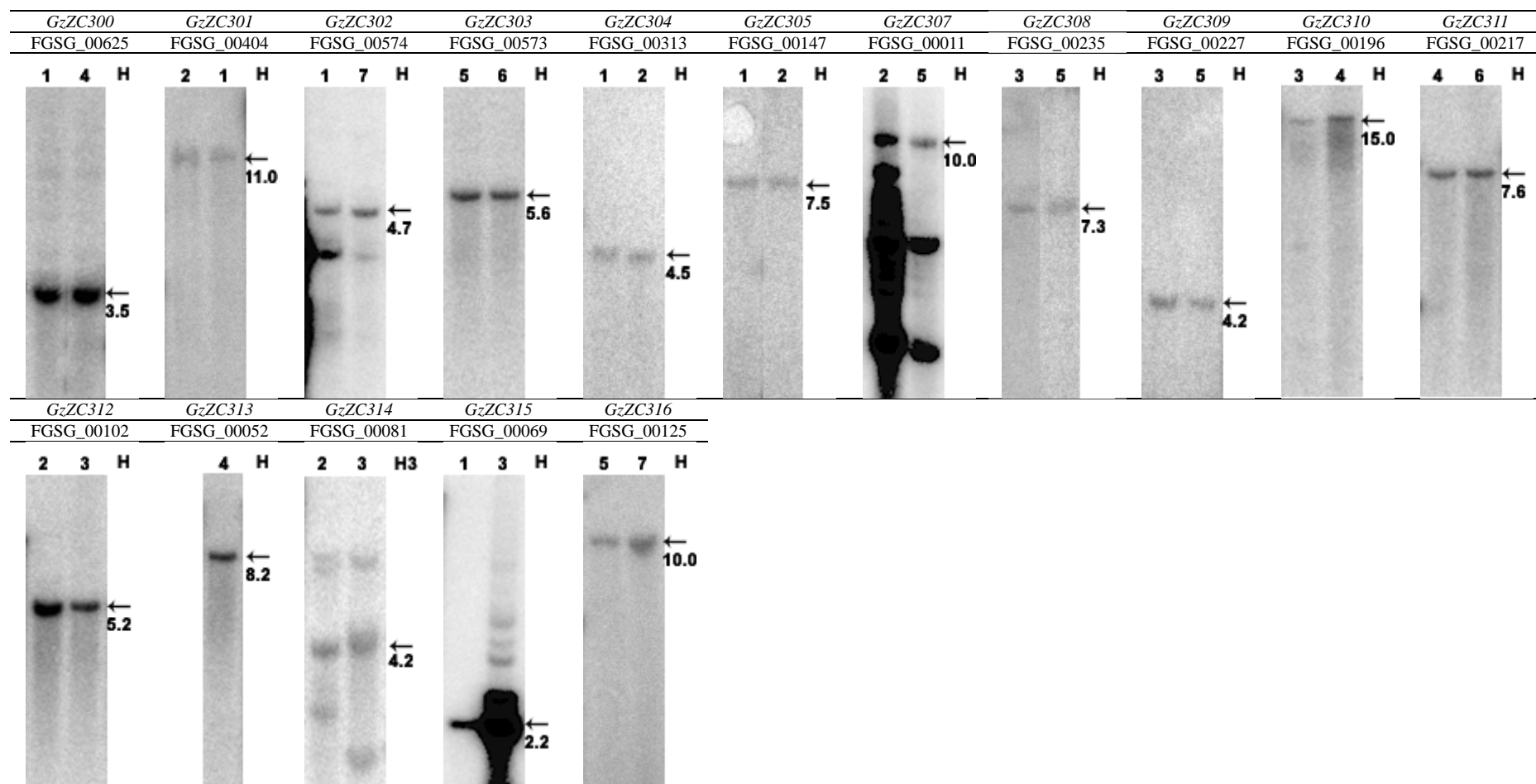

Supplement: Figure S1 — Deletion confirmation of deletion mutants by Southern blot. Restriction enzymes used for each blot and the size of DNA standards (kb) are indicated on the right of each blot. WT, G. zeae wild-type strain GZ3639; H, HindIII; E1, EcoRI, E5, EcoRV; B, BglII; S, SalI; Sc, SacI; Nd, NdeI; Nr, NruI; B1, BamHI; Xh, XhoI; Xb, XbaI; P1, PstI; C1, ClaI; SS1, SspI; K1, KpnI. (PDF) [file ppat.1002310.s001.pdf]
