# Supplementary material for: A Phenome-Based Functional Analysis of Transcription Factors in the Cereal Head Blight Fungus, Fusarium graminearum
Source: PLoS Pathog. 2011 Oct 20;7(10):e1002310. doi: 10.1371/journal.ppat.1002310 (PMC3197617; doi:10.1371/journal.ppat.1002310)
Supplement: Figure S2 — Mycelia growth of G. zeae strains on potato dextrose agar (PDA). Fungal strains were grown on PDA for five days. WT, G. zeae wild-type strain GZ3639. (PDF) [file ppat.1002310.s002.pdf]

WT	*GzAPSES001*	*FgStuA*	*GzAPSES004*	*GzAT001*	*GzbHLH001*
	FGSG_04220	FGSG_10129	FGSG_10384	FGSG_06071	FGSG_00545
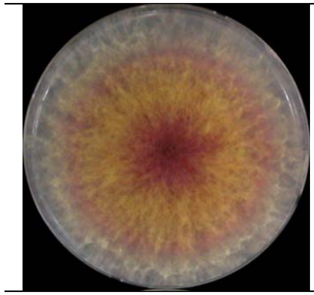	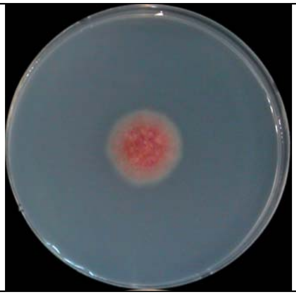	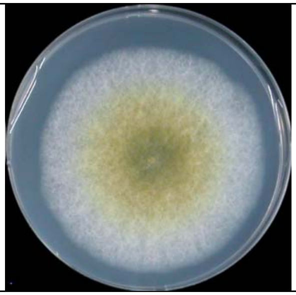	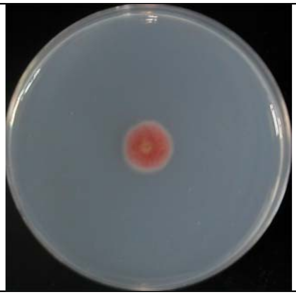	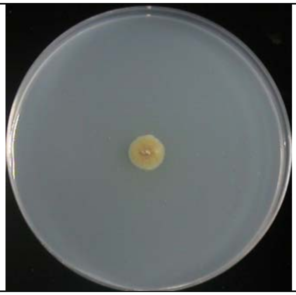	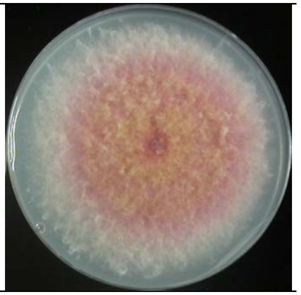
GzbHLH004	*GzBrom002*	*GzbZIP001*	*ZIF1*	*GzbZIP007*	*GzbZIP010*
FGSG_01173	FGSG_06291	FGSG_00515	FGSG_01555	FGSG_05171	FGSG_06651
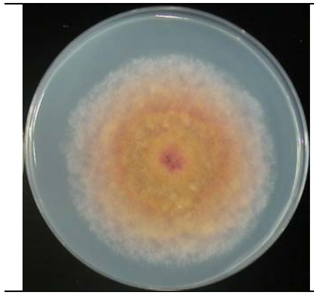	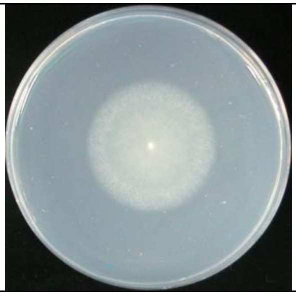	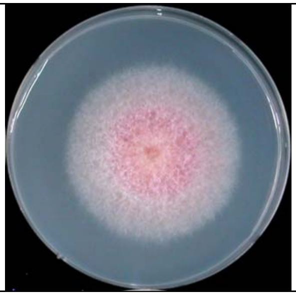	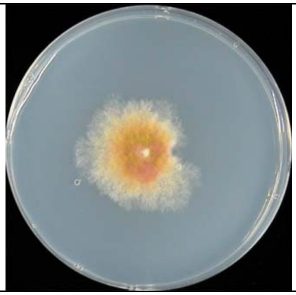	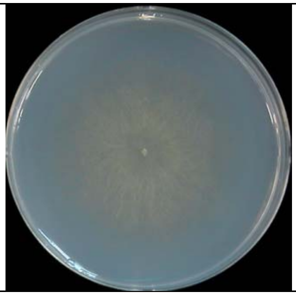	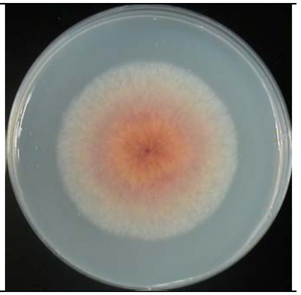
GzbZIP011	*GzbZIP016*	*GzC2H003*	*GzC2H007*	*GzC2H014*	*GzCON7*
FGSG_07789	FGSG_09832	FGSG_00477	FGSG_01022	FGSG_01350	FGSG_04134
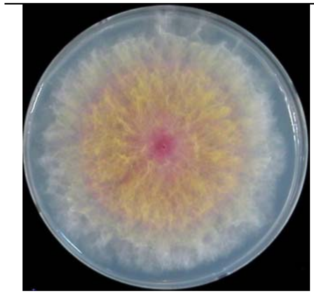	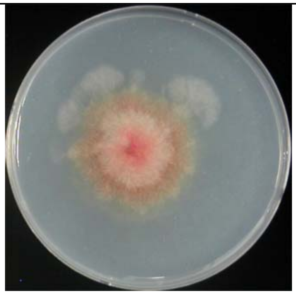	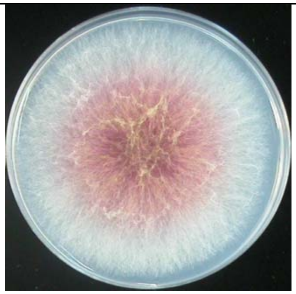	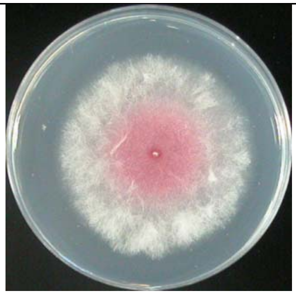	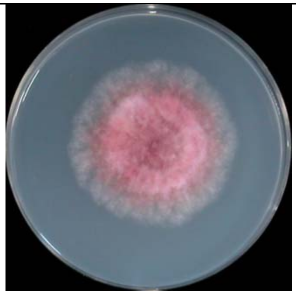	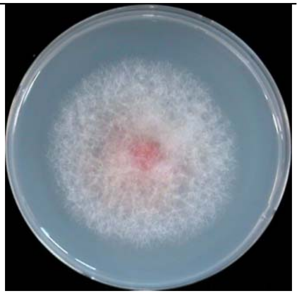

GzC2H045	*GzC2H047*	*GzC2H059*	*GzC2H066*	*GzC2H076*	*GzC2H088*
FGSG_06871	FGSG_07052	FGSG_07928	FGSG_08617	FGSG_09410	FGSG_10470
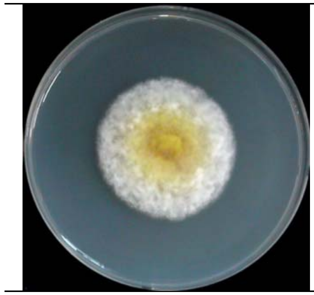	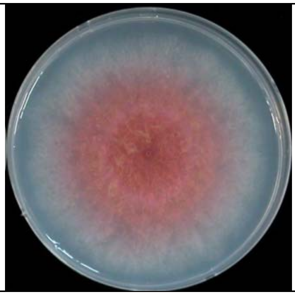	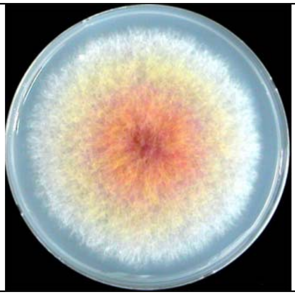	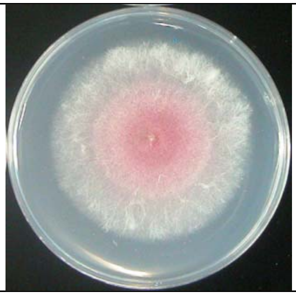	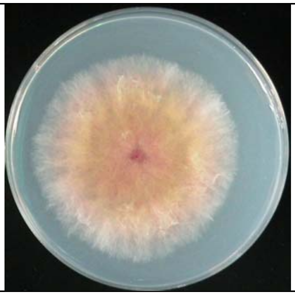	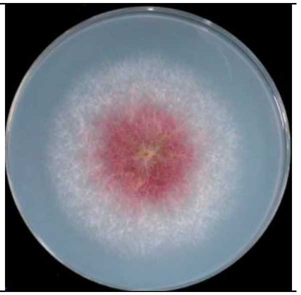
GzC2H090	*FgPac1*	*GzC2H105*	*GzNot002*	*GzDDT*	*GzGATA006*
FGSG_10517	FGSG_12970	FGSG_13711	FGSG_13746	FGSG_02527	FGSG_08634
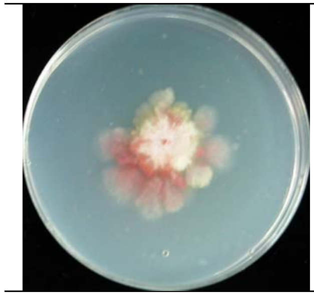	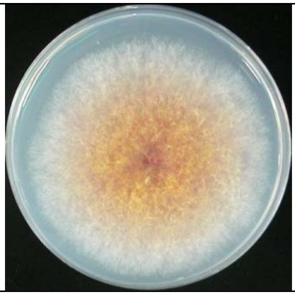	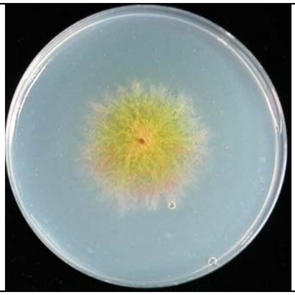	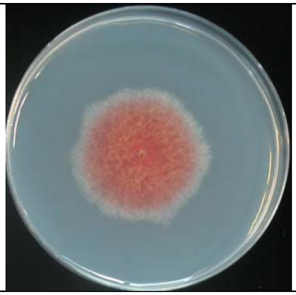	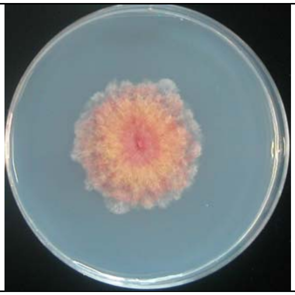	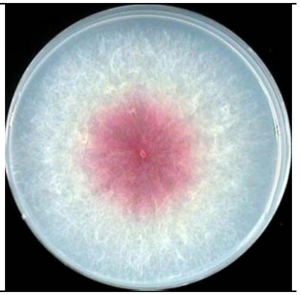
GzHSF003	*GzCCAAT002*	*GzCCAAT004*	*GzHMG002*	*GzHMG021*	*GzHMG029*
FGSG_10868	FGSG_01182	FGSG_05304	FGSG_00385	FGSG_07116	FGSG_09868
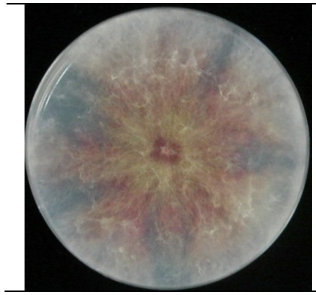	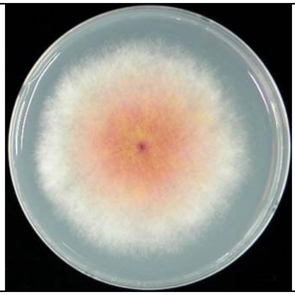	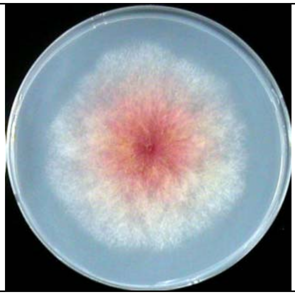	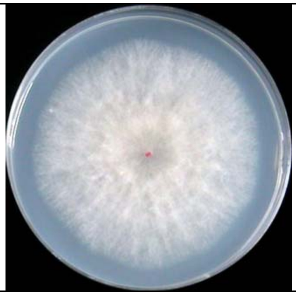	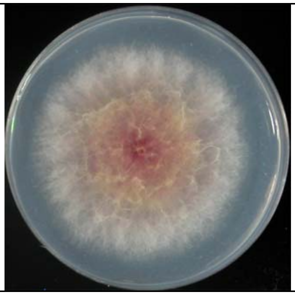	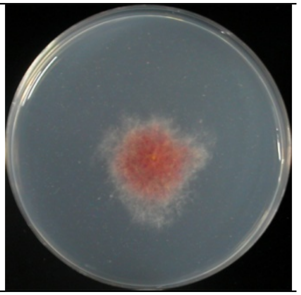

GzHOME009	*Gzscp*	*GzSsu72*	*GzMADS003*	*GzMyb002*	*GzFlbD*
FGSG_09019	FGSG_06948	FGSG_00930	FGSG_09339	FGSG_00324	FGSG_01915
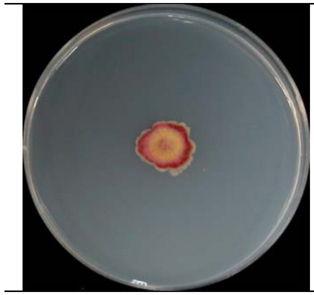	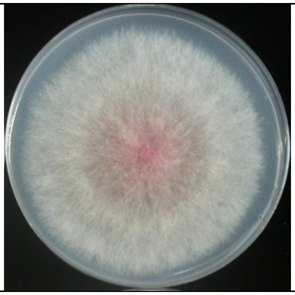	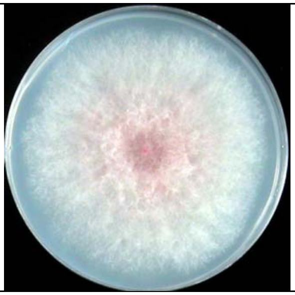	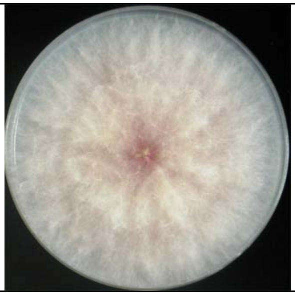	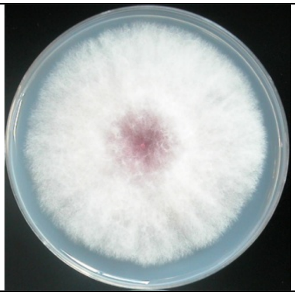	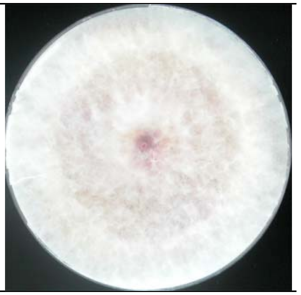
MYT2	*GzMyb016*	*GzMyb017*	*GzNH001*	*GzOB038*	*GzOB047*
FGSG_07546	FGSG_10269	FGSG_12781	FGSG_09992	FGSG_09654	FGSG_13120
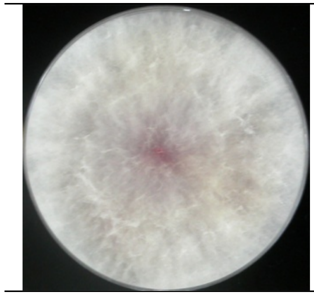	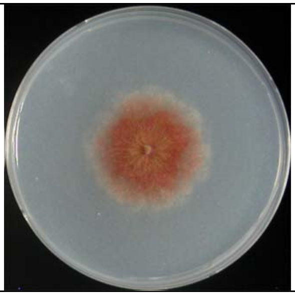	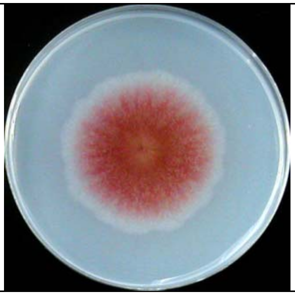	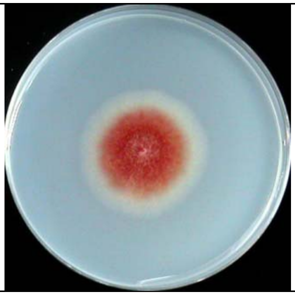	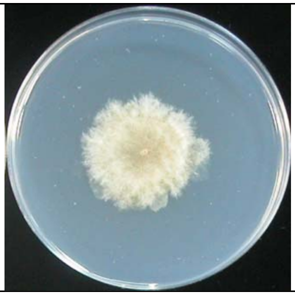	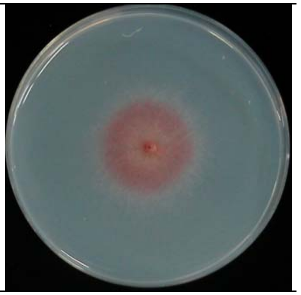
GzP53L003	*GzTF2S001*	*FgFSR1*	*GzWing014*	*GzWing015*	*GzRFX1*
FGSG_07157	FGSG_00902	FGSG_01665	FGSG_06359	FGSG_06944	FGSG_07420
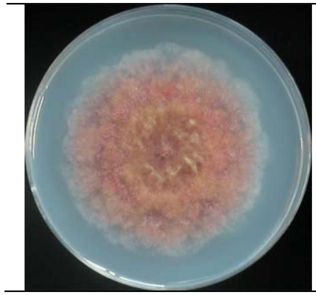	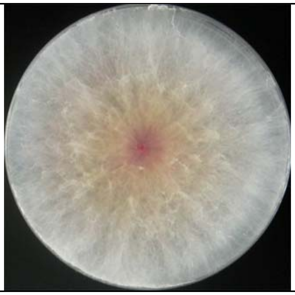	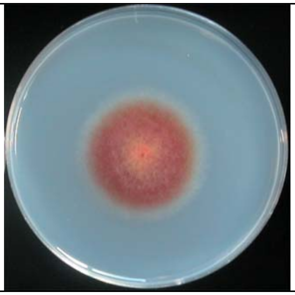	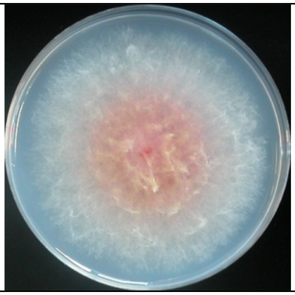	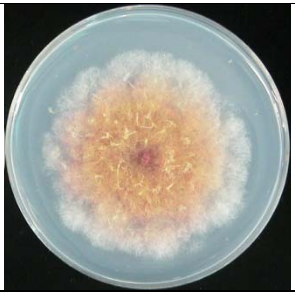	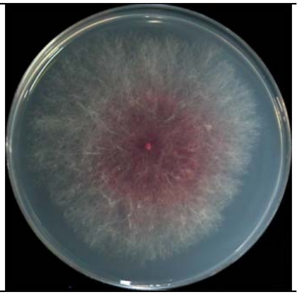

GzWing017	*GzWing018*	*GzWing019*	*GzWing020*	*GzCCHC011*	*GzZC086*
FGSG_07433	FGSG_08481	FGSG_08572	FGSG_08719	FGSG_10716	FGSG_08924
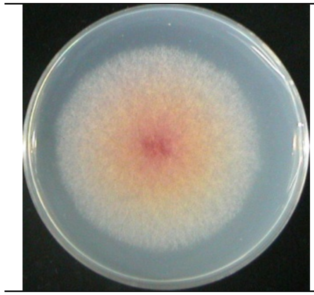	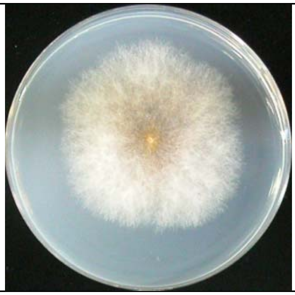	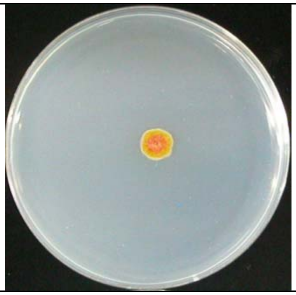	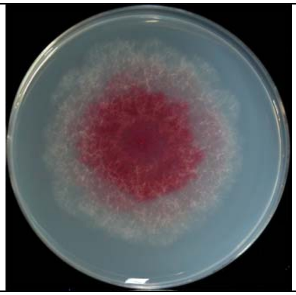	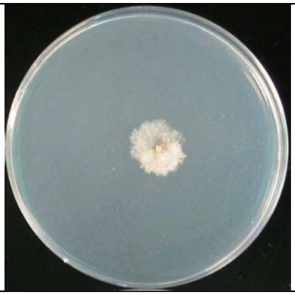	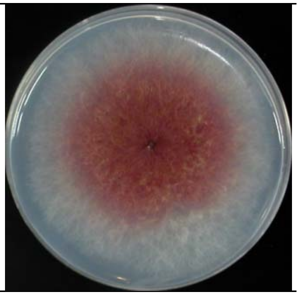
GzZC087	*GzZC108*	*GzZC229*	*GzZC232*	*GzZC246*	*GzZC250*
FGSG_10069	FGSG_08769	FGSG_06160	FGSG_07067	FGSG_02083	FGSG_02531
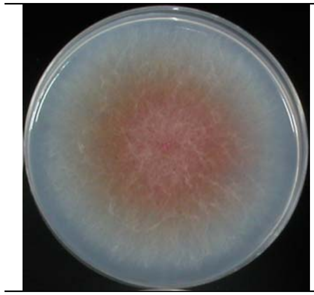	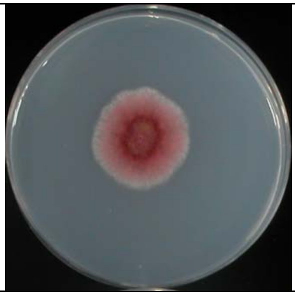	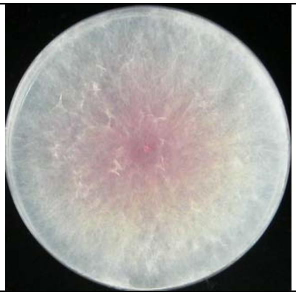	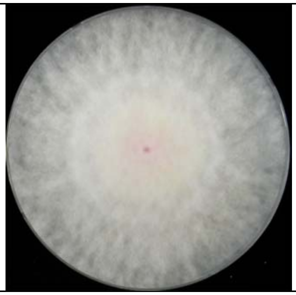	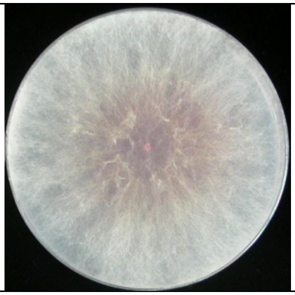	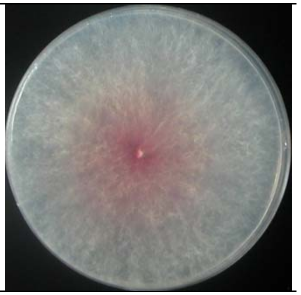
GzZC271	*GIP2*	*GzZC302*	*GzZC303*		
FGSG_04901	FGSG_02320	FGSG_00574	FGSG_00573		
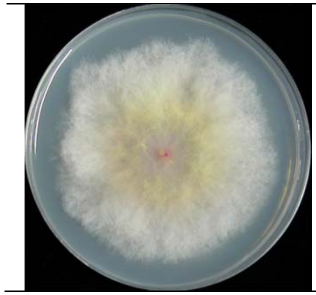	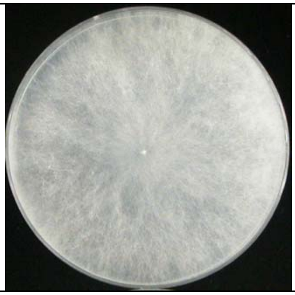	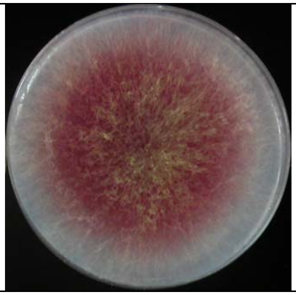	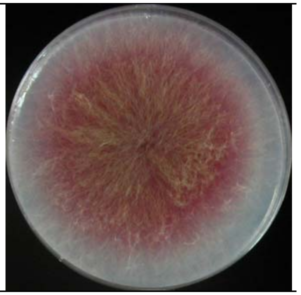		
