# Supplementary material for: A Phenome-Based Functional Analysis of Transcription Factors in the Cereal Head Blight Fungus, Fusarium graminearum
Source: PLoS Pathog. 2011 Oct 20;7(10):e1002310. doi: 10.1371/journal.ppat.1002310 (PMC3197617; doi:10.1371/journal.ppat.1002310)
Supplement: Figure S3 — Mycelia growth of G. zeae strains on minimal media (MM). Fungal strains were grown on MM for five days. WT, G. zeae wild-type strain GZ3639. (PDF) [file ppat.1002310.s003.pdf]

WT	*GzAPSES001*	*FgStuA*	*GzAPSES004*	*GzAT001*	*GzbHLH001*
	FGSG_04220	FGSG_10129	FGSG_10384	FGSG_06071	FGSG_00545
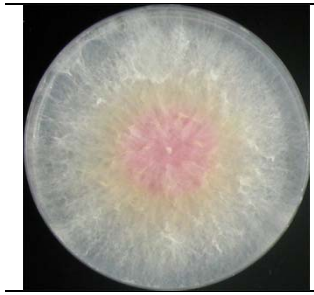	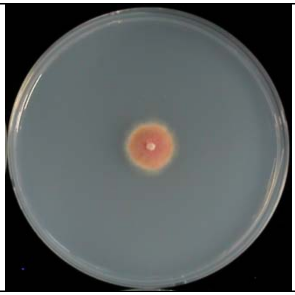	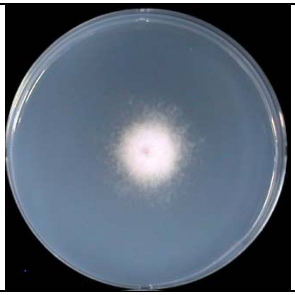	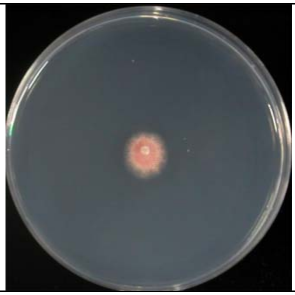	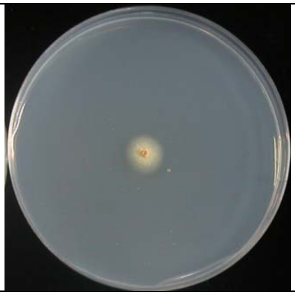	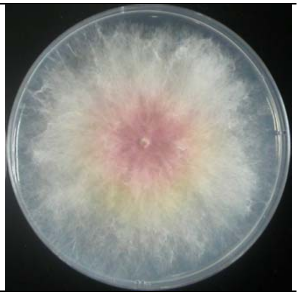
GzbHLH002	*GzbHLH004*	*GzBrom002*	*GzbZIP001*	*ZIF1*	*GzbZIP007*
FGSG_00750	FGSG_01173	FGSG_06291	FGSG_00515	FGSG_01555	FGSG_05171
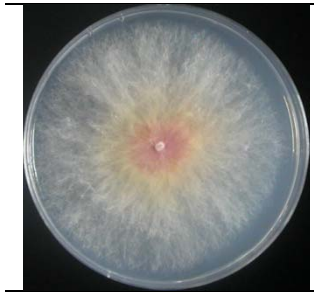	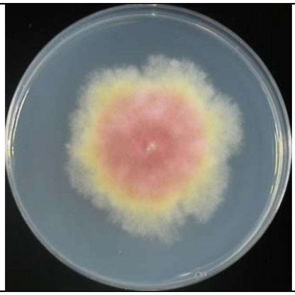	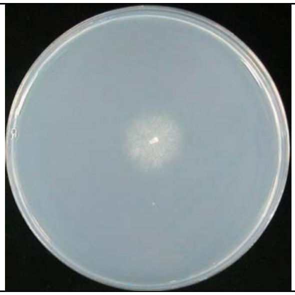	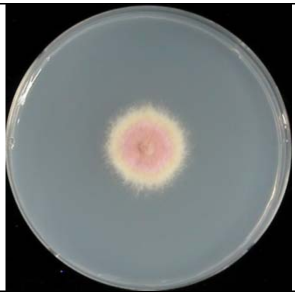	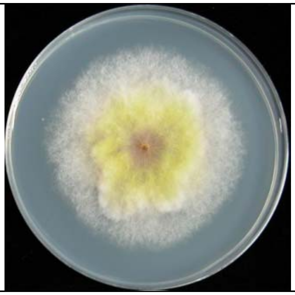	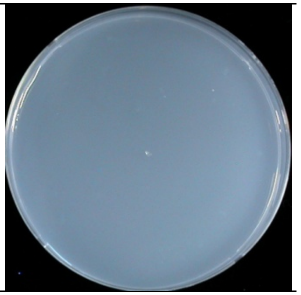
GzbZIP010	*GzbZIP016*	*GzC2H003*	*GzC2H014*	*GzCON7*	*GzC2H045*
FGSG_06651	FGSG_09832	FGSG_00477	FGSG_01350	FGSG_04134	FGSG_06871
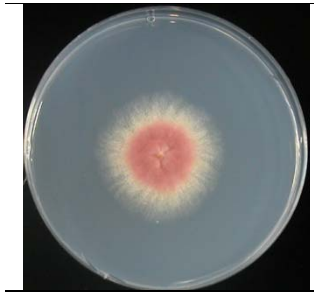	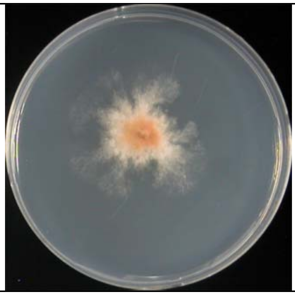	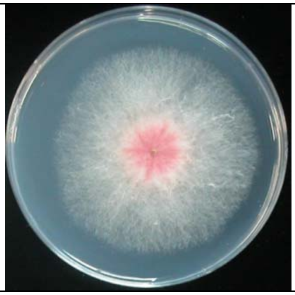	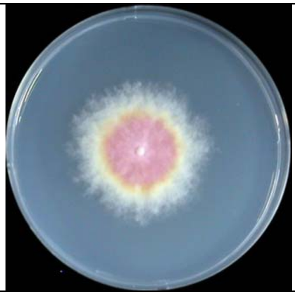	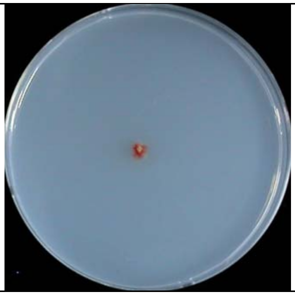	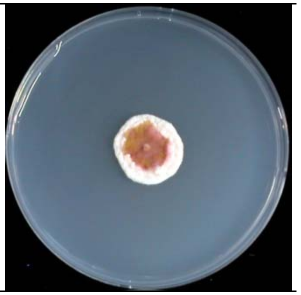

GzC2H048	*GzC2H059*	*GzC2H088*	*GzC2H090*	*GzC2H095*	*FgPac1*
FGSG_07075	FGSG_07928	FGSG_10470	FGSG_10517	FGSG_11799	FGSG_12970
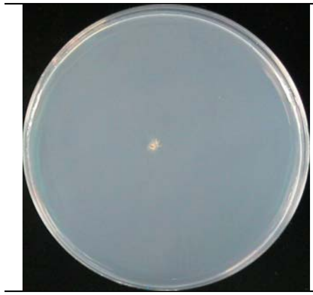	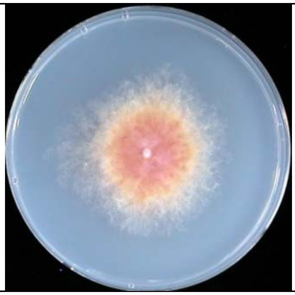	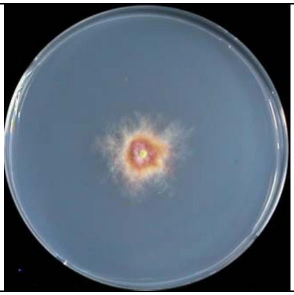	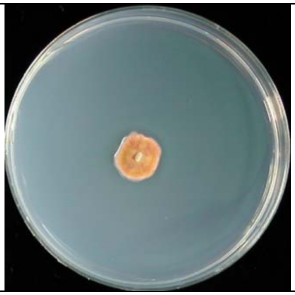	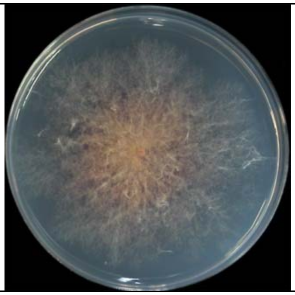	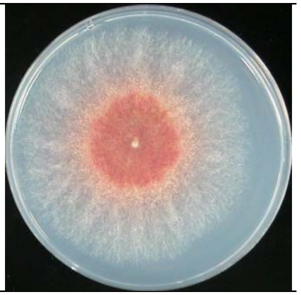
GzC2H105	*GzNot002*	*GzDDT*	*GzGATA006*	*GzGATA007*	*GzHSF003*
FGSG_13711	FGSG_13746	FGSG_02527	FGSG_08634	FGSG_09565	FGSG_10868
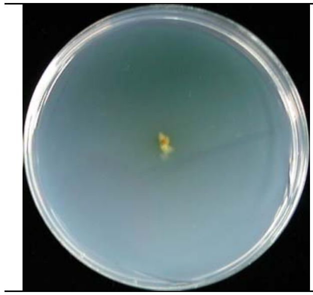	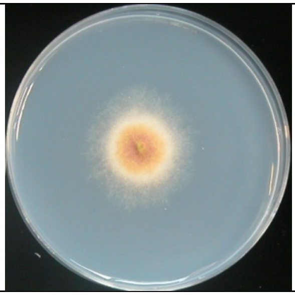	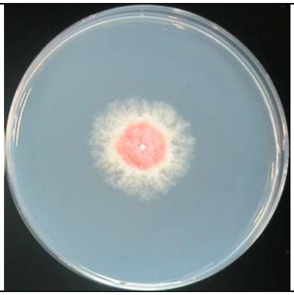	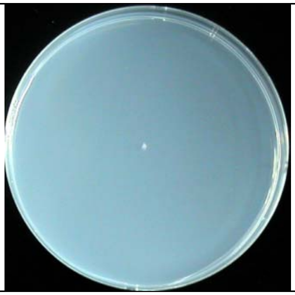	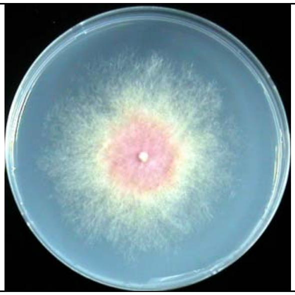	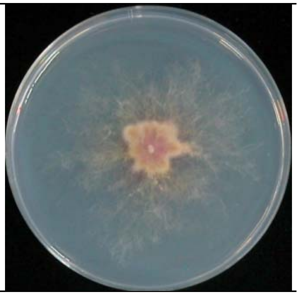
GzCCAAT002	*GzCCAAT004*	*GzHMG002*	*GzHMG021*	*GzHMG029*	*GzHOME009*
FGSG_01182	FGSG_05304	FGSG_00385	FGSG_07116	FGSG_09868	FGSG_09019
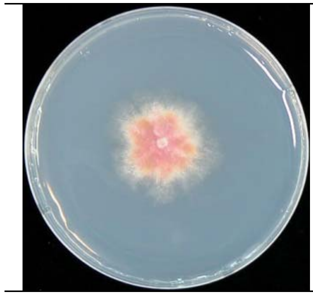	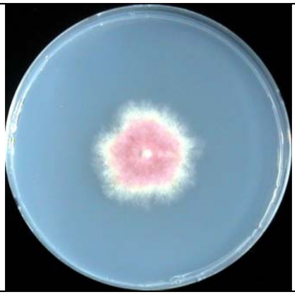	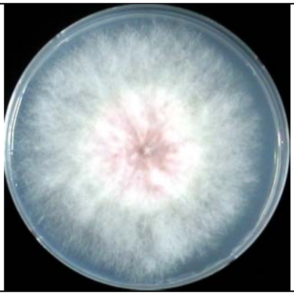	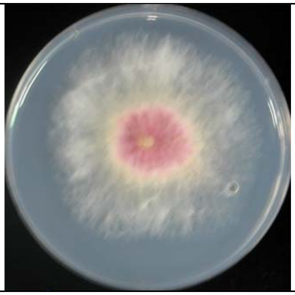	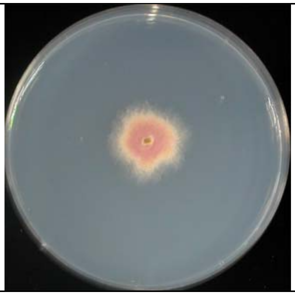	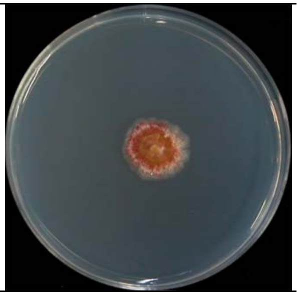

Gzscp	*GzSsu72*	*GzMyb002*	*GzFlbD*	*MYT2*	*GzMyb016*
FGSG_06948	FGSG_00930	FGSG_00324	FGSG_01915	FGSG_07546	FGSG_10269
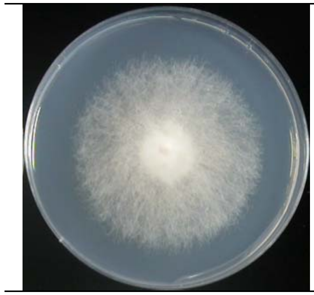	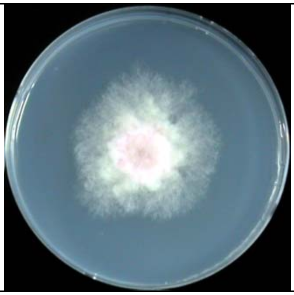	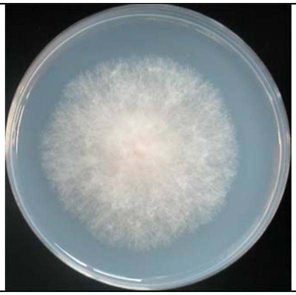	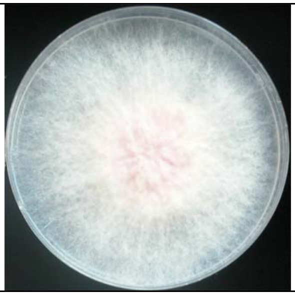	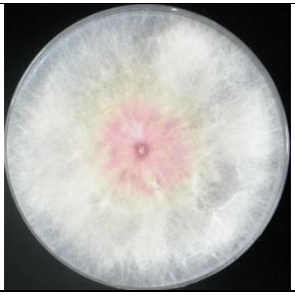	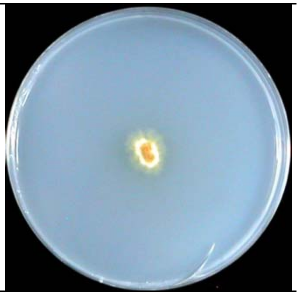
GzMyb017	*GzNH001*	*GzOB038*	*GzOB047*	*GzTF2S001*	*FgFSR1*
FGSG_12781	FGSG_09992	FGSG_09654	FGSG_13120	FGSG_00902	FGSG_01665
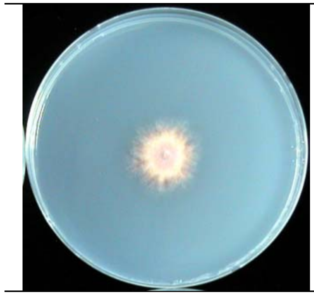	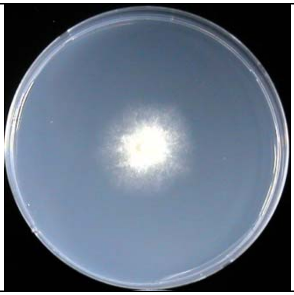	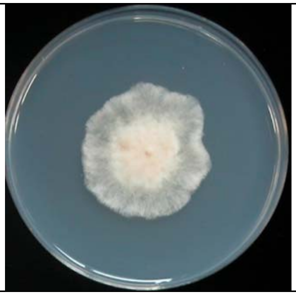	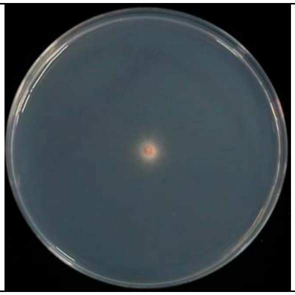	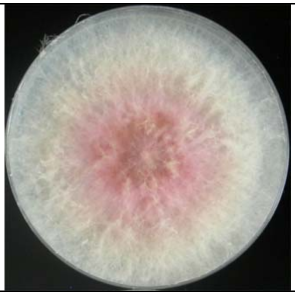	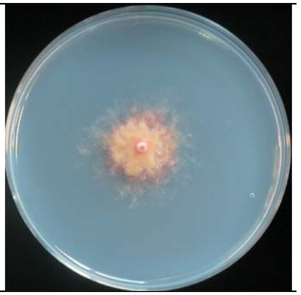
GzWing011	*GzWing015*	*GzRFX1*	*GzWing017*	*GzWing018*	*GzWing019*
FGSG_05520	FGSG_06944	FGSG_07420	FGSG_07433	FGSG_08481	FGSG_08572
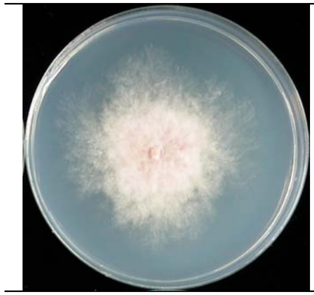	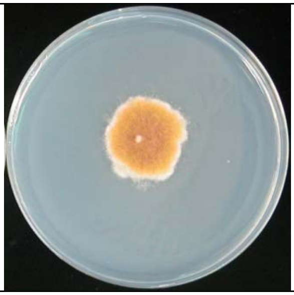	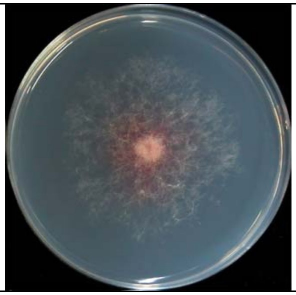	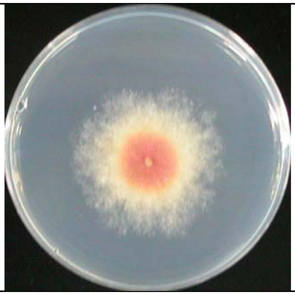	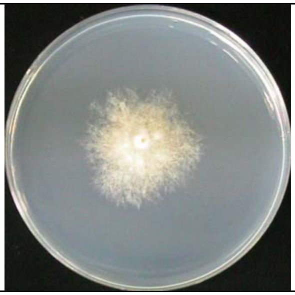	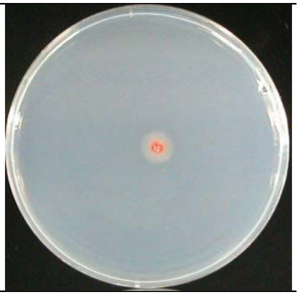

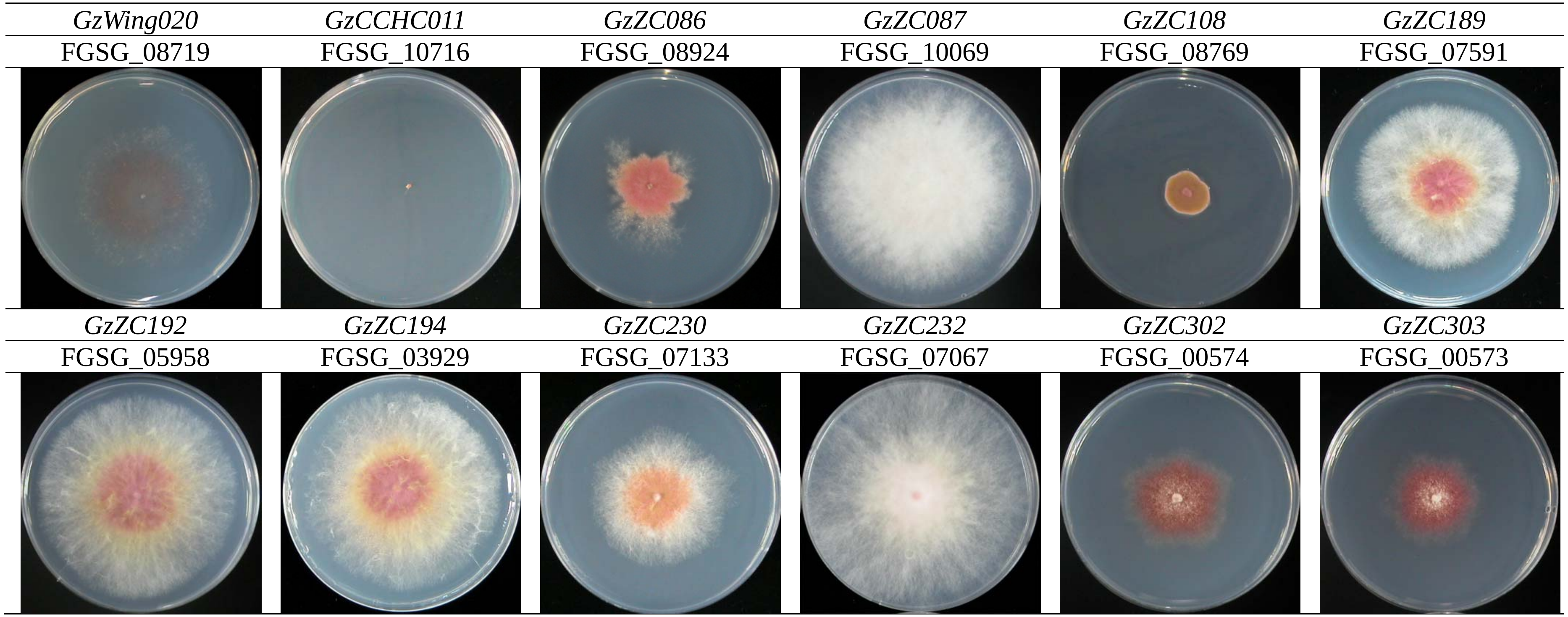
GzWing020
FGSG_08719
GzCCHC011
FGSG_10716
GzZC086
FGSG_08924
GzZC087
FGSG_10069
GzZC108
FGSG_08769
GzZC189
FGSG_07591
GzZC192
FGSG_05958
GzZC194
FGSG_03929
GzZC230
FGSG_07133
GzZC232
FGSG_07067
GzZC302
FGSG_00574
GzZC303
FGSG_00573
