# Supplementary material for: A Phenome-Based Functional Analysis of Transcription Factors in the Cereal Head Blight Fungus, Fusarium graminearum
Source: PLoS Pathog. 2011 Oct 20;7(10):e1002310. doi: 10.1371/journal.ppat.1002310 (PMC3197617; doi:10.1371/journal.ppat.1002310)
Supplement: Figure S4 — Sexual development of seven groups of TF mutants. Each strain was inoculated on carrot agar. The photographs were taken 10 days after sexual induction. WT, G. zeae wild-type strain GZ3639. Scale bar = 0.5 mm (dissecting microscope images) and 100 µm (differential interference contrast images). (PDF) [file ppat.1002310.s004.pdf]

Group 1. Increased number of perithecia (4)

WT	*GzbHLH007*	*GzbHLH014*	*GzbZIP010*	*GzGH*
	FGSG_02814	FGSG_09308	FGSG_06651	FGSG_06356

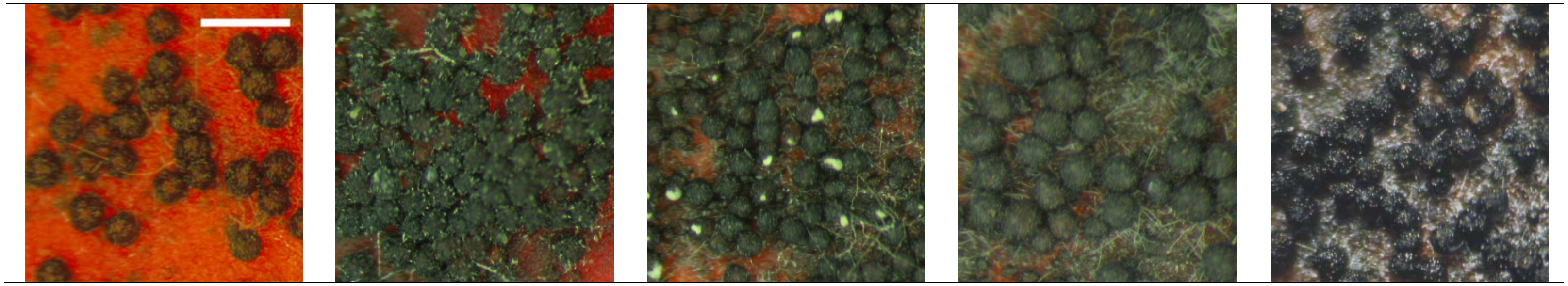

Group 2. No perithecia (44)

GzAPSES001	*FgStuA*	*GzAPSES004*	*GzAT001*	*GzbHLH004*	*GzBrom002*
FGSG_04220	FGSG_10129	FGSG_10384	FGSG_06071	FGSG_01173	FGSG_06291
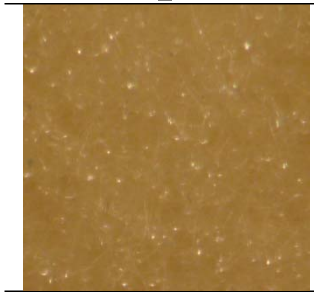	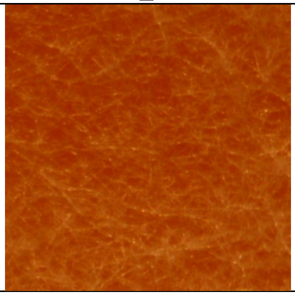	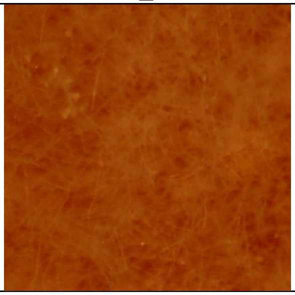	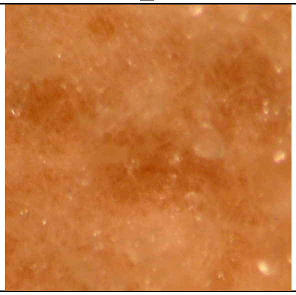	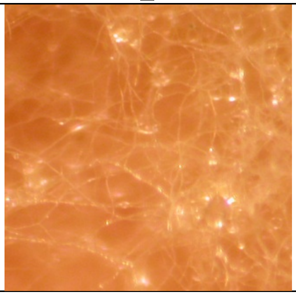	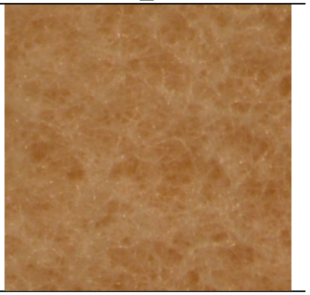
GzbZIP007	*GzC2H003*	*GzC2H007*	*GzC2H014*	*GzC2H016*	*GzCON7*
FGSG_05171	FGSG_00477	FGSG_01022	FGSG_01350	FGSG_01877	FGSG_04134
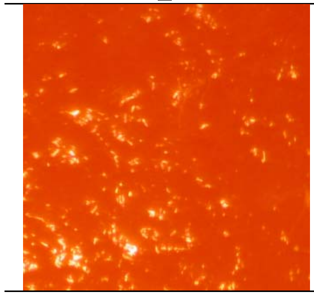	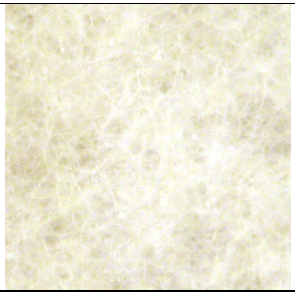	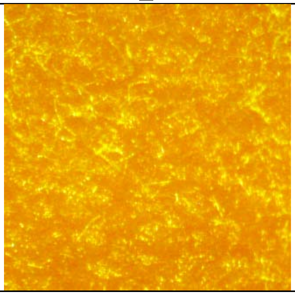	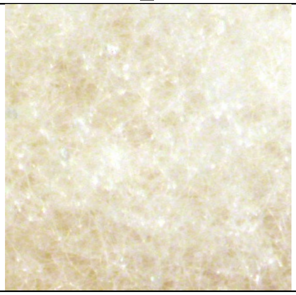	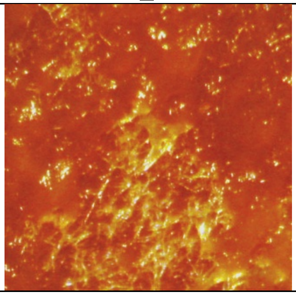	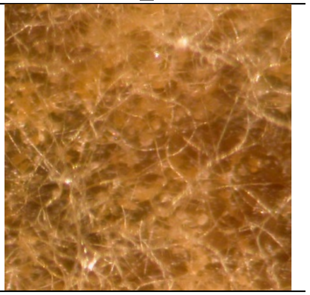
GzC2H045	*GzC2H088*	*GzC2H090*	*GzC2H094*	*GzC2H105*	*GzNot002*
FGSG_06871	FGSG_10470	FGSG_10517	FGSG_11792	FGSG_13711	FGSG_13746
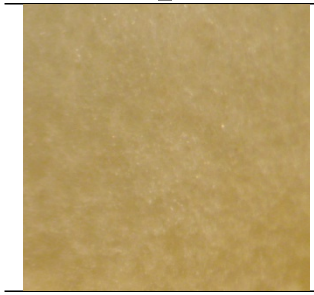	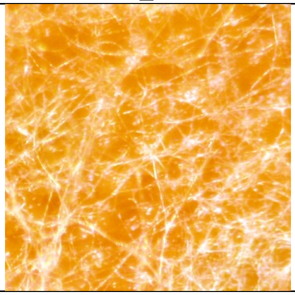	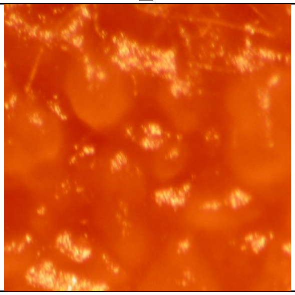	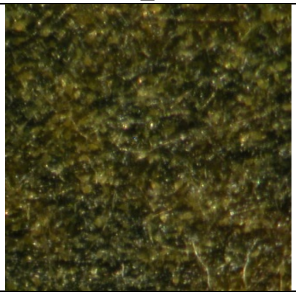	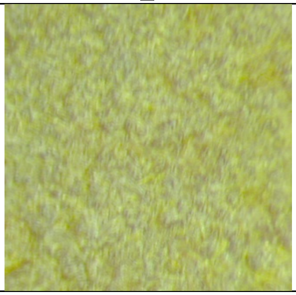	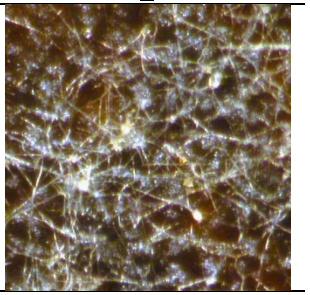

GzCCAAT004	*GzHMG002*	*Gzscp*	*GzMADS003*	*GzMyb002*	*GzFlbD*
FGSG_05304	FGSG_00385	FGSG_06948	FGSG_09339	FGSG_00324	FGSG_01915
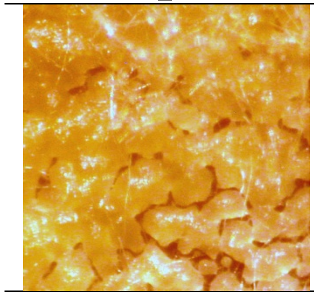	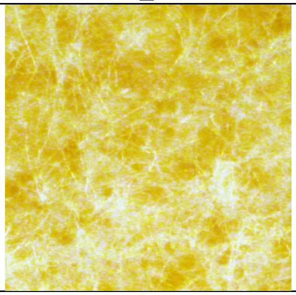	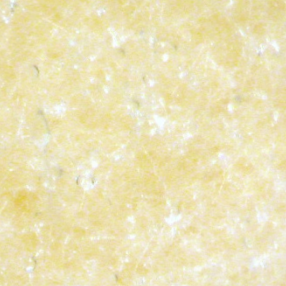	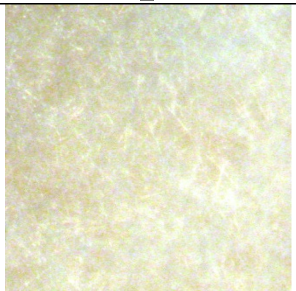	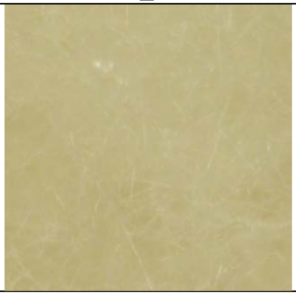	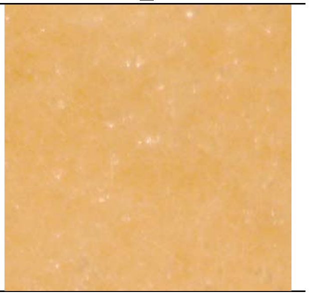
GzMyb016	*GzMyb017*	*GzNH001*	*GzOB047*	*GzP53L005*	*FgFSR1*
FGSG_10269	FGSG_12781	FGSG_09992	FGSG_13120	FGSG_09709	FGSG_01665
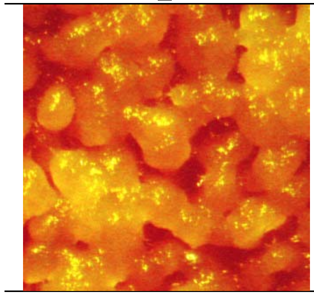	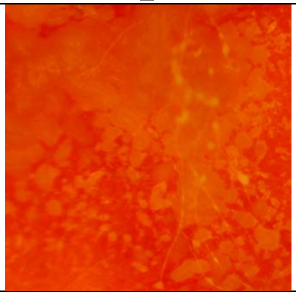	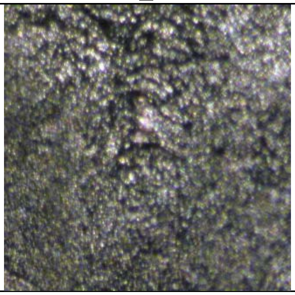	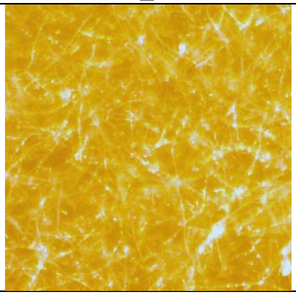	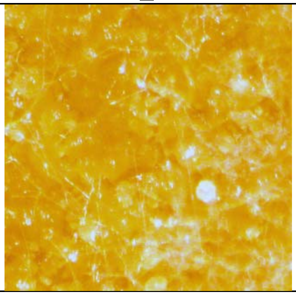	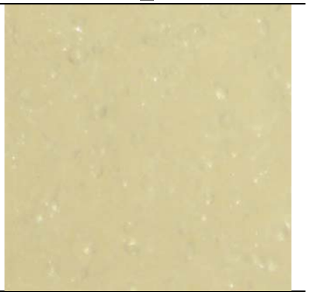
GzWing011	*GzWing018*	*GzWing019*	*GzWing020*	*GzCCHC011*	*GzDHHC003*
FGSG_05520	FGSG_08481	FGSG_08572	FGSG_08719	FGSG_10716	FGSG_06542
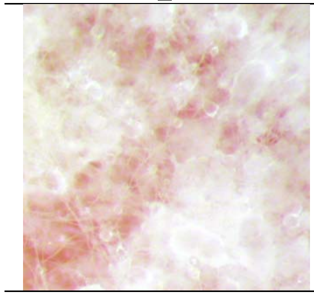	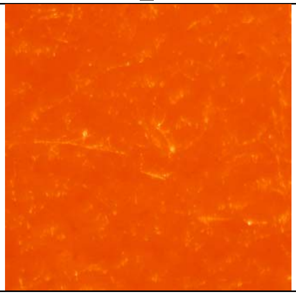	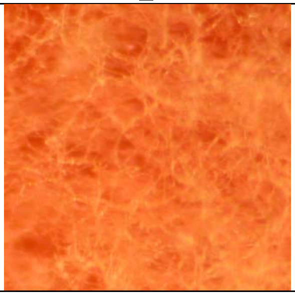	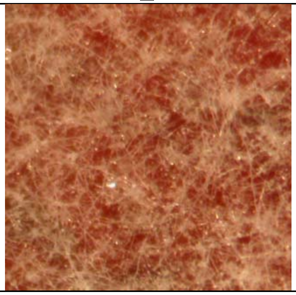	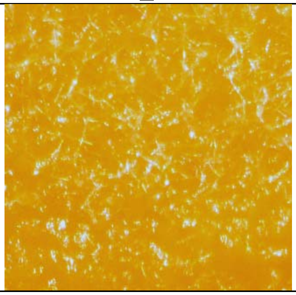	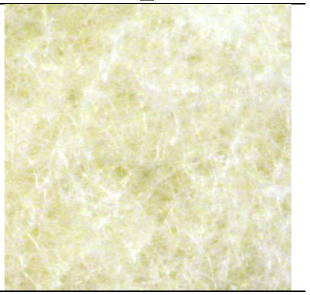

GzZC087	*GzZC108*	*GzZC183*	*GzZC232*	*GzZC258*	*GzZC301*
FGSG_10069	FGSG_08769	FGSG_04480	FGSG_07067	FGSG_09318	FGSG_00404
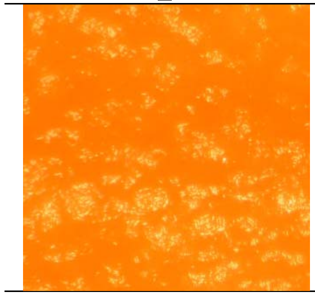	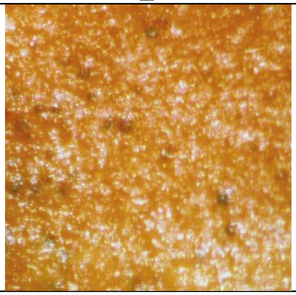	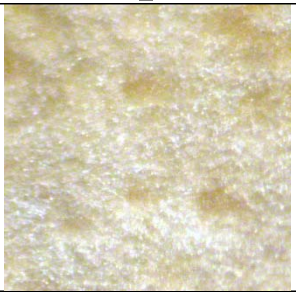	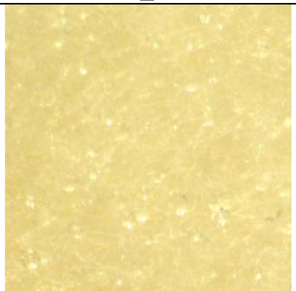	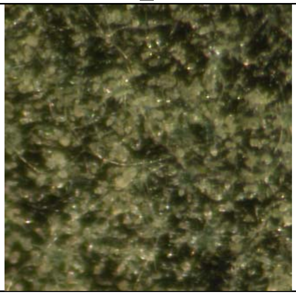	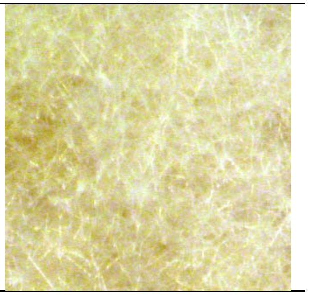

GzZC302	*GzZC303*
FGSG_00574	FGSG_00573
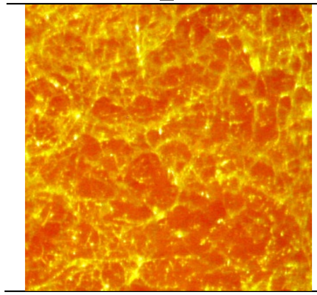	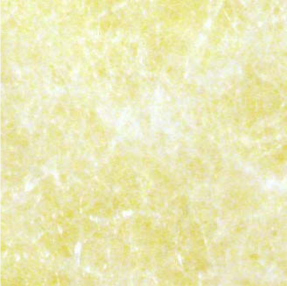

Group 3. Defect in perithecia development but not in ascospores formation (23)

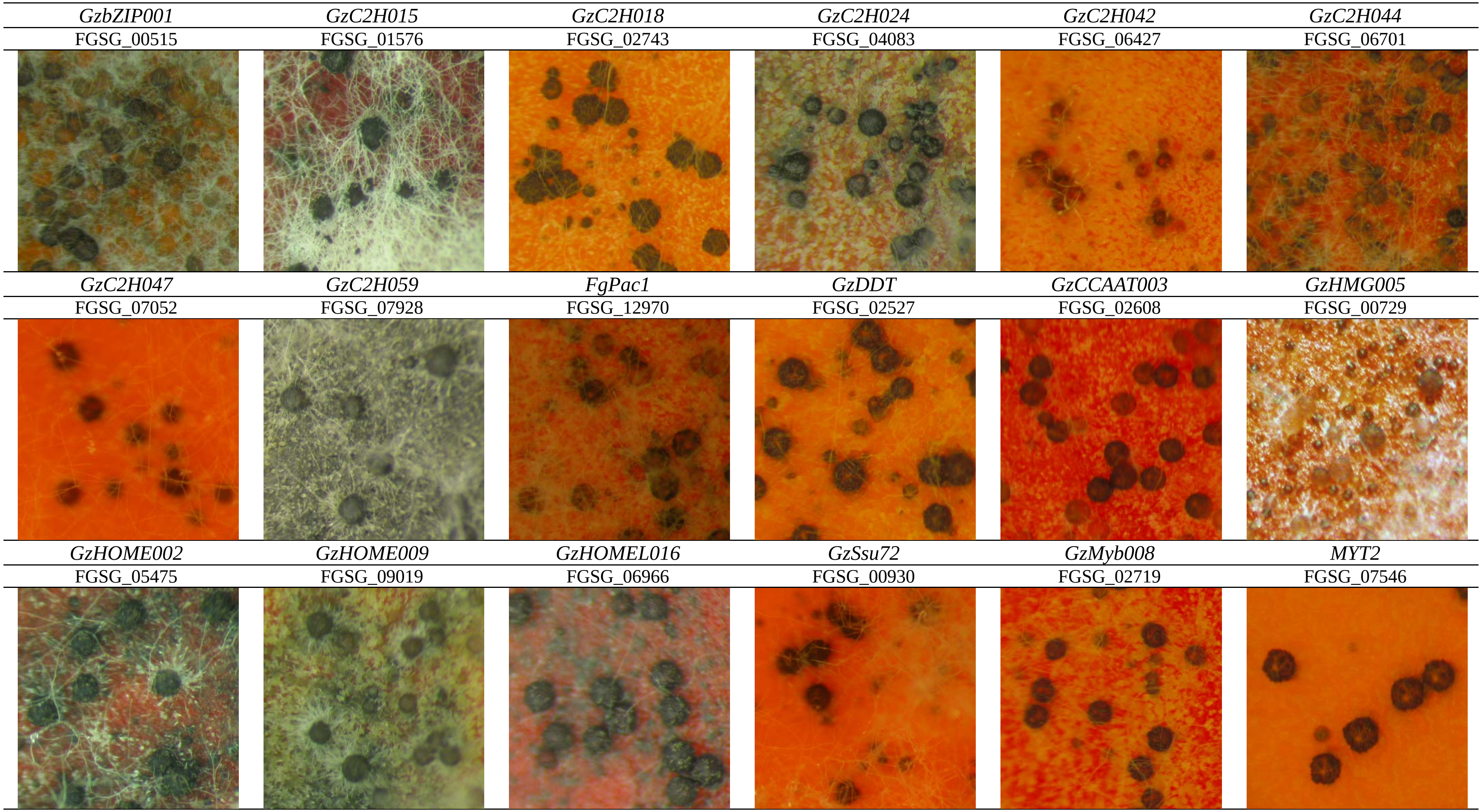

GzOB036	*GzZC228*	*GzZC248*	*GzZC276*	*GzZC289*
FGSG_09000	FGSG_05789	FGSG_01176	FGSG_01564	FGSG_06382
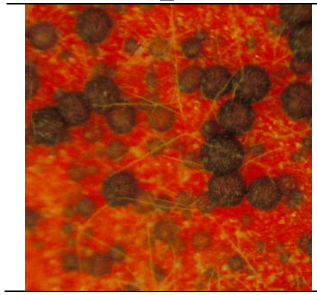	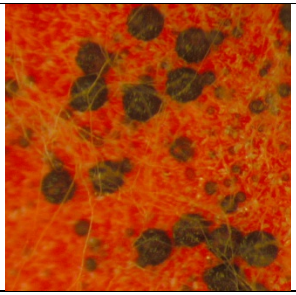	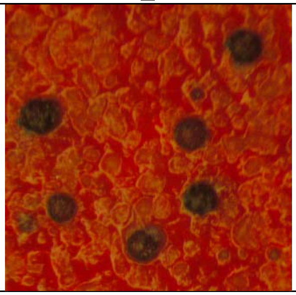	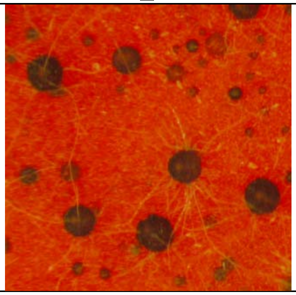	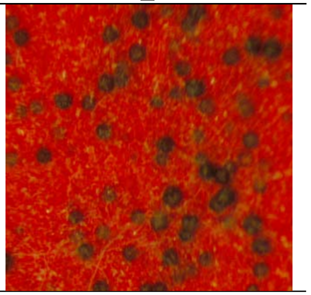

Group 4. Defects both in perithecia development and ascospores formation (9)

WT	*GzbHLH005*	*GzbZIP016*	*GzC2H008*	*GzC2H013*	*GzC2H066*
	FGSG_01307	FGSG_09832	FGSG_01106	FGSG_01341	FGSG_08617

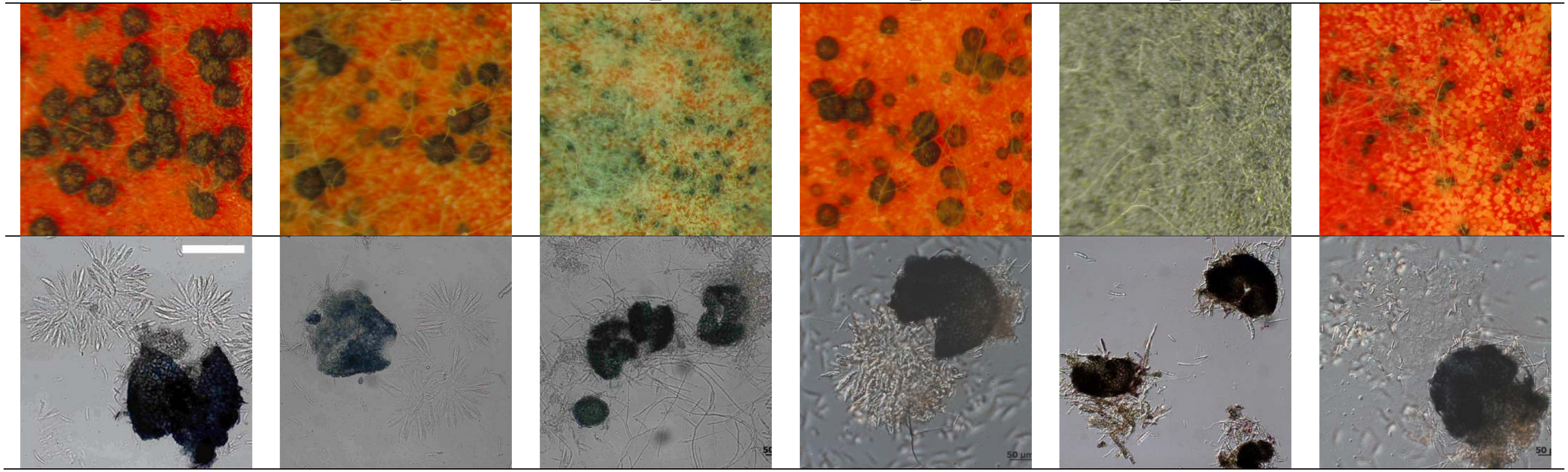

GzJUM003	*GzWing004*	*GzZC109*	*GzZC230*
FGSG_05855	FGSG_01030	FGSG_08626	FGSG_07133
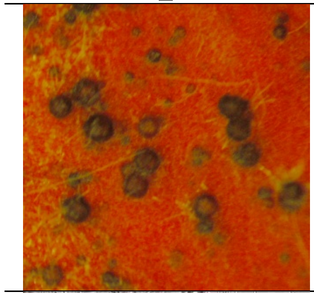	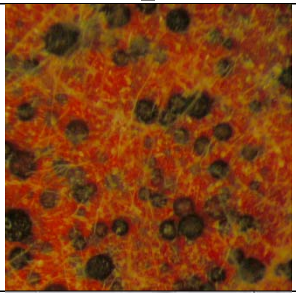	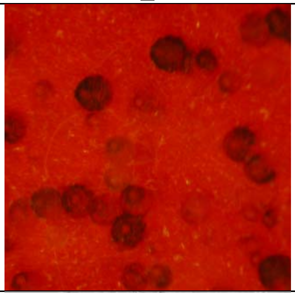	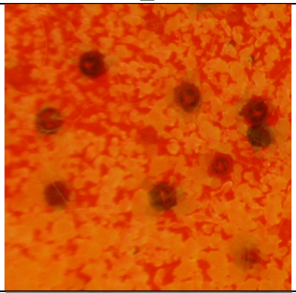
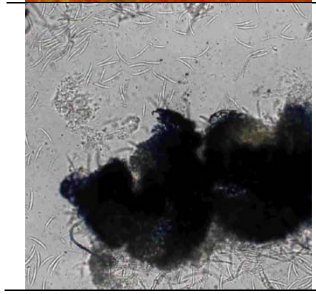	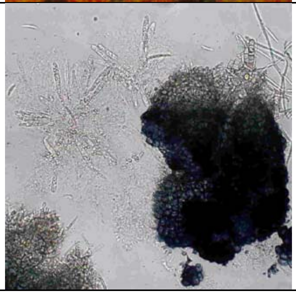	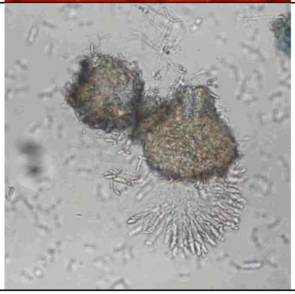	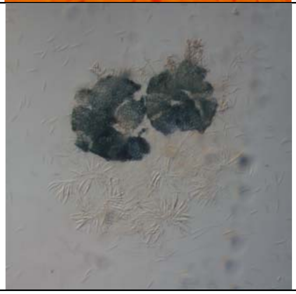

Group 5. Defect in perithecia development and no ascospores (19)

ZIF1	*GzBrlA*	*GzC2H103*	*GzGATA006*	*GzCCAAT002*	*GzHMG010*
FGSG_01555	FGSG_07310	FGSG_13314	FGSG_08634	FGSG_01182	FGSG_01366

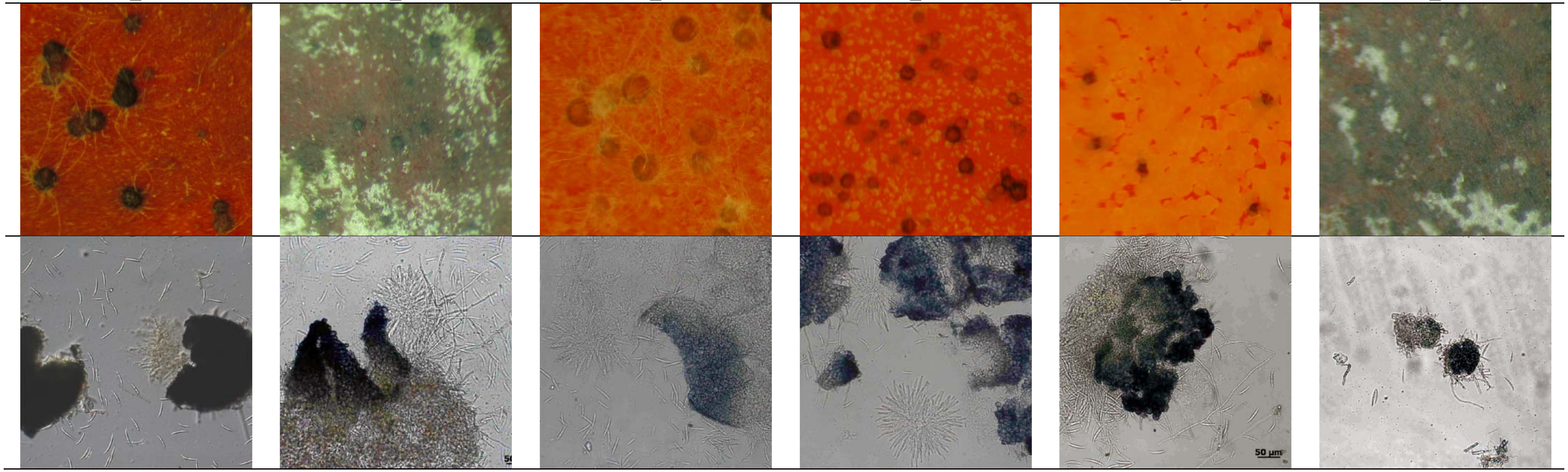

MAT1-1-3	*MAT1-1-1*	*MAT1-2-1*	*GzMADS001*	*MYT1*	*GzOB038*
FGSG_08890	FGSG_08892	FGSG_08893	FGSG_08696	FGSG_00318	FGSG_09654

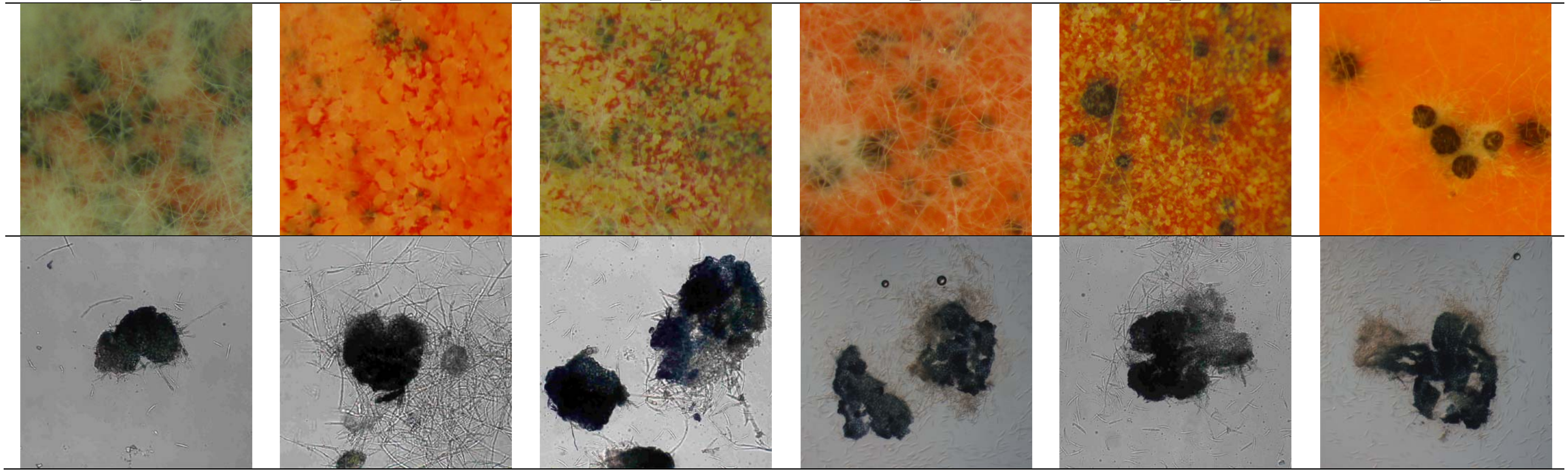

GzFlbA	*GzWing015*	*GzRFX1*	*GzWing027*	*GzZC151*	*GzZC229*
FGSG_06228	FGSG_06944	FGSG_07420	FGSG_11826	FGSG_02445	FGSG_06160

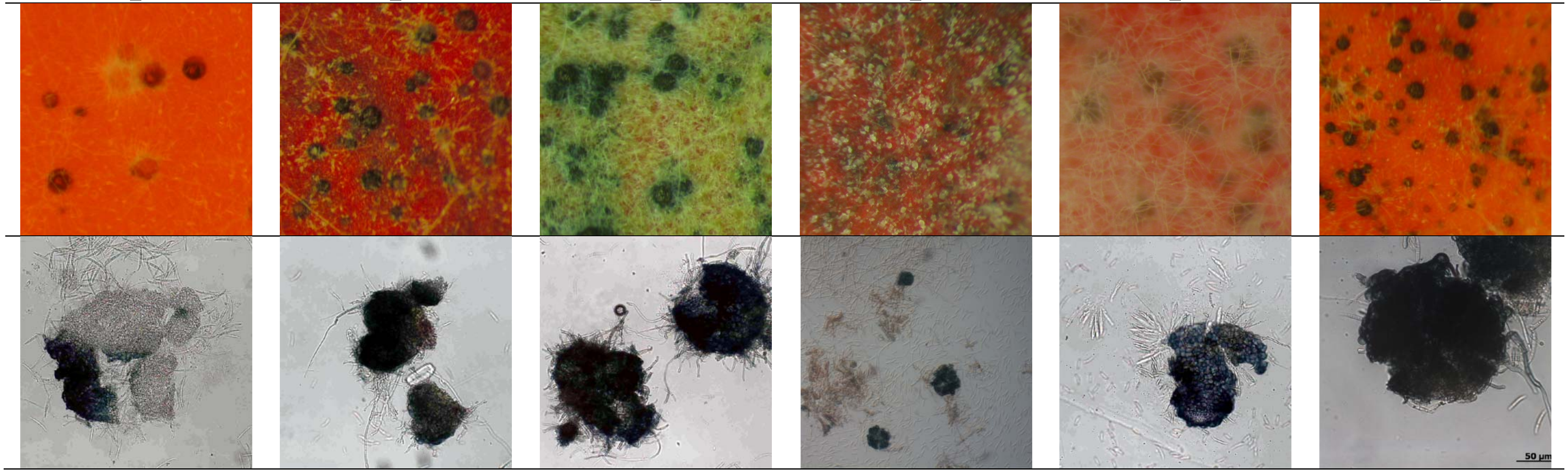

GzZC246
FGSG_02083
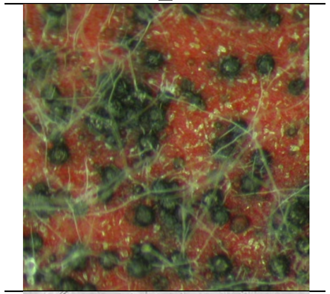
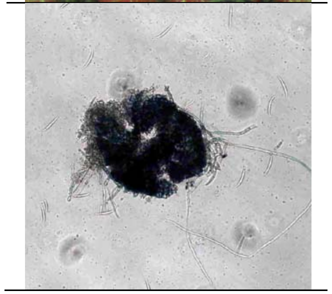

Group 6. Defect in ascospores formation but not in perithecia development (5)

GzHMG004	*GzHMG018*	*GzWing012*	*GzCCHC010*	*GzZC215*
FGSG_00696	FGSG_05604	FGSG_05949	FGSG_10286	FGSG_05503

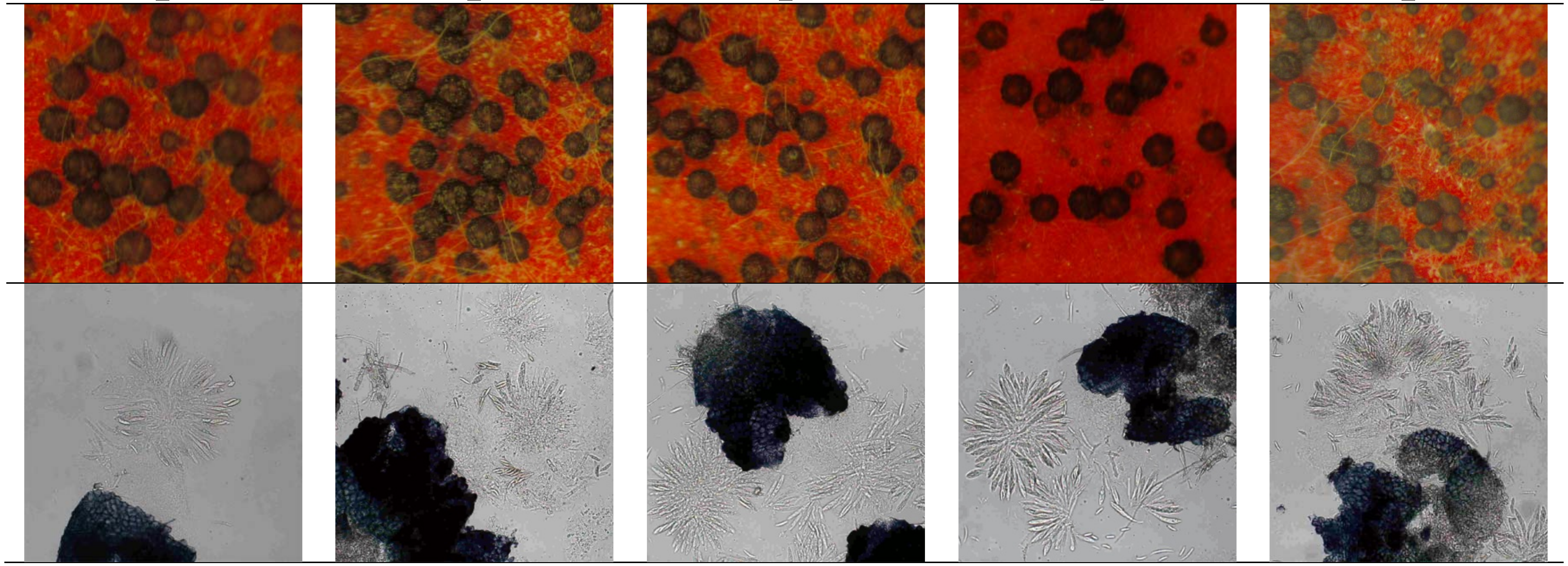

Group 7. Normal perithecia development but no ascospores (1)

GzC2H098
FGSG_12837
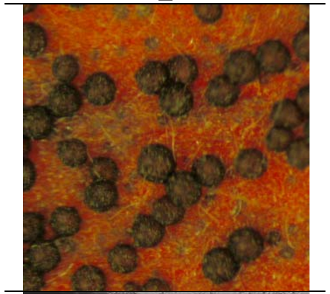
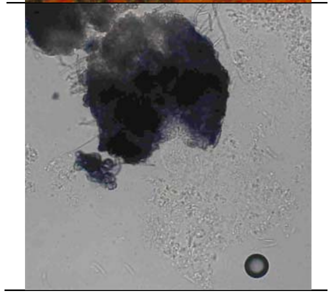
