# Supplementary material for: A Phenome-Based Functional Analysis of Transcription Factors in the Cereal Head Blight Fungus, Fusarium graminearum
Source: PLoS Pathog. 2011 Oct 20;7(10):e1002310. doi: 10.1371/journal.ppat.1002310 (PMC3197617; doi:10.1371/journal.ppat.1002310)

WT	*GzAPSES001*	*FgStuA*	*GzAPSES004*	*GzAT001*	*GzbHLH005*	*GzBrom002*	*GzbZIP001*	*ZIF1*	*GzbZIP007*	*GzbZIP010*
	FGSG_04220	FGSG_10129	FGSG_10384	FGSG_06071	FGSG_01307	FGSG_06291	FGSG_00515	FGSG_01555	FGSG_05171	FGSG_06651

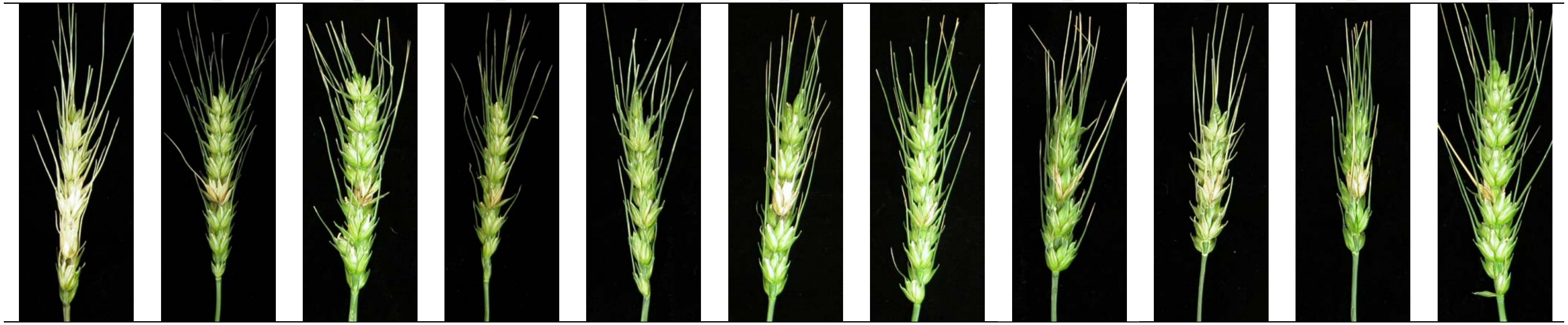

GzbZIP016	*GzbZIP017*	*GzC2H003*	*GzC2H007*	*GzC2H008*	*GzC2H013*	*GzC2H014*	*GzC2H024*	*GzCON7*	*GzC2H042*	*GzC2H045*
FGSG_09832	FGSG_10142	FGSG_00477	FGSG_01022	FGSG_01106	FGSG_01341	FGSG_01350	FGSG_04083	FGSG_04134	FGSG_06427	FGSG_06871

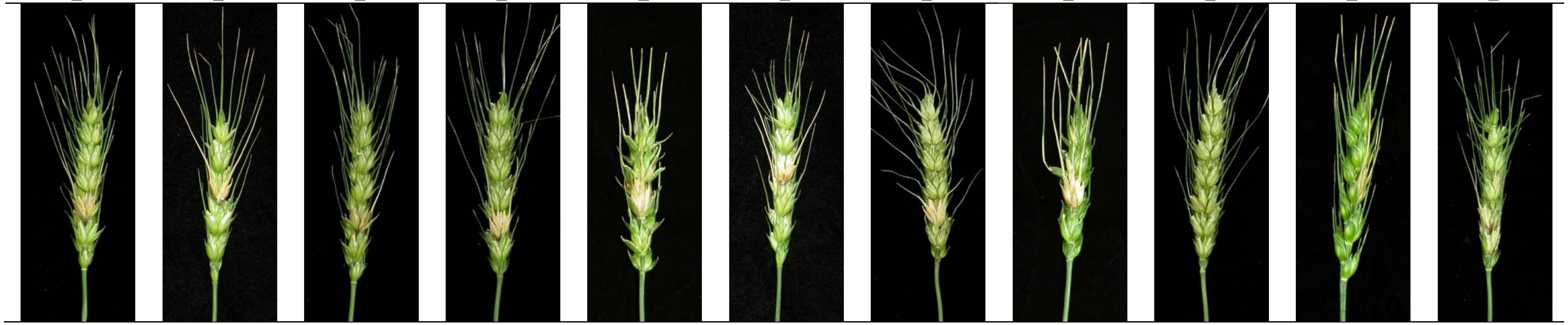

GzC2H059	*GzC2H066*	*GzC2H090*	*GzC2H093*	*GzC2H105*	*GzNot002*	*GzDDT*	*GzCCAAT002*	*GzCCAAT004*	*GzHMG002*	*GzHMG005*
FGSG_07928	FGSG_08617	FGSG_10517	FGSG_11416	FGSG_13711	FGSG_13746	FGSG_02527	FGSG_01182	FGSG_05304	FGSG_00385	FGSG_00729

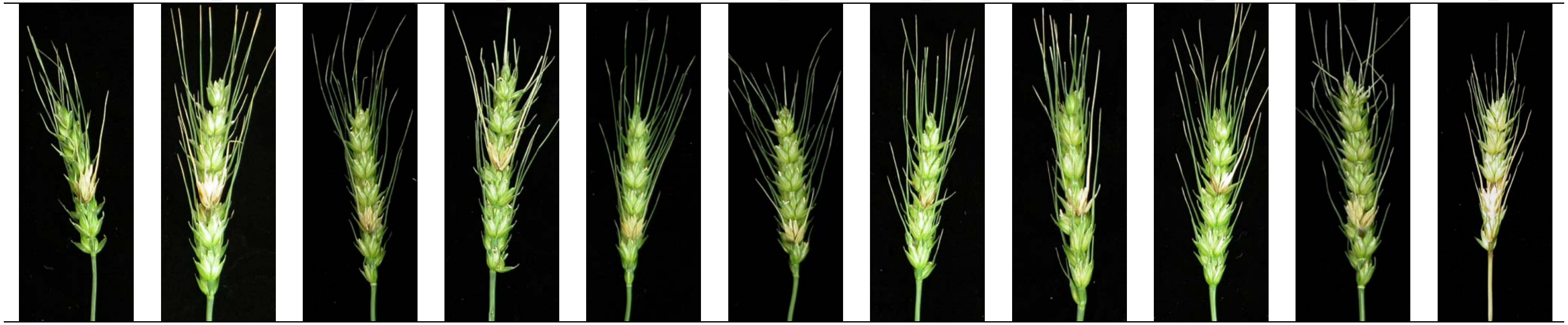

GzHMG029	*GzHOME009*	*Gzscp*	*GzLam002*	*GzMADS003*	*GzMyb002*	*GzMyb017*	*GzNH001*	*GzOB031*	*GzOB038*	*GzOB047*
FGSG_09868	FGSG_09019	FGSG_06948	FGSG_10179	FGSG_09339	FGSG_00324	FGSG_12781	FGSG_09992	FGSG_08737	FGSG_09654	FGSG_13120

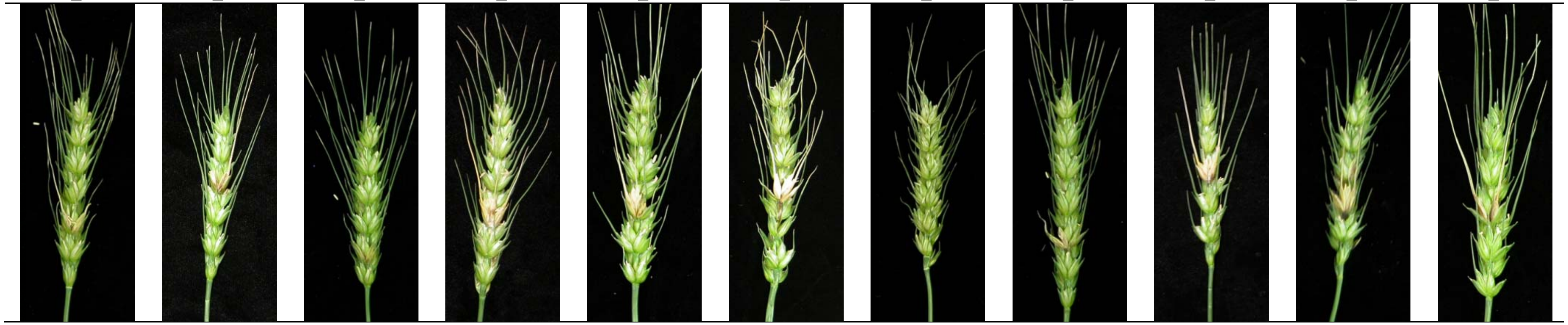

FgFSR1	*GzFlbA*	*GzWing015*	*GzWing018*	*GzWing019*	*GzWing020*	*GzCCHC011*	*GzZC087*	*GzZC108*	*GzZC116*	*GzZC120*
FGSG_05388	FGSG_06228	FGSG_06944	FGSG_08481	FGSG_08572	FGSG_08719	FGSG_10716	FGSG_12977	FGSG_08769	FGSG_08182	FGSG_08028

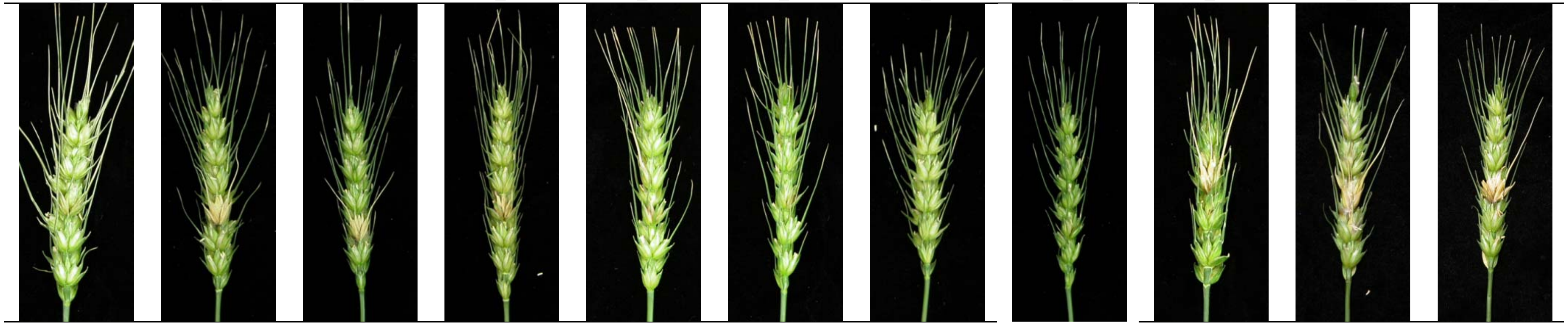

GzZC230	*GzZC232*	*GzZC236*	*GzZC248*	*GzZC282*	*GzZC302*	*GzZC305*
FGSG_07133	FGSG_07067	FGSG_01293	FGSG_01176	FGSG_00719	FGSG_00574	FGSG_00147

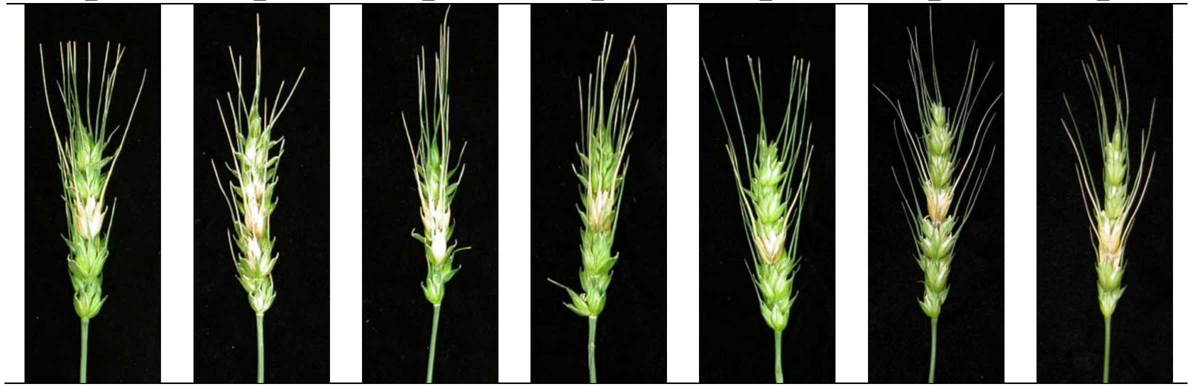

Supplement: Figure S5 — Virulence of TF mutants on wheat heads. A center spikelet of each wheat head was injected with 10 µl of conidia suspension. The photographs were taken 14 days after inoculation. WT, G. zeae wild-type strain GZ3639. (PDF) [file ppat.1002310.s005.pdf]
