# Supplementary material for: A Phenome-Based Functional Analysis of Transcription Factors in the Cereal Head Blight Fungus, Fusarium graminearum
Source: PLoS Pathog. 2011 Oct 20;7(10):e1002310. doi: 10.1371/journal.ppat.1002310 (PMC3197617; doi:10.1371/journal.ppat.1002310)
Supplement: Figure S6 — Toxin production using thin layer chromatography (TLC) analysis. Fungal strains were grown on rice substrate for three weeks. ZEA and DON are indicated in TLC plate image of the wild-type strain. WT, G. zeae wild-type strain GZ3639. ZEA, zearalenone; DON, deoxynivalenol. (PDF) [file ppat.1002310.s006.pdf]

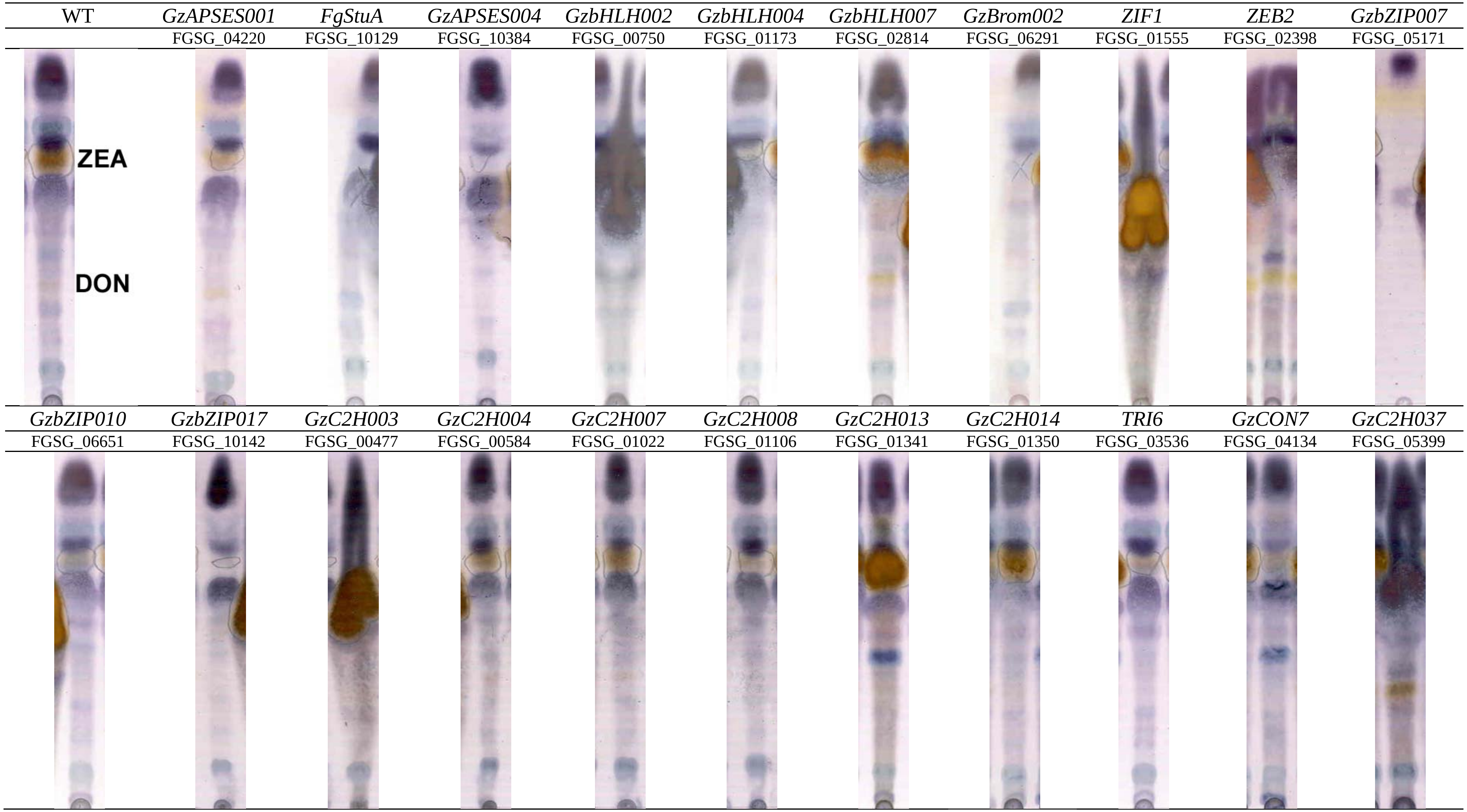
WT
GzAPSES001 FGSG_04220
FgStuA FGSG_10129
GzAPSES004 FGSG_10384
GzbHLH002 FGSG_00750
GzbHLH004 FGSG_01173
GzbHLH007 FGSG_02814
GzBrom002 FGSG_06291
ZIF1 FGSG_01555
ZEB2 FGSG_02398
GzbZIP007 FGSG_05171
ZEA
DON
GzbZIP010 FGSG_06651
GzbZIP017 FGSG_10142
GzC2H003 FGSG_00477
GzC2H004 FGSG_00584
GzC2H007 FGSG_01022
GzC2H008 FGSG_01106
GzC2H013 FGSG_01341
GzC2H014 FGSG_01350
TRI6 FGSG_03536
GzCON7 FGSG_04134
GzC2H037 FGSG_05399

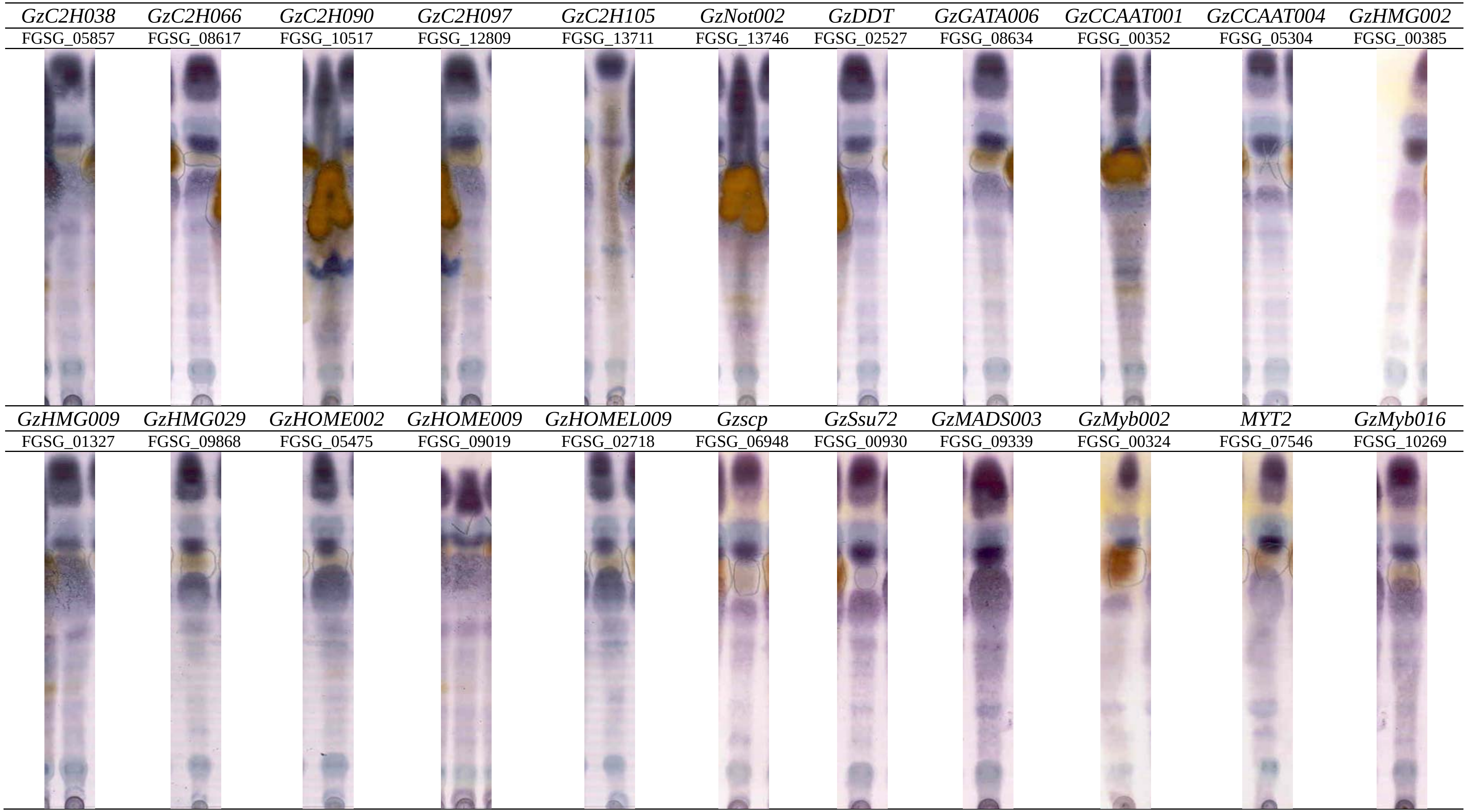

GzC2H038
FGSG_05857
GzC2H066
FGSG_08617
GzC2H090
FGSG_10517
GzC2H097
FGSG_12809
GzC2H105
FGSG_13711
GzNot002
FGSG_13746
GzDDT
FGSG_02527
GzGATA006
FGSG_08634
GzCCAAT001
FGSG_00352
GzCCAAT004
FGSG_05304
GzHMG002
FGSG_00385
GzHMG009
FGSG_01327
GzHMG029
FGSG_09868
GzHOME002
FGSG_05475
GzHOME009
FGSG_09019
GzHOMEL009
FGSG_02718
Gzscp
FGSG_06948
GzSsu72
FGSG_00930
GzMADS003
FGSG_09339
GzMyb002
FGSG_00324
MYT2
FGSG_07546
GzMyb016
FGSG_10269

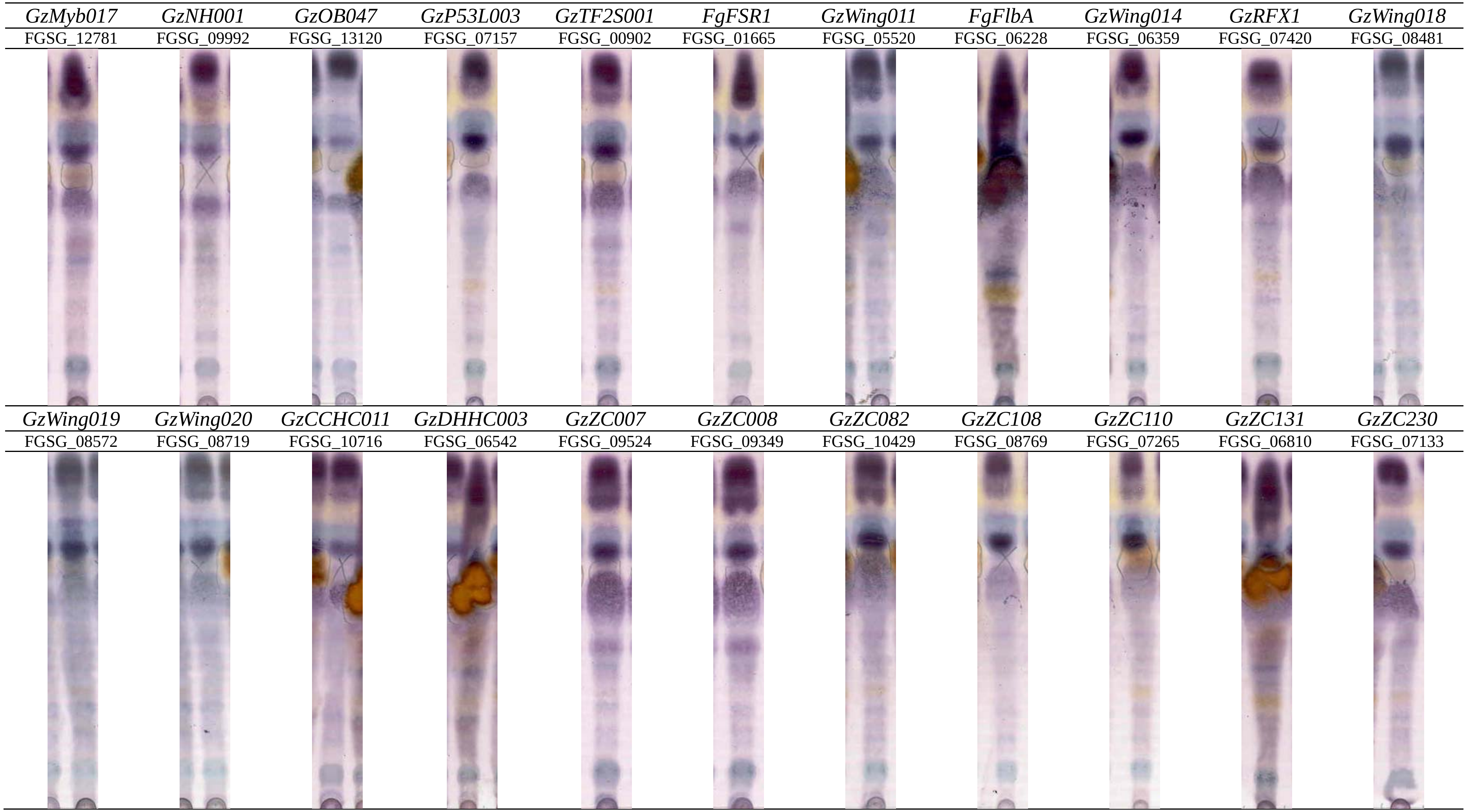
GzMyb017
FGSG_12781
GzNH001
FGSG_09992
GzOB047
FGSG_13120
GzP53L003
FGSG_07157
GzTF2S001
FGSG_00902
FgFSR1
FGSG_01665
GzWing011
FGSG_05520
FgFlbA
FGSG_06228
GzWing014
FGSG_06359
GzRFX1
FGSG_07420
GzWing018
FGSG_08481
GzWing019
FGSG_08572
GzWing020
FGSG_08719
GzCCHC011
FGSG_10716
GzDHHC003
FGSG_06542
GzZC007
FGSG_09524
GzZC008
FGSG_09349
GzZC082
FGSG_10429
GzZC108
FGSG_08769
GzZC110
FGSG_07265
GzZC131
FGSG_06810
GzZC230
FGSG_07133

GzZC232	*GzZC233*	*GzZC246*	*GzZC247*	*GzZC248*	*GzZC250*
FGSG_07067	FGSG_07368	FGSG_02083	FGSG_02068	FGSG_01176	FGSG_02531
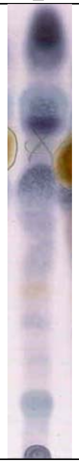	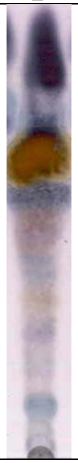	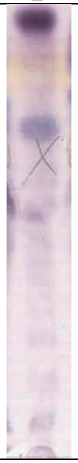	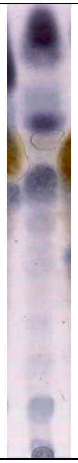	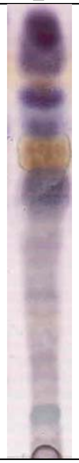	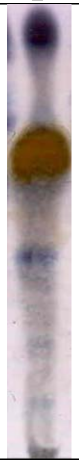
