# Supplementary material for: A Phenome-Based Functional Analysis of Transcription Factors in the Cereal Head Blight Fungus, Fusarium graminearum
Source: PLoS Pathog. 2011 Oct 20;7(10):e1002310. doi: 10.1371/journal.ppat.1002310 (PMC3197617; doi:10.1371/journal.ppat.1002310)
Supplement: Figure S7 — Phenotype of TF mutants under various stress conditions. The photographs were taken three days after inoculation. WT, G. zeae wild-type strain GZ3639. CM, mock complete medium; NaCl, CM with 1 M NaCl; KCl, CM with 1 M KCl; Sorbitol, CM with 1.5 M sorbitol; FeSO4, CM with 6 mM FeSO4; H2O2, CM with 5 mM; Mena, CM with 0.1 mM menadione; Fludi, CM with 0.023 mg/L fludioxonil; Ipro, CM with 8.6 mg/L iprodione; SDS, sodium dodecyl sulfate (SDS) 5 mg/L; C.R., Congo Red 60 mg/L; pH 4 and pH 11, CM with pH = 4 and pH = 11, respectively. (PDF) [file ppat.1002310.s007.pdf]

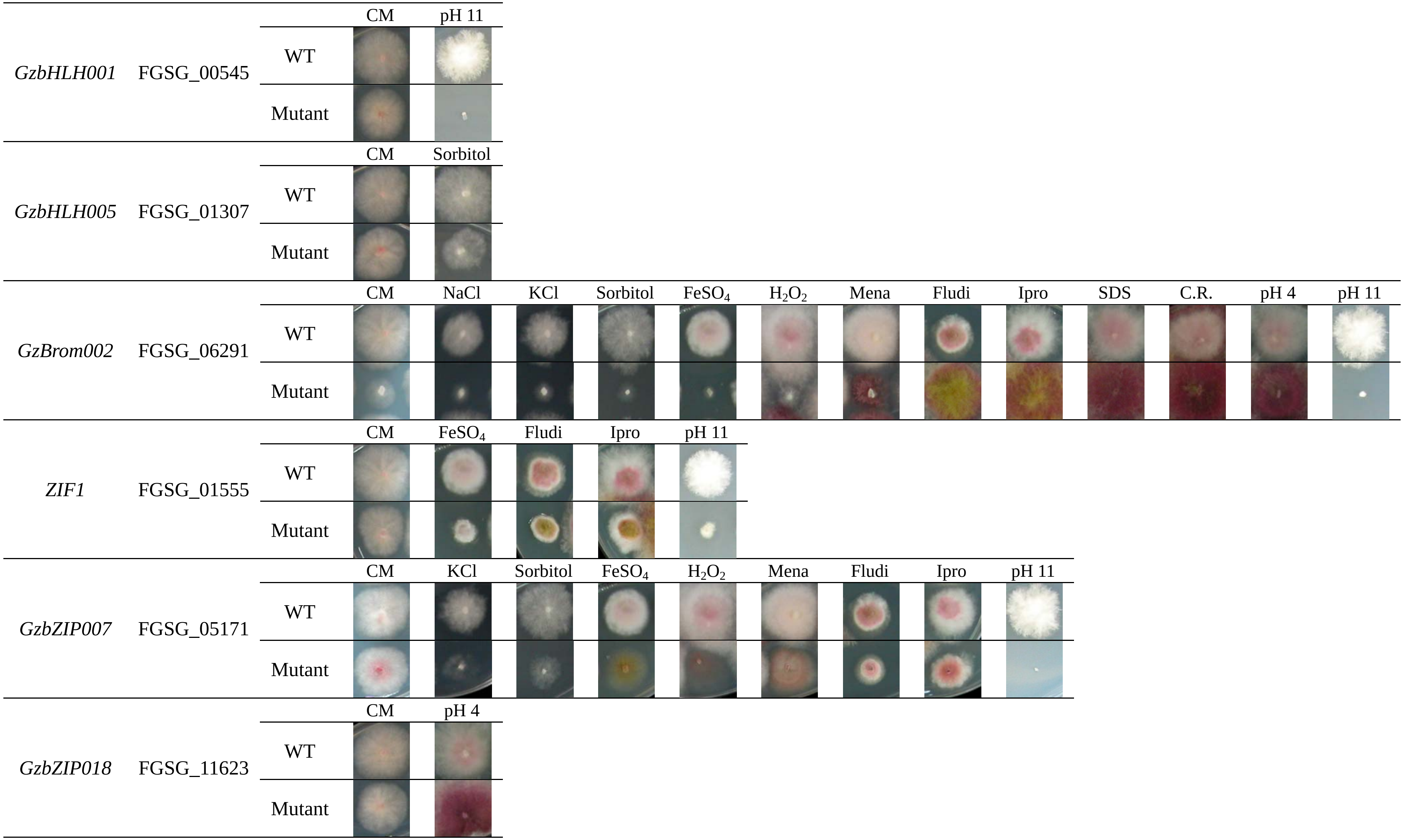
GzbHLH001
FGSG_00545
CM
pH 11
WT
Mutant
GzbHLH005
FGSG_01307
CM
Sorbitol
WT
Mutant
GzBrom002
FGSG_06291
CM
NaCl
KCl
Sorbitol
$FeSO_4$
H_2O_2
Mena
Fludi
Ipro
SDS
C.R.
pH 4
pH 11
WT
Mutant
ZIF1
FGSG_01555
CM
$FeSO_4$
Fludi
Ipro
pH 11
WT
Mutant
GzbZIP007
FGSG_05171
CM
KCl
Sorbitol
$FeSO_4$
H_2O_2
Mena
Fludi
Ipro
pH 11
WT
Mutant
GzbZIP018
FGSG_11623
CM
pH 4
WT
Mutant

			CM	pH 4	
GzC2H005	FGSG_00653	WT	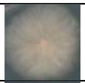	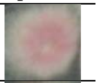	
		Mutant	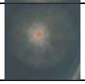	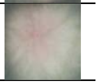	
			CM	pH 11	
GzC2H007	FGSG_01022	WT	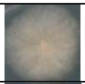	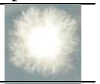	
		Mutant	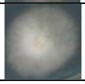	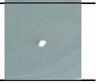	
			CM	pH 11	
GzC2H013	FGSG_01341	WT	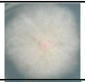	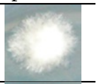	
		Mutant	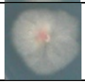	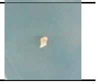	
			CM	pH 11	
GzC2H020	FGSG_02803	WT	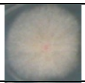	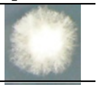	
		Mutant	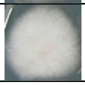	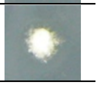	
			CM	pH 11	
GzC2H036	FGSG_05381	WT	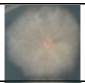	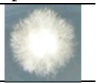	
		Mutant	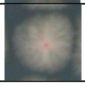	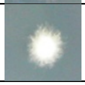	
			CM	$FeSO_4$	pH 11
GzC2H048	FGSG_07075	WT	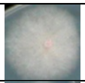	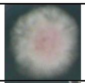	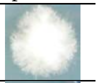
		Mutant	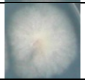	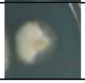	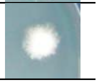

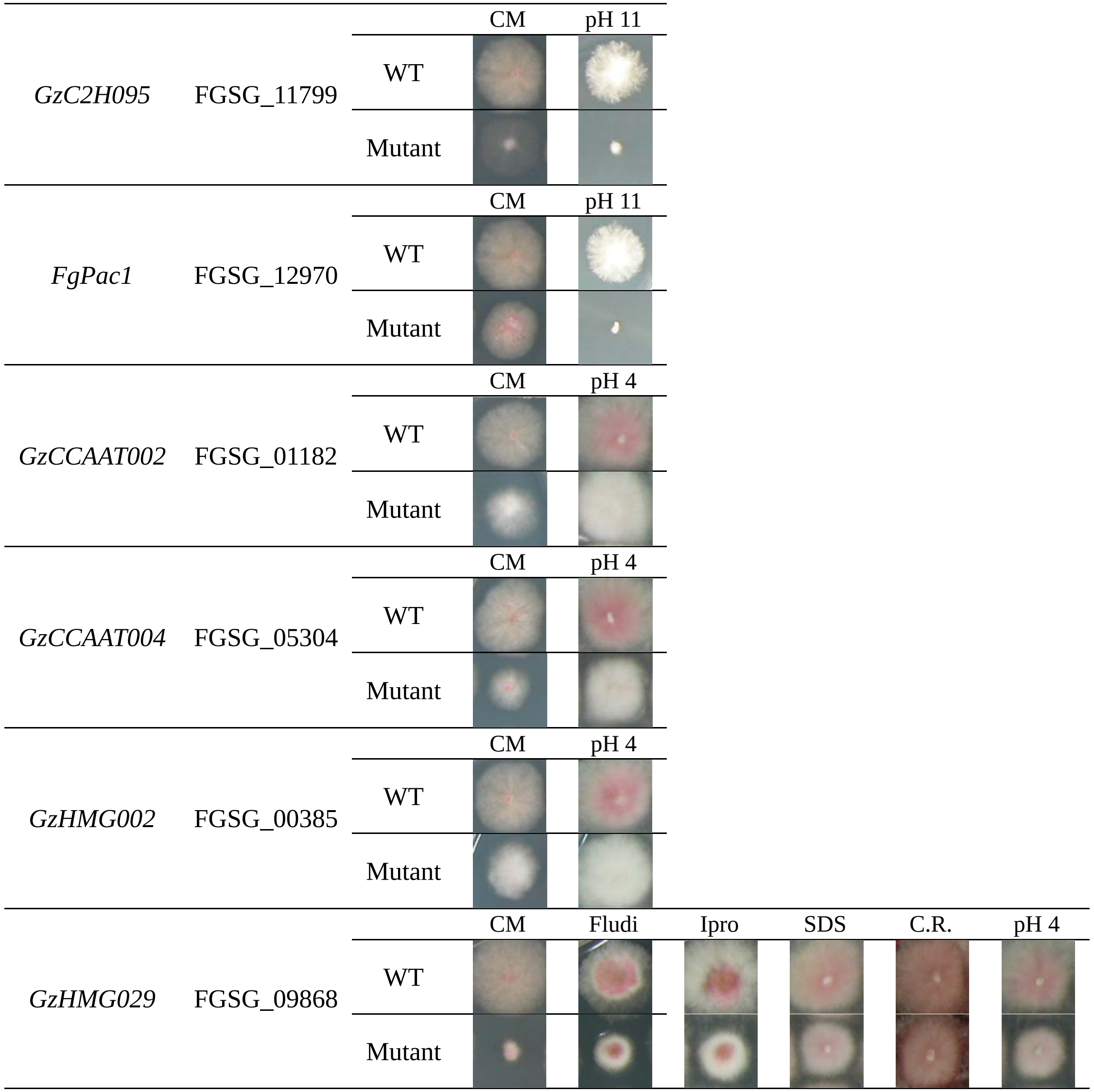
CM
pH 11
GzC2H095
FGSG_11799
WT
Mutant
CM
pH 11
FgPac1
FGSG_12970
WT
Mutant
CM
pH 4
GzCCAAT002
FGSG_01182
WT
Mutant
CM
pH 4
GzCCAAT004
FGSG_05304
WT
Mutant
CM
pH 4
GzHMG002
FGSG_00385
WT
Mutant
CM
Fludi
Ipro
SDS
C.R.
pH 4
GzHMG029
FGSG_09868
WT
Mutant

GzSsu72	FGSG_00930		CM	$FeSO_4$	SDS		
		WT	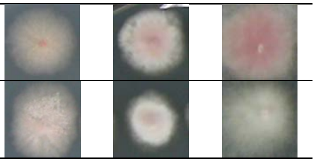				
		Mutant					
GzLam002	FGSG_10179		CM	Fludi			
		WT	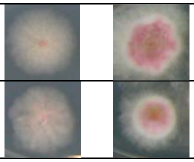				
		Mutant					
GzMADS001	FGSG_08696		CM	$FeSO_4$	SDS		
		WT	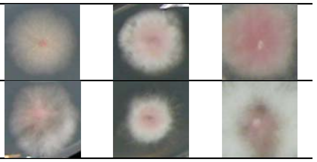				
		Mutant					
MYT1	FGSG_00318		CM	pH 11			
		WT	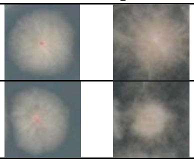				
		Mutant					
GzMyb002	FGSG_00324		CM	NaCl	KCl	Sorbitol	
		WT	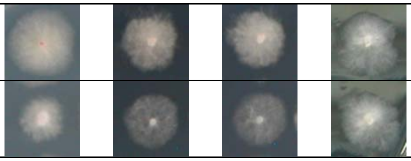				
		Mutant					
GzMyb008	FGSG_02719		CM	Ipro	SDS	C.R.	pH 4
		WT	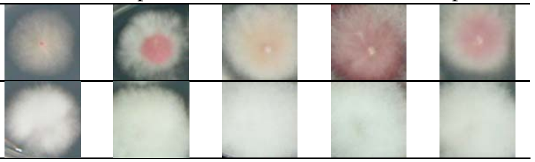				
		Mutant					

			CM	Fludi	
GzOB042	FGSG_09904	WT	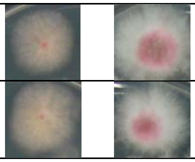		
		Mutant			
			CM	NaCl	KCl
GzP53L002	FGSG_03874	WT	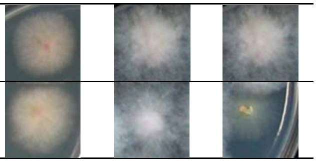		
		Mutant			
			CM	Fludi	Ipro
GzOpi	FGSG_08981	WT	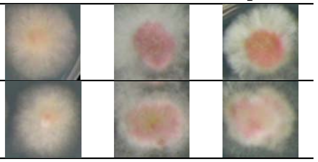		
		Mutant			
			CM	NaOH	
GzTF2S001	FGSG_00902	WT	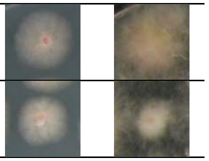		
		Mutant			
			CM	pH 4	
FgFSR1	FGSG_01665	WT	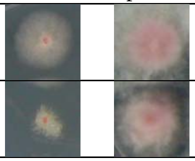		
		Mutant			
			CM	$FeSO_4$	SDS
GzWing011	FGSG_05520	WT	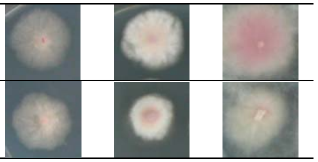		
		Mutant			

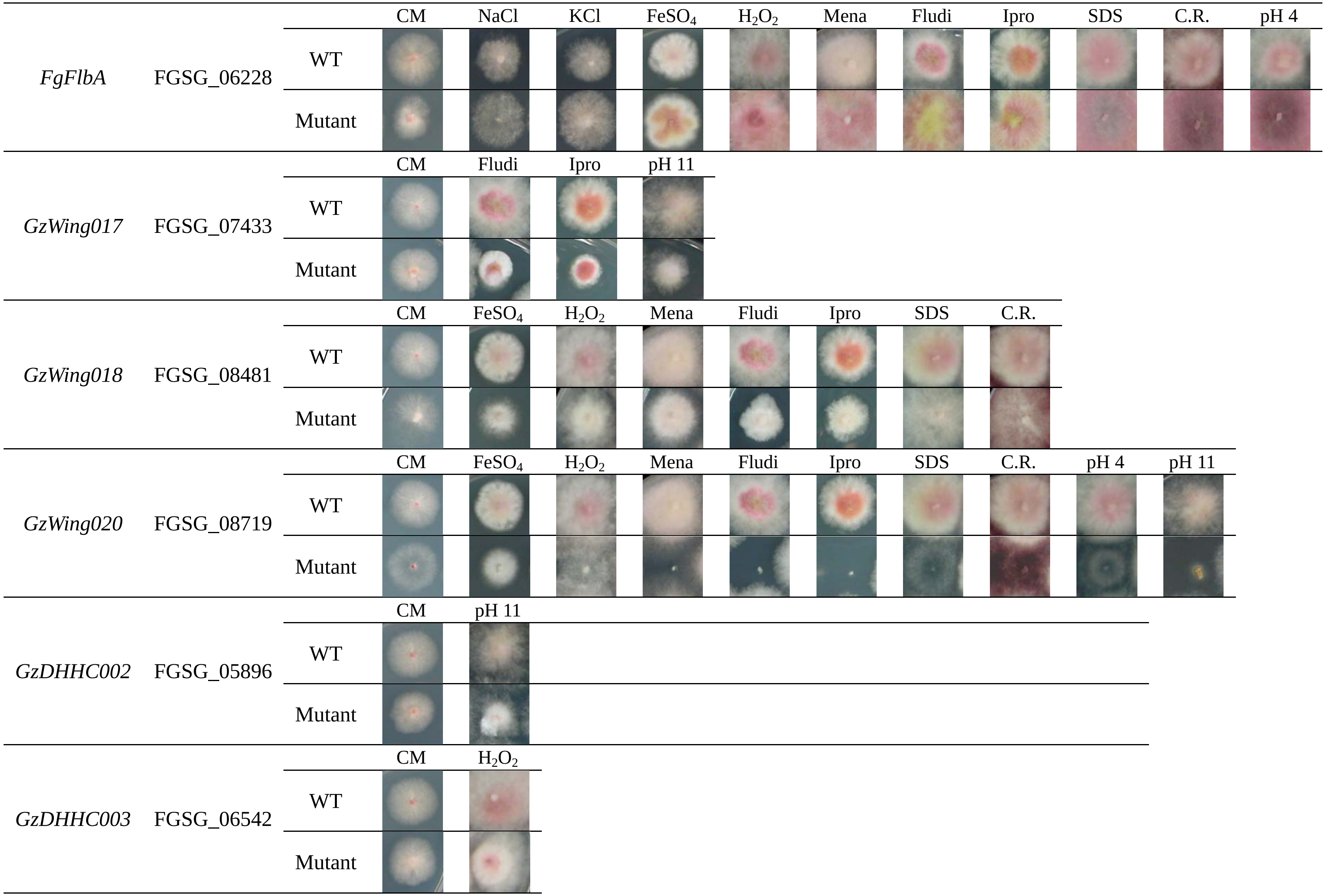

CM
NaCl
KCl
FeSO4
H2O2
Mena
Fludi
Ipro
SDS
C.R.
pH 4
FgFlbA
FGSG_06228
WT
Mutant
CM
Fludi
Ipro
pH 11
GzWing017
FGSG_07433
WT
Mutant
CM
FeSO4
H2O2
Mena
Fludi
Ipro
SDS
C.R.
GzWing018
FGSG_08481
WT
Mutant
CM
FeSO4
H2O2
Mena
Fludi
Ipro
SDS
C.R.
pH 4
pH 11
GzWing020
FGSG_08719
WT
Mutant
CM
pH 11
GzDHHC002
FGSG_05896
WT
Mutant
CM
H2O2
GzDHHC003
FGSG_06542
WT
Mutant

Gene	Locus	Strain	Conditions
GzZC083	FGSG_00568		CM, Ipro
		WT	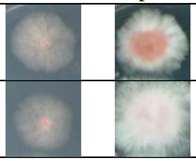
		Mutant	
GzZC086	FGSG_08924		CM, Fludi, Ipro
		WT	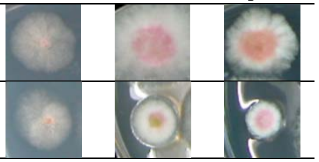
		Mutant	
GzZC113	FGSG_07177		CM, NaCl
		WT	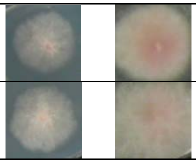
		Mutant	
GzZC121	FGSG_07927		CM, NaCl, KCl, H_2O_2
		WT	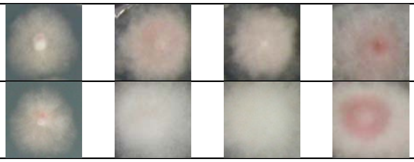
		Mutant	
GzZC162	FGSG_09464		CM, NaCl, KCl, SDS, C.R., pH 4
		WT	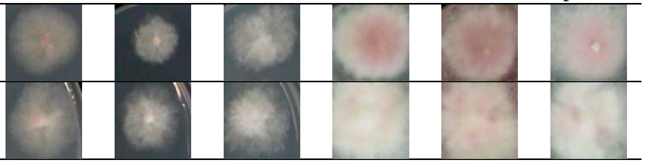
		Mutant	
GzZC172	FGSG_11364		CM, pH 11
		WT	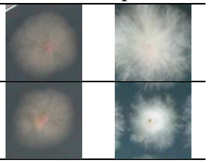
		Mutant	

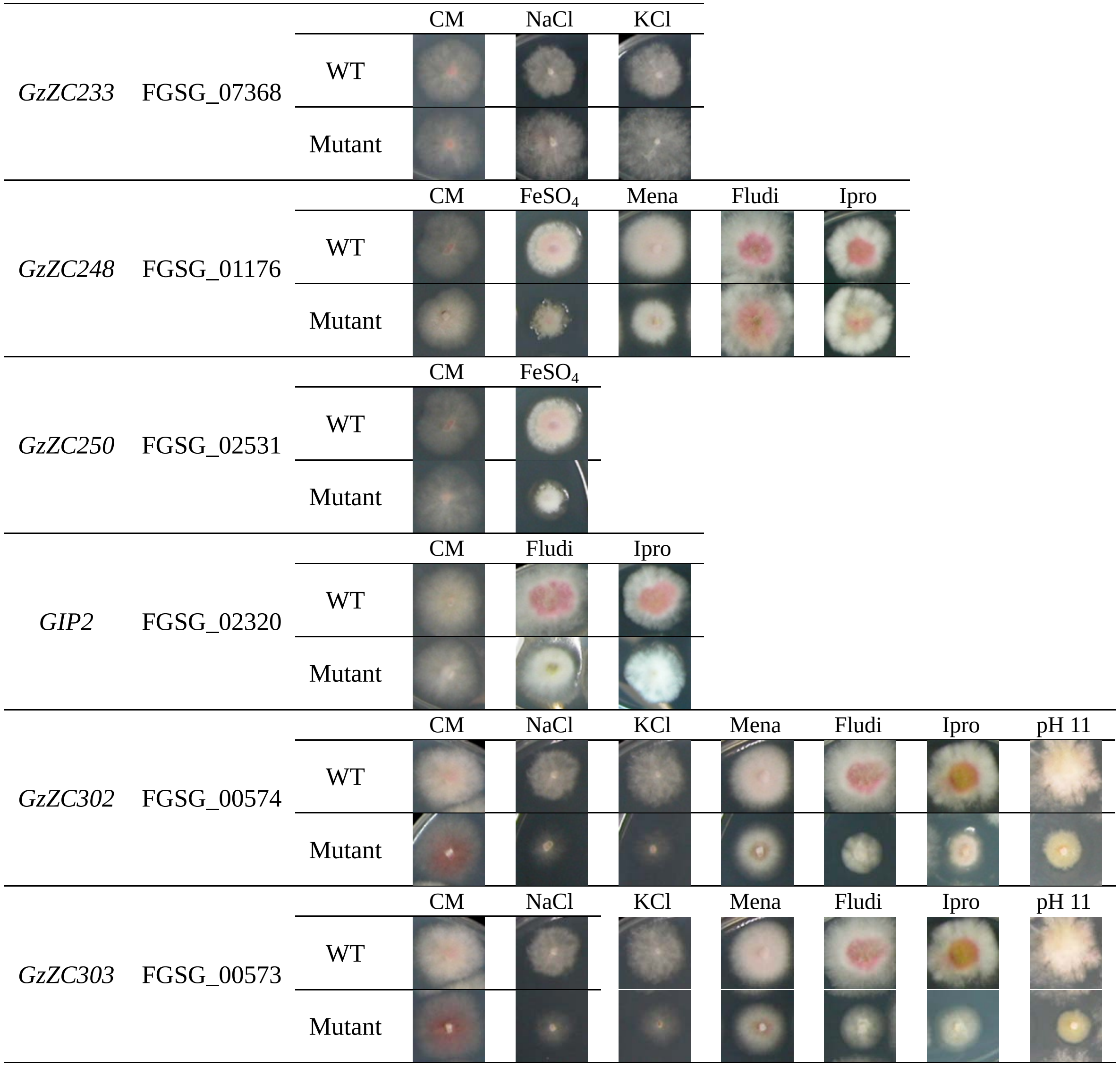

GzZC233 FGSG_07368
CM NaCl KCl
WT
Mutant
GzZC248 FGSG_01176
CM FeSO4 Mena Fludi Ipro
WT
Mutant
GzZC250 FGSG_02531
CM FeSO4
WT
Mutant
GIP2 FGSG_02320
CM Fludi Ipro
WT
Mutant
GzZC302 FGSG_00574
CM NaCl KCl Mena Fludi Ipro pH 11
WT
Mutant
GzZC303 FGSG_00573
CM NaCl KCl Mena Fludi Ipro pH 11
WT
Mutant

			CM	SDS	C.R.
GzZC305	FGSG_00147	WT	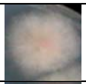	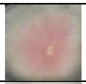	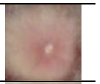
		Mutant	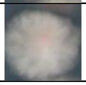	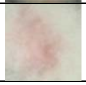	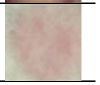
