# Supplementary material for: A Phenome-Based Functional Analysis of Transcription Factors in the Cereal Head Blight Fungus, Fusarium graminearum
Source: PLoS Pathog. 2011 Oct 20;7(10):e1002310. doi: 10.1371/journal.ppat.1002310 (PMC3197617; doi:10.1371/journal.ppat.1002310)
Supplement: Figure S8 — Gene expression patterns of transcription factors, exhibiting defective sexual phenotypes in a deletion mutant, from perithecia development-related microarray data. 105 defective sexual phenotypes were grouped (Table S8 and Figure 3) and two microarray experiments were employed to monitor expression patterns; one is a sexual development microarray on G. zeae wild-type strain PH-1 (from 0 h to 144 h) [5] and the other is a microarray on ascospore discharge of PH-1 mutant [6]. (PDF) [file ppat.1002310.s008.pdf]

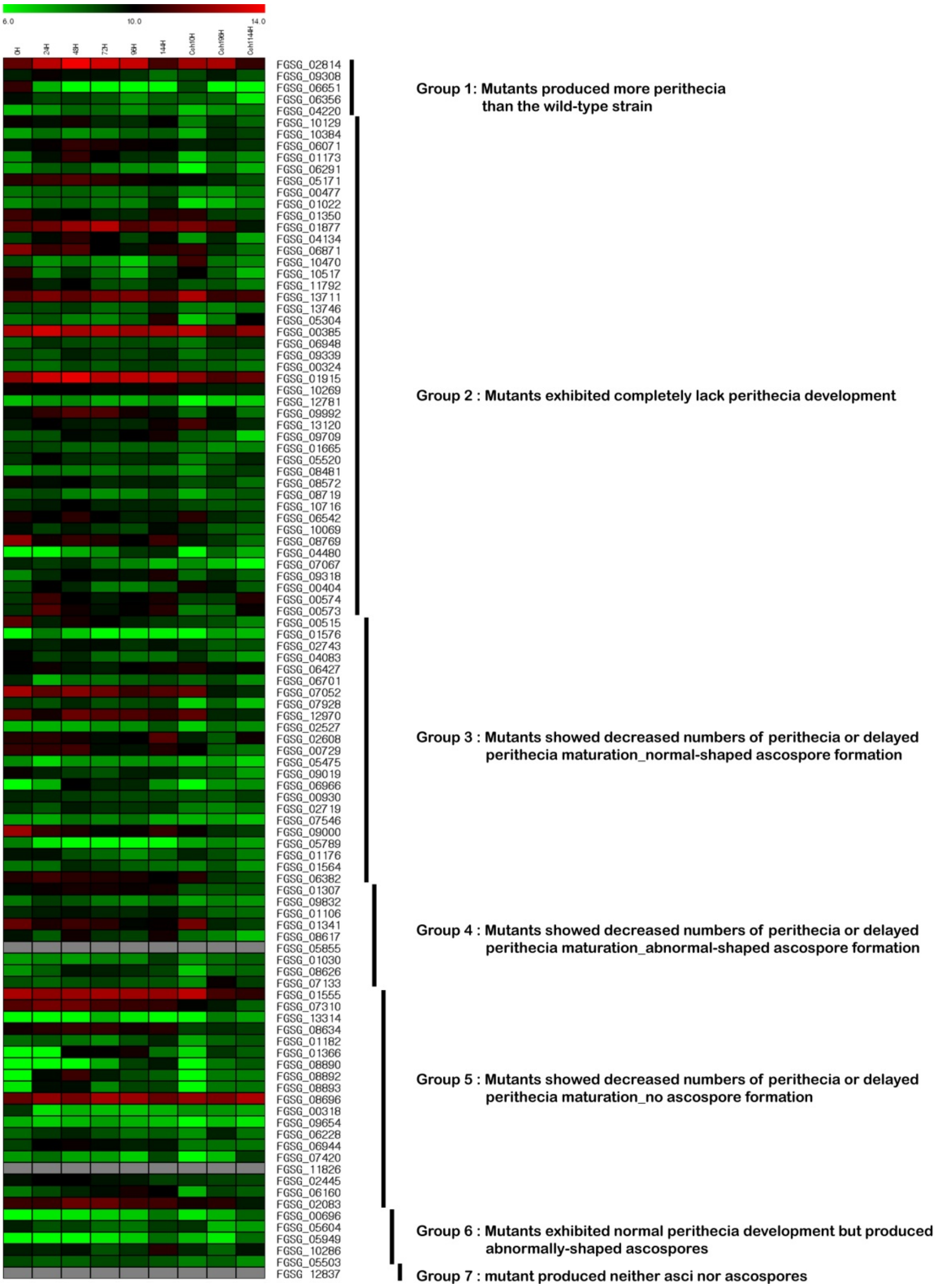

6.0
10.0
14.0
0H
24H
48H
72H
96H
144H
FGSG_02814
FGSG_09308
FGSG_06651
FGSG_06356
FGSG_04220
FGSG_10129
FGSG_10384
FGSG_06071
FGSG_01173
FGSG_06291
FGSG_05171
FGSG_00477
FGSG_01022
FGSG_01350
FGSG_01877
FGSG_04134
FGSG_06871
FGSG_10470
FGSG_10517
FGSG_11792
FGSG_13711
FGSG_13746
FGSG_05304
FGSG_00385
FGSG_06948
FGSG_09339
FGSG_00324
FGSG_01915
FGSG_10269
FGSG_12781
FGSG_09992
FGSG_13120
FGSG_09709
FGSG_01665
FGSG_05520
FGSG_08481
FGSG_08572
FGSG_08719
FGSG_10716
FGSG_06542
FGSG_10069
FGSG_08769
FGSG_04480
FGSG_07067
FGSG_09318
FGSG_00404
FGSG_00574
FGSG_00573
FGSG_00515
FGSG_01576
FGSG_02743
FGSG_04083
FGSG_06427
FGSG_06701
FGSG_07052
FGSG_07928
FGSG_12970
FGSG_02527
FGSG_02608
FGSG_00729
FGSG_05475
FGSG_09019
FGSG_06966
FGSG_00930
FGSG_02719
FGSG_07546
FGSG_09000
FGSG_05789
FGSG_01176
FGSG_01564
FGSG_06382
FGSG_01307
FGSG_09832
FGSG_01106
FGSG_01341
FGSG_08617
FGSG_05855
FGSG_01030
FGSG_08626
FGSG_07133
FGSG_01555
FGSG_07310
FGSG_13314
FGSG_08634
FGSG_01182
FGSG_01366
FGSG_08890
FGSG_08892
FGSG_08893
FGSG_08696
FGSG_00318
FGSG_09654
FGSG_06228
FGSG_06944
FGSG_07420
FGSG_11826
FGSG_02445
FGSG_06160
FGSG_02083
FGSG_00696
FGSG_05604
FGSG_05949
FGSG_10286
FGSG_05503
FGSG_12837
Group 1: Mutants produced more perithecia than the wild-type strain
Group 2 : Mutants exhibited completely lack perithecia development
Group 3 : Mutants showed decreased numbers of perithecia or delayed perithecia maturation_normal-shaped ascospore formation
Group 4 : Mutants showed decreased numbers of perithecia or delayed perithecia maturation_abnormal-shaped ascospore formation
Group 5 : Mutants showed decreased numbers of perithecia or delayed perithecia maturation_no ascospore formation
Group 6 : Mutants exhibited normal perithecia development but produced abnormally-shaped ascospores
Group 7 : mutant produced neither asci nor ascospores
